# U-Sleep: resilient high-frequency sleep staging

**DOI:** 10.1038/s41746-021-00440-5

**Published:** 2021-04-15

**Authors:** Mathias Perslev, Sune Darkner, Lykke Kempfner, Miki Nikolic, Poul Jørgen Jennum, Christian Igel

**Affiliations:** 1 Department of Computer Science, University of Copenhagen, Copenhagen, Denmark; 2 Danish Center for Sleep Medicine, Rigshospitalet, Copenhagen, Denmark

**Keywords:** Sleep, Machine learning, Software, Electroencephalography - EEG

## Abstract

Sleep disorders affect a large portion of the global population and are strong predictors of morbidity and all-cause mortality. Sleep staging segments a period of sleep into a sequence of phases providing the basis for most clinical decisions in sleep medicine. Manual sleep staging is difficult and time-consuming as experts must evaluate hours of polysomnography (PSG) recordings with electroencephalography (EEG) and electrooculography (EOG) data for each patient. Here, we present U-Sleep, a publicly available, ready-to-use deep-learning-based system for automated sleep staging (sleep.ai.ku.dk). U-Sleep is a fully convolutional neural network, which was trained and evaluated on PSG recordings from 15,660 participants of 16 clinical studies. It provides accurate segmentations across a wide range of patient cohorts and PSG protocols not considered when building the system. U-Sleep works for arbitrary combinations of typical EEG and EOG channels, and its special deep learning architecture can label sleep stages at shorter intervals than the typical 30 s periods used during training. We show that these labels can provide additional diagnostic information and lead to new ways of analyzing sleep. U-Sleep performs on par with state-of-the-art automatic sleep staging systems on multiple clinical datasets, even if the other systems were built specifically for the particular data. A comparison with consensus-scores from a previously unseen clinic shows that U-Sleep performs as accurately as the best of the human experts. U-Sleep can support the sleep staging workflow of medical experts, which decreases healthcare costs, and can provide highly accurate segmentations when human expertize is lacking.

## Introduction

Sleep disorders affect a large portion of the global population and impose significant welfare costs^1–5^. Abnormal sleeping patterns and associated sleep disorders are strong predictors of morbidity and all-cause mortality^6,7^. Anomalous sleep-wake changes occur for instance in psychiatric conditions (e.g., schizophrenia, depression^8^), neurodegenerative diseases (e.g., dementia, rapid eye movement (REM) sleep behavior disorder, and Parkinson’s Disease^8–10^), and genuine sleep disorders (e.g., narcolepsy^11^, insomnia^12^, sleep apnea^13^) as well as during epileptic seizures and prior to stroke^14^. Timely and accurate diagnosis of sleep disorders relies on the difficult and time-consuming process of sleep staging based on polysomnography (PSG) data. A PSG collects a set of non-invasive long-term recordings of physiological measures of multiple brain and body functions using modalities such as electroencephalography (EEG), electrooculography (EOG), and electromyography (EMG). These signals are divided into intervals, typically of 30 s, which are mapped to different sleep stages such as awake, light sleep, intermediate sleep, deep sleep, and REM sleep^15,16^ (see Supplementary Fig. 2 and Supplementary Table 1 for a brief overview of PSG and sleep stage characteristics). This sleep staging forms the basis for subsequent analyses.

Sleep staging requires multiple hours of manual annotations from expert clinicians for each subject incurring significant costs and leading to bottlenecks in both diagnosis and large-scale clinical studies. The manual annotations suffer from high intra- and interscorer variability, which reduces the diagnostic precision^17,18^. Algorithmic sleep staging aims at automating this process. Recent work shows that such systems can be highly accurate and robust and may play an important role in developing novel biomarkers for sleep disorders and other (e.g., neurodegenerative) diseases^11,19–22^. Deep learning^23^ is becoming increasingly popular for the analysis of physiological time-series in general^24^ and has already been successfully applied to sleep staging^25–27^. While several high-performance deep-learning-based sleep staging systems have been proposed recently^28–38^, these have not yet been widely adopted in clinical practice because it is not clear if the reported results can be generalized. Current state-of-the-art systems are tuned, trained and evaluated on one or a very small number of clinical cohorts, and it remains questionable if similar results can be achieved in a different clinical setting for different patient populations. Most systems are designed to operate on PSG data from a specific hardware & pre-processing pipeline including a specific set of EEG/EOG/EMG channels, sampling rate, etc. to maximize performance. Consequently, most existing sleep staging systems—including deep learning systems trained on several datasets^32,39^—require re-training at each clinical site, which imposes a significant technical barrier.

A robust, easy-to-use sleep staging model directly applicable across clinical populations and PSG protocols with (at least) expert-level performance would both free significant resources across sleep clinics and enable developing countries with advanced sleep diagnostics. Such a system may also serve as a global, standardized reference for sleep staging which could spark scientific discussions and reduce inter-clinical and inter-operator variability.

This study describes U-Sleep, our contribution toward these goals. U-Sleep is a publicly available, ready-to-use deep neural network for resilient sleep staging inspired by the popular U-Net^40–42^ architecture for image segmentation. The neural network was trained and evaluated on the—to the best of our knowledge—largest and most diverse set of PSG records for sleep staging ever collected, spanning 16 independent clinical studies providing 23 datasets, geographically dispersed clinical sites, multiple decades, a large array of demographics, and patient groups. Eight datasets were not considered during model development and training, they were only used for realistic verification of U-Sleep. Two datasets are consensus-scored and allowed us to compare U-Sleep’s performance to that of five clinical experts on both healthy subjects and sleep-disordered patients. U-Sleep requires only a single EEG and a single EOG channel with arbitrary standard electrode placement as input, makes no assumptions about the acquisition hardware (including sampling rate) or pre-processing pipeline, and outputs a whole night’s sleep stages in seconds on a laptop CPU. U-Sleep also has a unique in-built ability to output sleep stage labels at temporal frequencies up to the signal sampling rate^43^. We show that such high-frequency representation of sleep carries diagnostic information in separating obstructive sleep apnea (OSA) patients from a population of healthy control subjects.

**Fig. 1:**
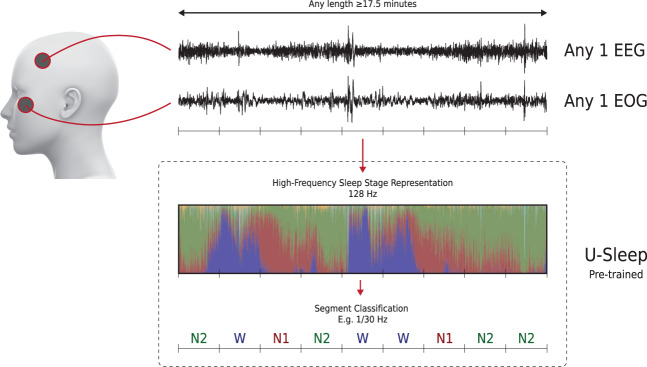
The U-Sleep prediction pipeline. U-Sleep is a ready-to-use deep neural network for sleep staging. First, it maps each provided EEG and EOG channel pair (shown in the top) to an intermediate, high-frequency sleep stage representation (shown in the middle). The intermediate representation is visualized by the colored bars indicating the level of confidence U-Sleep has that the subject is in one of the 5 sleep stages at a given time (Blue: Wake, Red: N1, Green: N2, Cyan: N3, Yellow: REM). From the intermediate representation, U-Sleep aggregates confidence scores over periods of time (for instance segments of 30 s) to output final sleep staging scores. U-Sleep makes no assumptions about the PSG protocol including acquisition hardware, electrode positions, filtering, and sampling rate. Internally, signals are re-sampled at 128 Hz. U-Sleep may output sleep stage labels up to this frequency.

**Fig. 2:**
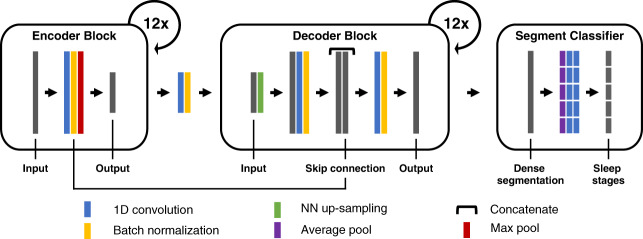
Model architecture. U-Sleep is a special fully convolutional neural network architecture designed for physiological time-series segmentation tasks such as sleep staging. It consists of an encoder (left) which encodes the input signals into dense feature representations, a decoder (middle) which projects the learned features into the input space to generate a dense sleep stage representation as shown in Fig. 1, and finally a specially designed segment classifier (right) which generates sleep stages at a chosen temporal resolution. Please see the Method section and Supplementary Table 2 for details on the U-Sleep model architecture.

Figure 1 provides an overview of the U-Sleep prediction pipeline. Figure 2 illustrates the model architecture. U-Sleep is freely available at https://sleep.ai.ku.dk.

## Results

### Datasets and model training

We trained and evaluated U-Sleep on 19,924 PSG records collected from 15,660 participants of 16 independent clinical studies (21 datasets). A brief overview of each dataset along with key demographic statistics are displayed in Table 1, details can be found in Supplementary Note: Datasets. All datasets are publicly available, some require an approval. The datasets can be split into two groups. First, there are 13 datasets that were partly used to train the U-Sleep model. In combination they span ≈19.4 years of annotated signals. Each dataset was split into training (at least 75%), validation (up to 10%, at most 50 subjects) and testing (up to 15%, at most 100 subjects) subsets on a per-subject or per-family basis. All records from subjects in the training sets were used to train the U-Sleep model. Records in the validation sets where used to monitor the performance of U-Sleep throughout training. Records in the testing subsets were used for evaluation.

**Table 1:** Datasets overview.

Type	Dataset	Public	Records	Subjects	Length (days)	Age (years)	BMI	Sex % (F/M)
Internal - Train/Test	ABC	(✓)	132	49	46.2	48.8 ± 9.8	38.9 ± 2.9	43/57
	CCSHS	(✓)	515	515	240.1	17.7 ± 0.4	25.1 ± 5.9	50/50
	CFS	(✓)	730	730/144^a^	300.8	41.7 ± 20.0^b^	32.4 ± 9.5	55/45
	CHAT	(✓)	1638	1232	679.6	6.6 ± 1.4	19.0 ± 4.9	52/48
	DCSM	✓	255	255	201.0	–	–	–
	HPAP	(✓)	238	238	77.6	46.5 ± 11.9	37.3 ± 9.2	43/57
	MESA	(✓)	2056	2056	905.5	69.4 ± 9.1	–	54/46
	MROS	(✓)	3926	2903	1877.3	76.4 ± 5.5	27.2 ± 3.8	0/100
	PHYS	✓	994	994	309.8	55.2 ± 14.3	–	33/67
	SEDF-SC	✓	153	78	144.1	58.8 ± 22.0	–	53/47
	SEDF-ST	✓	44	22	14.8	40.2 ± 17.7	–	68/32
	SHHS	(✓)	8444	5797	3144.4	63.1 ± 11.2	28.2 ± 5.1	52/48
	SOF	(✓)	453	453	188.1	82.8 ± 3.1	27.7 ± 4.7	100/0
Hold-Out	ISRUC-SG1	✓	100	100	31.3	51.1 ± 15.9	–	44/56
	ISRUC-SG2	✓	16	8	4.9	46.9 ± 17.5	–	25/75
	ISRUC-SG3	✓	10	10	3.1	39.6 ± 9.6	–	10/90
	MASS-C1	(✓)	53	53	19.9	63.6 ± 5.3	–	36/64
	MASS-C3	(✓)	62	62	21.8	42.5 ± 18.9	–	55/45
	SVUH	✓	25	25	7.2	50.0 ± 9.4	31.6 ± 3.9	16/84
	DOD-H	✓	25	25	8.6	35.3 ± 7.5	23.8 ± 3.4	24/76
	DOD-O	✓	55	55	18.5	45.6 ± 16.5	29.6 ± 6.4	36/64

Please refer to the Supplementary Material for additional details on each dataset. Missing values are due to study design or anonymized data. Individual statistics may be computed over a smaller number of observations than the total number of subjects due to missing data. Datasets DOD-H and DOD-O are hold-out consensus scored datasets. (✓) = requires approval.

*ABC* Apnea, Bariatric surgery, and CPAP^61,62^, *CCSHS* Cleveland Children’s Sleep and Health Study^61,63^, *CFS* Cleveland Family Study^61,64^, *CHAT* Childhood Adenotonsillectomy Trial^61,65,66^, *HPAP* Home Positive Airway Pressure^61,67^, *MESA* Multi-Ethnic Study of Atherosclerosis^61,68^, *MROS* Osteoporotic Fractures in Men^61,69,70^, *SHHS* Sleep Heart Health Study^61,71^, *SOF* Study of Osteoporotic Fractures^61,72,73^, *PHYS* 2018 PhysioNet/CinC Challenge^74^, *SEDF* Sleep-EDF^75^, *SVUH* St. Vincent’s University Hospital/University College Dublin Sleep Apnea Database^76^, *DCSM* Danish Center for Sleep Medicine, *ISRUC* ISRUC-Sleep^77^, *MASS* The Montreal Archive of Sleep Studies^78^, *DOD* Dreem Open Datasets^31,79,80^.

^a^Number of distinct families.

^b^Assuming uniform age distribution in the binned data.

In the second group are eight datasets that were used for evaluation only, that is, no data from these sources were used in the model building process (neither for training nor hyperparameter selection). These datasets allowed an unbiased performance evaluation of U-Sleep when applied (unaltered) to new, clinical cohorts. Among others, we measured the performance of U-Sleep against human experts by considering held-out consensus-scored datasets produced by clinical experts. The performance of U-Sleep was compared to that of the individual experts evaluated against their consensus scores.

The combined training dataset spans a significant fraction of the expected clinical population including large numbers of healthy individuals, patients with sleep and non-sleep related disorders, men and women, as well as different age-, BMI- and ethnic groups. The datasets were collected across geographically diverse locations (although mainly from the US), across decades, and on a variety of hardware using different sampling rates, hardware filters and more. The datasets were scored by sleep experts with different backgrounds.

Our goal is to perform accurate sleep staging across all cohorts simultaneously. In contrast to most studies, we deliberately exposed the machine learning system to highly variable data and labels, in order to learn a final model which generalizes well and is useful in clinical practice where data may vary unexpectedly and with time. U-Sleep was trained on randomly selected batches sampled across the datasets as described in the Methods section. For each sample in a batch, U-Sleep was exposed to a randomly selected EEG and EOG channel combination picked from all possible combinations for the given PSG. No information was given to the model about the data sources. This challenging setup forced U-Sleep to become invariant to electrode placements. We designed U-Sleep to require only a single EEG and a single EOG channel, where the electrode placement does not matter as long as it is a standard position, to maximize its applicability and ease-of-use. We omitted other modalities such as EMG, which carry important information about sleep disturbances and disorders (e.g., Parkinson’s Disease and REM sleep behavior disorder), but are not necessary for the delineation of sleep stages. Adding EMG has the potential to further improve the performance of U-Sleep. However, EMG signals especially help to distinguish between being awake and REM sleep, two stages that our predecessor system U-Time already separates very well. In preliminary experiments, adding EMG did not improve the performance of U-Time (see supplementary Table S. 12 in the study by Perslev et al.^43^). Using only the two most common modalities makes our model widely applicable, in particular in scenarios without advanced sleep monitoring setups, and allowed us to combine many datasets for training, some of which did not, for example, contain EMG recordings.

### Performance overview

U-Sleep was able to learn sleep staging across all training datasets simultaneously. Supplementary Fig. 3 shows both the overall loss and mean F1 score computed across validation subsets for each individual training dataset. The U-Sleep performance increased at similar rates for all datasets.

We used the trained U-Sleep to predict the full hypnogram of all PSG records in the test subsets of all datasets using all available combinations of EEG and EOG channels. Given the large number of results, we focus on the mean and stage-wise F1/Dice metrics computed across subjects for each dataset as described in the Methods section. The per-channel evaluations are shown for each dataset in Supplementary Tables 4–25. Table 2 lists the F1 scores using majority vote, that is, the hypnograms were generated using predictions from all available EEG-EOG channel combinations within each record. Majority voting, as can be seen from the channel-wise results in the Supplementary Material, always performed at least as good as the average over all possible channel combinations. For 19 out of the 21 datasets, the majority voting performed at least as good as the best individual channel (see Supplementary Material).

**Table 2:** Majority vote results overview.

Type	Dataset	Records	Wake	N1	N2	N3	REM	Mean
Internal - Train/Test	ABC	20	0.87	0.53	0.84	0.72	0.90	0.77
	CCSHS	78	0.93	0.63	0.91	0.88	0.93	0.85
	CFS	92	0.93	0.52	0.89	0.84	0.91	0.82
	CHAT	128	0.93	0.64	0.87	0.90	0.90	0.85
	DCSM	39	0.97	0.48	0.86	0.83	0.89	0.81
	HPAP	36	0.91	0.48	0.84	0.78	0.90	0.78
	MESA	100	0.92	0.59	0.87	0.65	0.90	0.79
	MROS	134	0.93	0.46	0.87	0.68	0.88	0.77
	PHYS	100	0.84	0.60	0.84	0.81	0.87	0.79
	SEDF-SC	23	0.93	0.57	0.86	0.71	0.88	0.79
	SEDF-ST	8	0.80	0.58	0.88	0.64	0.91	0.76
	SHHS	140	0.93	0.51	0.87	0.76	0.92	0.80
	SOF	68	0.93	0.45	0.86	0.77	0.92	0.78
Hold-Out	ISRUC-SG1	100	0.89	0.52	0.79	0.77	0.88	0.77
	ISRUC-SG2	16	0.85	0.49	0.78	0.83	0.86	0.76
	ISRUC-SG3	10	0.90	0.55	0.78	0.74	0.85	0.77
	MASS-C1	53	0.94	0.41	0.81	0.61	0.88	0.73
	MASS-C3	62	0.93	0.54	0.87	0.75	0.91	0.80
	SVUH	25	0.80	0.37	0.81	0.78	0.88	0.73
	DOD-H	25	0.91	0.60	0.87	0.79	0.94	0.82
	DOD-O	55	0.90	0.52	0.86	0.74	0.92	0.79
		Mean (weighted)	0.91	0.53	0.86	0.77	0.90	0.79
		STD (weighted)	0.03	0.07	0.03	0.08	0.02	0.03

For each record in each dataset, U-Sleep generated a hynogram using all possible combinations of 1 EEG and 1 EOG channel. Results reported here are from the majority voted hypnograms across all such combinations as described in the Methods section. We refer to Supplementary Tables 4–25 for per-channel results. Here we report the global F1 scores across all subjects in each dataset. The reported Mean (weighted) and STD (weighted) statistics are computed across datasets in each column weighted by the number of PSG records in each row.

Across 21 datasets, U-Sleep performed sleep staging with mean F1 ± STD (in parenthesis shown when weighted by number of test records) of 0.90 ± 0.04 (0.91 ± 0.03) for stage Wake, 0.53 ± 0.07 (0.53 ± 0.07) for stage N1, 0.85 ± 0.04 (0.86 ± 0.03) for N2, 0.76 ± 0.07 (0.77 ± 0.08) for stage N3, and 0.90 ± 0.02 (0.90 ± 0.02) for stage REM. Considering the mean computed across stages for each dataset, the global F1 performance can be summarized as 0.79 ± 0.03 (0.79 ± 0.03) ranging from a minimum 0.73 (SVUH) to maximum 0.85 (CCSHS and CHAT). The standard deviation over F1 scores obtained using each available channel combination was for most datasets below 0.02 (mean 0.01), with datasets MASS-C1 and ABC being the only exceptions with standard deviations of 0.03.

Examples of hypnograms as computed by U-Sleep using channel majority voting are visualized and compared to human expert annotations for all 21 testing datasets in in Supplementary Figs. 4–24. Specifically, we display the predicted hypnogram with the single highest F1-score, the single lowest F1-score and the one nearest to the median F1 score observed for the dataset. Thus, the figures visualize the span in U-Sleep performance from worst- to best-case scenario.

### Consensus results: comparing to human experts

**Fig. 3:**
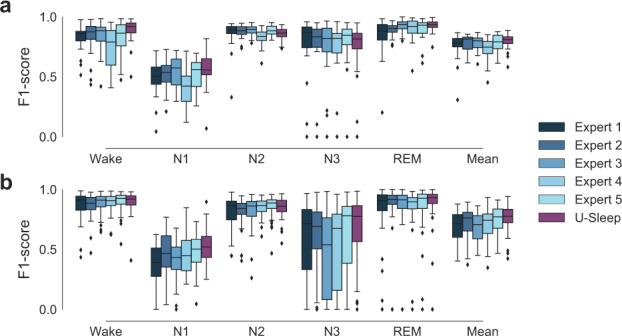
Boxplots illustrating the distributions of F1 scores from 5 human experts and U-Sleep on healthy controls and OSA patients. **a** Shows results from dataset DOD-H on 25 healthy subjects. **b** Shows results from dataset DOD-O on 55 patients suffering from OSA. Sleep stages produced by U-Sleep and the five individual experts were compared to consensus-scored hypnograms. Please refer to the Methods section for further details. Mean F1 scores averaged across stages are shown along with F1 scores for the five individual sleep/wake stages. The performance of U-Sleep is shown in red colors (right most boxplot in each group). The performance of each human expert is shown in shades of blue (4 left most boxplots in each group). Note that some records were scored by both human experts and U-Sleep with very low F1 scores (0 in some cases) on individual classes. This especially concerns stage N3 in dataset DOD-O and most often happens for rare classes. For instance, a patient severely affected by OSA rarely enters the N3 deep sleep stage, and the resulting low number of observed N3 stages makes even a few errors result in a large deviation in the F1 score. Each boxplot shows the median (middle vertical line), first and third quartiles (lower and upper box limits) and whiskers that extend to 1.5 times the IQR added or subtracted the third and first quartiles, respectively. Data outside of this range is marked as outliers indicated by diamond shaped points.

In Table 3 we report the performance of U-Sleep on the consensus-scored datasets DOD-H (3a) and DOD-O (3b) compared to the performance of five individual clinical experts from which the consensus scores were generated. The distributions of scores are shown for U-Sleep and the five experts in Fig. 3.

**Table 3:** Consensus score results on datasets (a) DOD-H and (b) DOD-O.

DOD-H: Healthy controls, *N* = 25							
Scorer	Fit	Wake	N1	N2	N3	REM	Mean
Expert 1	–	0.83 ± 0.11	0.49 ± 0.15	0.86 ± 0.12	**0.78** ± **0.24**	0.84 ± 0.16	0.76 ± 0.11
Expert 2	–	0.83 ± 0.14	0.52 ± 0.11	0.88 ± 0.05	**0.78** **±** **0.23**	0.89 ± 0.06	0.78 ± 0.07
Expert 3	–	0.84 ± 0.12	0.54 ± 0.13	0.88 ± 0.05	0.74 ± 0.25	**0.93** **±** **0.05**	**0.79** **±** **0.07**
Expert 4	–	0.73 ± 0.18	0.40 ± 0.15	0.83 ± 0.07	0.75 ± 0.22	0.90 ± 0.09	0.72 ± 0.11
Expert 5	–	0.83 ± 0.14	0.53 ± 0.12	**0.89** **±** **0.04**	0.76 ± 0.24	0.90 ± 0.09	0.78 ± 0.08
U-Sleep	✗	**0.88** **±** **0.10**	**0.56** **±** **0.14**	0.86 ± 0.05	0.73 ± 0.23	**0.93** **±** **0.05**	**0.79** **±** **0.06**
SimpleNet	✓	0.83 ± 0.13	0.57 ± 0.14	0.90 ± 0.04	0.80 ± 0.23	0.90 ± 0.09	0.80 ± 0.07
DeepSleepNet	✓	0.84 ± 0.10	0.56 ± 0.13	0.90 ± 0.05	0.79 ± 0.24	0.88 ± 0.10	0.79 ± 0.07
SeqSleepNet	✓	0.81 ± 0.18	0.54 ± 0.14	0.87 ± 0.08	0.73 ± 0.25	0.86 ± 0.12	0.76 ± 0.11

DOD-O: OSA patients, *N* = 55							
Scorer	Fit	Wake	N1	N2	N3	REM	Mean
Expert 1	–	0.87 ± 0.11	0.38 ± 0.15	0.82 ± 0.13	0.59 ± 0.31	0.81 ± 0.25	0.69 ± 0.12
Expert 2	–	0.87 ± 0.09	0.46 ± 0.17	0.82 ± 0.11	0.61 ± 0.29	0.86 ± 0.22	0.72 ± 0.12
Expert 3	–	0.88 ± 0.09	0.42 ± 0.16	0.83 ± 0.13	0.46 ± 0.33	0.85 ± 0.22	0.69 ± 0.11
Expert 4	–	0.89 ± 0.09	0.46 ± 0.15	0.84 ± 0.07	0.52 ± 0.33	0.83 ± 0.24	0.71 ± 0.12
Expert 5	–	**0.90** **±** **0.08**	0.48 ± 0.15	**0.86** **±** **0.08**	0.62 ± 0.33	0.85 ± 0.22	0.74 ± 0.11
U-Sleep	✗	0.89 ± 0.09	**0.53** **±** **0.14**	0.85 ± 0.08	**0.66** **±** **0.30**	**0.88** **±** **0.20**	**0.76** **±** **0.10**
SimpleNet	✓	0.89 ± 0.09	0.52 ± 0.16	0.88 ± 0.11	0.63 ± 0.35	0.85 ± 0.22	0.75 ± 0.11
DeepSleepNet	✓	0.86 ± 0.11	0.46 ± 0.17	0.87 ± 0.10	0.67 ± 0.30	0.84 ± 0.22	0.74 ± 0.12
SeqSleepNet	✓	0.84 ± 0.13	0.46 ± 0.20	0.86 ± 0.10	0.59 ± 0.33	0.77 ± 0.28	0.71 ± 0.14

Highest scores from human experts and U-Sleep are highlighted in bold. Scores where one of the trained ML models (last three rows) performed as well or superior to U-Sleep are indicated by underlined numbers. However, these models were fit to the particular datasets, while U-Sleep has not seen any data from DOD-H and DOD-O during model building and training, indicated by checkmarks or crosses in the Fit column. Numbers shown are mean ± 1 standard deviation per-subject F1 scores computed between the output of a single model or human expert and the consensus scores generated from the 4 (N − 1) remaining (when comparing human to consensus) or best human annotators (when comparing model to consensus).

Across the 25 healthy subjects of DOD-H, U-Sleep matched the best performing human expert with a mean F1 score of 0.79 ± 0.07 and human expert scores ranging from a minimum 0.72 ± 0.11 (Expert 4) to a maximum 0.79 ± 0.07 (Expert 3). There is no significant difference between the performance of U-Sleep and the best human expert (Expert 3) at confidence level *α* = 0.05 (*W* = 150.0, *p* = 0.737, two-sided Wilcoxon test). U-Sleep scored higher mean F1 than all humans on stages Wake and N1, similar to the best individual expert (Expert 3) on stage REM and worse than all human experts on stage N3. U-Sleep performed on average on par with the best two models *SimpleNet* and *DeepSleepNet* from the six models evaluated in the publication presenting the data ^31^ (3 best shown here), which were trained on consensus-scored labels from the same data distribution.

Across the 55 OSA patients of DOD-O, U-Sleep had the highest mean performance of 0.76 ± 0.10 among the set of human experts and itself, with human performances ranging from a minimum 0.69 ± 0.12 (Expert 1) to a maximum 0.74 ± 0.11 (Expert 5). There is no significant difference between the performance of U-Sleep and the best human expert (Expert 5, *W* = 555.0, *p* = 0.072, two-sided Wilcoxon test). U-Sleep scored higher mean F1 than all humans on stages N1, N3 and REM, and slightly below Expert 5 on stages Wake and N2. U-Sleep performed as well or better than the reference models, which were trained on consensus labels.

### Evaluation of high frequency sleep stages

U-Sleep can output sleep stages at a higher frequency than that of the labels used during trained. We trained with a label frequency of 1/30 Hz—the most typical so called *page size* in sleep staging—but can provide sleep stage predictions at frequencies up to 128 Hz (input records may be sampled at a higher frequency, but will be re-sampled before analysis). Figure 1 visualizes these high-frequency scores. We argue that these scores can capture sleeping patterns on shorter time scales^43^. To show this, we performed a simple, but carefully designed study to investigate if there is predictive information in the high frequency scores. We describe the experimental details in the Methods section. We considered the datasets DOD-H and DOD-O. Our experiment evaluated the hypothesis that the healthy subjects and OSA patients are easier to discriminate by a classifier when extracting features from high-resolution sleep stage scores. We considered the output by U-Sleep at different frequencies and computed the occurrences of sleep-stage triplet transitions of the form (*s*_1_, *s*_2_, *s*_3_), where *s*_*x*_ ∈ {Wake, N1, N2, N3, REM} and *s*_1_ ≠ *s*_2_ and *s*_2_ ≠ *s*_3_. The extracted triplet frequency features are time-invariant. We get the same number of features independent of the frequency at which we computed them. We fitted Random Forrest^44^ classifiers to separate the healthy and OSA patients using features extracted at different frequencies.

**Fig. 4:**
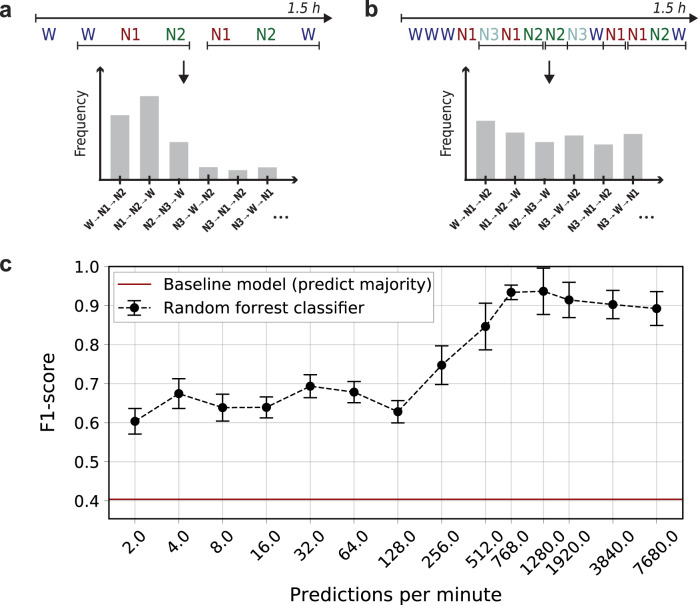
Classification performance on the task of separating healthy control subjects and OSA patients in a population of *N* = 80 (25 controls, 55 OSA patients) using a Random Forrest classifier on sleep stage transition triplet frequencies extracted using U-Sleep outputs of varying frequency. **a** and **b** Illustrate the process of extracting sleep stage triplet transition frequencies from low (**a**) and high (**b**) frequency outputs from U-Sleep, which are passed to the classifier. **c** Shows classification performance as a function of sleep staging frequency. Increasing the temporal resolution improved the predictive performance of the downstream classifier from its initial low mean F1 of 0.60 to nearly perfect classifications with mean F1 scores in range 0.89–0.94 at frequencies ≥768 predictions/minute. The black curve shows the mean performance with standard deviation error bars computed over 50 repetitions of the experiment using randomly configured classifiers. The solid red line is the F1 score obtained using a baseline model which predicts only the majority class (OSA patient) independent its input.

Figure 4 shows the result of the experiment. We evaluated the classification performance on sleep stages generated by U-Sleep at 14 different frequencies approximately uniformly distributed on a ${\mathrm{log}\,}_{2}$ scale from 2 stages/minute to 7680 stages/minute (128 Hz). The mean F1 classification performance increased from an initial low value of 0.60 (at 2 stages/minute frequency) up to a maximum of 0.94 (at 1280 stages/minute), indicating that the task of separating healthy and OSA patients was much easier using high-frequency scores, and, consequently, that such stages are indeed clinically informative.

## Discussion

U-Sleep has simultaneously learned sleep staging for a wide range of clinical cohorts without requiring adaptation to different cohorts. It can deal with large variations in patient demographics and PSG protocols, and only requires an arbitrary single EEG and EOG channel as inputs. We evaluated U-Sleep on several datasets that it has not seen during training, and we found that its accuracy matches the performance of models that were specifically developed and/or trained on these datasets. For instance, U-Sleep matches the performance of its predecessor U-Time (for a performance comparison of U-Time with other sleep staging approaches we refer to Perslev et al.^43^) trained specifically on datasets ISRUC-SG1 and SVUH^43^ with both models scoring 0.77 and 0.73 mean F1, respectively. U-Sleep also approximately matches the performance of U-Time on datasets SEDF-SC (U-Sleep: 0.79, U-Time: 0.76), PHYS (U-Sleep: 0.79, U-Time: 0.77) and DCSM (U-Sleep: 0.81, U-Time: 0.79). However, the scores of U-Time on these datasets span additional records, so the results cannot be compared directly. U-Sleep performs nearly as well as DeepSleepNet on MASS-C3^28^ (mean F1 of 0.82 for DeepSleepNet and 0.80 for U-Sleep). It is even as accurate as the best human expert of a group of five when evaluated on the datasets DOD-H and DOD-O with healthy and diseased individuals. It performs at least as well as all six automated systems evaluated in the original study presenting these data^31^. In contrast to U-Sleep, these six models were all trained on the same consensus-scored labels that define the ground truth, which gives them the advantage of learning from higher quality labels as well as a matching label distributions at training and test time.

In contrast to other automated systems, U-Sleep is trained to work with any standard EEG and EOG channels it receives as input. The measured F1 scores do vary between individual channels (as seen in Supplementary Tables 4–25), but with a low standard deviation for most datasets. Prediction by combining the available channels using majority vote almost always matches the prediction using the best individual channel. As majority scores can be easily obtained also in practice, this result relief sleep researchers from testing which channel combinations work best for their specific patients and data. In accordance with our clinical experience, we did not find specific EEG and EOG channel combinations that score particularly well or badly across datasets. It is possible that the performance scores obtained using a specific channel reflect what information the human annotators focused on when annotating the signals, as individual experts may have personal preference (or training) when detecting certain sleep stage characteristics such as spindles and K-complexes in a particular set of channels. As U-Sleep is trained on randomly varying channel combinations, it is forced to learn robust features that are conceivable across EEG and EOG channels. We hypothesize that U-Sleep utilizes its ability to look minutes into both the past and future to detect more global sleeping patterns that are observable across channels, but may be difficult to conceive for humans.

Developing sleep staging systems based on deep learning is an active research area, and new findings will further improve U-Sleep. When trained on single datasets, some recent algorithms may perform better than the general U-Sleep system^36,37^. While recent work showed that specialized systems can be applied to new datasets with good performance using transfer learning techniques^32,39^, these methods were retrained on new data matching the target cohort, which requires technical expertize, time, specialized hardware, and labelled data from the target domain. However, no system has demonstrated the robustness of U-Sleep on the much more difficult and relevant task of resilient sleep staging across new clinical cohorts, different input channels, etc. without additional training.

The U-Sleep architecture was designed based on our experience with U-Net-type neural networks^42,45–47^, please refer to the Methods section for details. It is a limitation of our study that, because of the long training time and in order to avoid problems due to adaptive data analysis, we have not fine-tuned the U-Sleep architecture and training procedure. It is possible that small modifications could further improve the results. Also, while we have attempted to compile as many and diverse datasets as possible (e.g., with respect to demographics), all datasets used so far were collected in either Europe or North America, and represent in particular healthy subjects and OSA patients; two groups both likely to display normal EEG patterns. It remains to be systematically studied how U-Sleep performs on patients with highly abnormal brain activity patterns (e.g., following stroke or due to psychiatric diseases or neurodegenerative disorders). In addition, we have only limited patient record information available for all subjects. Accordingly, it has not been possible to fully rule out all potential (e.g., regional) biases of U-Sleep. It is our hope that more sleep data will be made available from currently underrepresented groups of subjects, training on which will reduce the risk of unintentional biases. In Supplementary Note: Demographic Bias, we report the effects of age, sex and gender, finding increasing age to have a statistically significant (but small in magnitude) negative effect on performance, which we attribute to general decrease in health with age.

U-Sleep is an accurate, carefully evaluated and ready-to-use system for sleep staging. Therefore, we believe that the public availability of U-Sleep will benefit researchers and clinicians in sleep medicine. It can augment the workflow of expert clinicians by immediately providing sleep stage annotations of high quality when a PSG sample is inspected. While we do not advocate to disregard the invaluable expertize of the local clinical and technical staff, who will undoubtedly have a significantly better understanding and experience with patients and data from their clinic, we think that significant resources can be saved by using U-Sleep’s predictions as a starting point for sleep staging. In this case, the expert only needs to spot potential errors or disagreements with the system’s output instead of scoring the whole PSG manually. Furthermore, U-Sleep can provide highly accurate sleep staging when experienced experts are missing. It computes in seconds on a laptop CPU and requires no technical expertize to use, which makes it applicable for home-monitoring and sleep clinics in developing countries.

U-Sleep may facilitate large-scale, global studies of sleep with more consistent and less biased labels. While manual sleep staging follows guidelines as suggested by, for example, the AASM^16^, it is a difficult process with room for interpretation, making it inconsistent and error prone^19^. Different clinics may perform sleep staging slightly differently, which may introduce systematic biases when pooling data from clinical sites. While U-Sleep may make errors, these are more consistent. U-Sleep could thus be used to annotate large collections of data from across the world, facilitating the on-going and presumably important transition to large-scale sleep studies^48^. Individual clinics may be interested to compare their scores against those of U-Sleep, which may spark scientific debate about observed differences.

The ability of U-Sleep to output high-frequency sleep stages has the potential to significantly impact the study of sleeping patterns in health and disease, as demonstrated by our proof-of-concept experiments separating OSA patients and healthy controls. The current standard for sleep classification has developed only little since its first formulation in 1968^15^, in particular given the great progress made toward understanding sleep physiology. Sleep staging today almost always considers the brain as if it would move discretely from one stage to another over segments of exactly 30 s, failing to account for sleep dynamics on shorter time scales^9^. As we have shown, sleep stage scores at much higher frequency may serve as the basis for building future diagnostic predictive models. Such models—which may take additional input modalities such as EMG and demographic variables into account—may require significantly less training data compared to models that must learn to solve a predictive diagnostic task from raw PSG data alone, because they can utilize that U-Sleep has already digested the complex raw signals into an informative, high-frequency representation of sleep.

## Methods

### Fully convolutional neural network for time series segmentation

The U-Sleep model is a deep neural network, which maps an EEG and an EOG signal to a high-frequency sleep stage representation and then aggregates this intermediate representation to a sequence of sleep stages each spanning a fixed-length time interval (e.g., 30 s). This process is illustrated in Fig. 1. U-Sleep accepts input signals obtained with any common electrode placement (i.e., any EEG and EOG channel), hardware and software filtering, and sampling rate (internally re-sampled to 128 Hz). Up to computer memory constraints, U-Sleep processes inputs of arbitrary lengths. However, inputs shorter than 17.5 min may reduce performance by restricting the model from observing long-range dependencies in the data. It predicts sleep stages for the whole sequence in a single forward pass. This makes it possible for U-Sleep to process a whole night’s PSG data in seconds on commodity hardware and even in less than a second if a graphics processing unit (GPU) is used.

In contrast to other automated sleep staging systems, U-Sleep is a purely feed-forward, fully convolutional neural network. Fully convolutional networks have been incredibly successful in computational vision and especially in medical image analysis. They mark the state-of-the-art in image segmentation, with the *U-Net* arguably being the most popular architecture so far^40,41^. We successfully applied U-Nets for various medical segmentation tasks, and found that one fixed architecture and set of hyperparameters can give excellent results across very different tasks^45–47^. Recently, we adapted our version of *U-Net* for image analysis^42,45–47^ to the segmentation of one-dimensional physiological time series data. We extended the architecture with an additional block of fully convolutional layers for aggregating classifications^43^. The new architecture termed *U-Time* was applied to sleep staging.

In accordance with our results on images, we found that we could use the same network architecture and training process to learn a variety of sleep staging tasks outperforming state-of-the-art models such as DeepSleepNet^28^. Our fully convolutional network was easier to train (e.g., less dependent on hyperparameter settings) compared to more complex models for sleep staging relying on recurrent neural network architectures^43^. Another decisive feature of U-Time is that it provides a classification of the input signals for each time point as an intermediate representation, although the data used for training and evaluating the model were segmented at a much lower temporal resolution. The U-Sleep architecture proposed in this study supersedes U-Time; the main differences between the systems are described below.

### Automated sleep staging

Sleep staging refers to the process of partitioning a PSG record into a sequence of sleep stages. Human annotators typically consider segments of 30 s and assign a single sleep stage to each segment. We denote a PSG record by ${\bf{X}}\in {{\mathbb{R}}}^{\tau S\times C}$, where *τ* is a number of seconds sampled, *S* is the sampling rate and *C* is the number of channels recorded. The output of the sleep staging process is a sequence of ⌊*τ* ⋅ *e*⌋ labels, where *e* is the frequency at which we want to assign sleep stages, with *e* = 1/30 Hz being the typical value for human annotators. Thus, each sleep stage spans *i* = *S*/*e* sampled points in time across *C* channels.

Given a fixed integer *i* > 0, U-Sleep defines a deterministic function $f({\bf{X}}^{\prime} ;\theta ):{{\mathbb{R}}}^{T\cdot i\times C}\to {{\mathbb{R}}}^{T\times K}$ for any integer *T* > 0, where *θ* is a set of parameters learned from data, ${\bf{X}}^{\prime}$ is a (section of a) PSG record, *T* is a number of fixed-length segments with *i* sampled points each, *C* the number of PSG channels and *K* is the number of sleep stages. During training, ${\bf{X}}^{\prime}$ is typically a submatrix of a longer PSG **X** with ${\bf{X}}^{\prime} ={\bf{X}}[\{t,\ldots ,t+i\cdot T\},\{1,\ldots ,C\}]$ for some time point *t*. That is, U-Sleep takes a temporal section of a PSG and outputs a sequence of labels corresponding to fixed-length, contiguous segments of time (in principle, different output labels of U-Sleep could span different lengths of time, but we assume the typical case of fixed-length segments). The input ${\bf{X}}^{\prime}$ can be any length (augmented or cut to a multiple of *i*; ideally *T* ⋅ *i* ≥ 4096, because there are 12 pooling operations down-sampling the signal by a factor of 2 each). For instance, when we trained U-Sleep, ${\bf{X}}^{\prime}$ spanned 17.5 min of a PSG signal. When using U-Sleep to predict sleep stages in new data, the whole PSG is input to U-Sleep (i.e., ${\bf{X}}^{\prime} ={\bf{X}}$), which computes the whole hypnogram at once.

The provided U-Sleep system requires at least two input channels (*C* = 2), one EEG and one EOG channel, respectively, sampled or re-sampled to 128 Hz. It assumes *K* = 5 different stages {Wake, N1, N2, N3, REM}.

### Machine learning model

U-Sleep is a fully convolutional deep neural network refining its predecessor U-Time^43^, which we recently devised for time-series segmentation problems such as sleep staging (the differences between U-Sleep and U-Time are described below). In the following, we outline the U-Sleep architecture. We refer to Fig. 2 for a schematic overview and to Supplementary Table 2 for additional details on the configuration of the individual layers.

U-Sleep consists of three sub-modules: (1) An *encoder* module first extracts a deep stack of abstract feature maps from the input signals. Each extracted feature map has a lower temporal resolution compared to its input. (2) A *decoder* module then performs an up-scaling of the compact feature maps to match the temporal resolution of the input signals. The output of the decoder may be seen as a complex representation of sleep stages at a frequency matching the input signal. (3) A specially designed *segment classifier* module aggregates the intermediate, high-frequency output of the decoder into segments and predicts the sleep stages for these segments. For each segment, a confidence score is predicted for every possible sleep stage, which is interpreted as a probabilistic prediction by applying the softmax-function. Next, we describe the individual modules in more detail.

#### Encoder

The encoder module comprises 12 *encoder blocks*. Each encoder block consists of one convolutional layer (kernel size 9, no kernel dilation, stride 1^49^), one layer of Exponential Linear Unit (ELU)^50^ activation functions, batch normalization^51^ and max-pooling (kernel size 2, stride 2). The number of learned filters *c*_*l*_ in the *l*-th convolutional layer is $\sqrt{2}$ times larger compared to the previous layer, starting with *c*_1_ = 5, that is, for *l* ∈ {1, . . . , 11} we have ${c}_{l+1}=\lfloor {c}_{l}\sqrt{2}\rfloor$ (this corresponds to a doubling of the degrees of freedom from one block to the next, which is less than in U-Net implementations).

#### Decoder

The decoder module consists of 12 *decoder blocks*. Each decoder block performs nearest neighbour up-sampling of the input with kernel size 2 (i.e., it doubles the length of the feature maps along the temporal axis) and applies convolution (kernel size 2, stride 1), ELU activation functions and batch normalization. The up-scaled input is then combined with the output of the batch-norm operation (i.e., before max-pooling) of the corresponding encoder block (in terms of temporal resolutions, e.g., the first decoder block matches the last encoder block). Then a convolution, non-linearity, and batch-normalization are applied to the stacked feature maps. Opposite to the encoder, the decoder scales down the number of learned filters by a factor of $\sqrt{2}$ in each consecutive block.

The output of the final decoder has the same temporal resolution as the input signal. Thus, when concatenated, the encoder and decoder modules map an input signal in ${{\mathbb{R}}}^{T\cdot i\times C}$ to an output in ${{\mathbb{R}}}^{T\cdot i\times K}$, where *K* = 5 is the number of sleep stages. This output can be regarded as an intermediate representation of sleep stages at high (128 Hz) frequency.

#### Segment classifier

The segment classifier module maps the intermediate, high-frequency representation to the sleep stage prediction at the desired frequency. It aggregates scores over longer segments of time. For a given window of length *i* it first applies a per-channel mean-pooling operation with kernel width *i* and stride *i*. Two point-wise convolution operations (kernel width 1, stride 1) are then applied, the first using ELU activation functions. This allows to learn a non-linear weighted combination of the mean scores over the interval. Finally, the softmax-function is used to transform the scores into probabilistic predictions. Thus, the output of the segment classifier is a *T* × *K* right stochastic matrix, where *T* is a number of segments and *K* = 5 is the number of sleep stages. During training, we have one sleep stage label available for each segment of length *i*, and we train the whole encoder + decoder + segment classifier network end-to-end as described in the Optimization section below.

### Model specification and hyperparameter selection

The deep neural network architecture of U-Sleep is well-structured and simple in comparison to many others deep networks proposed for sleeep staging. Still, U-Sleep has many hyperparameters (e.g., the depth, the number of filters and their sizes for each block, etc.) which could be optimized to tune its performance on any specific set of data. However, we deliberately did not systematically tune the hyperparameters of U-Sleep, but employed a minimal hyperparameter selection strategy based on empirical evidence gathered from U-Time^43^, our experience from using fully convolutional neural networks for image segmentation^42,45,46^, and our physiological understanding of sleep staging. We avoided automated hyperparameter search to limit unintentional method-level overfitting and problems due to adaptive data analysis.

We adopted large parts of the U-Sleep model architecture (Supplementary Table 2) and hyperparameters (Supplementary Table 3) from its predecessor U-Time^43^, which was shown to be able to learn sleep staging across a range of datasets (individually) without requiring dataset-specific hyperparameter tuning. Still, we changed important aspects of the system. Because U-Sleep solves a significantly more difficult learning task requiring generalization across clinical cohorts and input channel combinations we increased the capacity of the network. The increased dataset size allowed us to fit a more complex model. In addition, we improved the system based on lessons learnt from U-Time. U-Sleep has a larger number of trainable parameters (≈3.1 ⋅ 10^6^ compared to U-Time’s ≈ 1.1 ⋅ 10^6^) and is significantly deeper, consisting of 12 encoder- and decoder blocks instead of U-Time’s four. U-Sleep also down-samples the input signal and subsequent feature maps much more slowly by using max-pooling kernels of width 2 in all encoder blocks instead of U-Time much more aggressive max-pooling kernels of widths in {10, 8, 4, 2}. U-Sleep implements the more complex ELU non-linearity following all convolution operations instead of U-Time’s Rectified Linear Units. Finally, whereas U-Time only linearly combined the mean-pooled activations in the final segment classifier layer, U-Sleep applies two convolution operations allowing for a non-linear weighted combination.

All changes served to increase the capacity of U-Sleep (i.e., its ability to approximate a more complex target function). Using a less aggressive max-pool down-sampling strategy reduces the information loss in the early layers. While U-Time benefited from early, aggressive down-sampling to reach computational and statistical efficiency, we argued that U-Sleep might need to capture more complex, hardly conceived patterns in the input signals which are robustly observed across datasets and channel combinations but may be lost if the input is sub-sampled too aggressively. The increased depth of U-Sleep also considerably expanded its theoretical receptive field^52^ (the maximum length of input signal that may effect each convolution computation in a given layer) from U-Time’s ≈ 5.5 min to ≈ 9.6 min in the last convolutional layer of the encoding sub-network. We numerically estimated the output sleep stages to be sensitive to changes in the input space 6.75 min backward and forward in time (i.e., each sleep stage prediction is informed by data from a window of up to 13.5 min of 128 Hz signal).

While U-Sleep has more layers compared to U-Time, the individual encoder- and decoder blocks are less complex, because they apply only a single convolution operation to their inputs instead of two, and the number of learned filters scale only by a factor of $\sqrt{2}$ with depth instead of 2 (see Supplementary Table 2).

Finally, we trained U-Sleep differently from U-Time to accommodate learning across many different datasets, and also apply augmentations as described in the Optimization and Augmentation sections below. The more common and simpler cross-entropy cost function was optimized instead of the generalized dice loss^53,54^ used for U-Time.

All reported results in this study are from the first and only trained instance of the U-Sleep model. That is, the design choices described above were not revised based on the performance of the system, making the reported evaluation metrics highly reliable.

### Pre-processing

All EEG and EOG signals are resampled to 128 Hz using polyphase filtering. We scale the range of EEG and EOG signals on a global, per-subject and per-channel basis so that the whole EEG signal recorded from a single channel has a median of 0 and inter quartile range (IQR) of 1 (i.e., an outlier robust scaling). We then clip any value which has an absolute deviation from the median of more than 20 times the IQR of that channel. Finally, during training we strip from the beginning and end any EEG or EOG signal which is outside the range of the scored hypnogram.

The current U-Sleep system considers sleep stages following the AASM standard: {W, N1, N2, N3, REM}^16^. If data was originally scored by a human expert following the Kales and Rechtschaffen^15^ manual, we merged stages S3 and S4 into a single N3. U-Sleep does not attempt to score stages such as ’MOVEMENT’ or ’UNKNOWN’. Whenever such a label occurred during training, we masked the loss contribution from that segment. This ensures that the model observes the segment in question, but its prediction does not influence the computation of the gradients for updating the model. We did not remove such segments entirely, as we want a model that can deal with such potentially noisy regions when scoring neighbouring segments after deployment.

### Optimization

For training of U-Sleep we used batches of size 64 sampled across the available training datasets. One element of a batch was a sequence of 35 segments of EEG and EOG data, each spanning 30 s, from a single subject. One label is specified for each such segment. That is, each batch element covered 17.5 min of signal and 35 labels. Batch elements were sampled from the training data using the following procedure:

1. **Dataset sampling:** We randomly select one of the available training datasets. The probability that a given dataset, *D*, is selected is given by *P*(*D*) = *αP*_1_(*D*) ⋅ (1 − *α*)*P*_2_(*D*), where *P*_1_(*D*) is the probability that a dataset is selected under discrete uniform sampling (i.e., all datasets are sampled with probability 1/*N* where *N* is the number of datasets) and *P*_2_(*D*) is the probability of sampling a dataset according to its size (number of PSG records in the dataset; i.e., a dataset of size 100 would be sampled 10 times more often than a dataset of size 10). Following the sampling policy of *P*_1_(*D*) means that all datasets (and thus clinical cohorts) are considered equally important in training independent of their size, while following *P*_2_(*D*) means that individual PSG records are equally important independent of the size of the dataset from which they originated. We set *α* = 0.5 to equally weigh *P*_1_(*D*) and *P*_2_(*D*), as we want the model to consider each individual sample while not (effectively) ignoring the smallest of training datasets.
2. **Subject sampling:** We uniformly sample a PSG record *S*_*D*_ from *D*.
3. **Channel sampling:** We uniformly sample 1 EEG and 1 EOG channel from those available for PSG record *S*_*D*_. For instance, if 2 EEG channels, C3-M2 & C4-M1, and 2 EOG channels, ROC-M1 & LOC-M2, are available, four combinations would be possible.
4. **Segment sampling:** We then select a segment of length *T* from the chosen EEG-EOG channel combination from PSG record *S*_*D*_. In our experiments, we set *T* = 35 (17.5 min). In order to counter class imbalance we select the temporal placement of the segment following these steps: (1) we uniformly sample a class from the label set {W, N1, N2, N3, REM}, (2) we then select a random sleep period of 30 s that the human annotator scored to be of the sampled class, (3) we shift the chosen sleep segment to a random position within the window of length *T*. This scheme ensures that even very rare sleep stages are visited. However, this approach does not fully balance the batches, as the *T* − 1 remaining segments of the input window are still subject to class imbalance, and some PSG records might not display a given minority class at all.

During training U-Sleep scored all 64 elements of a batch generating a total 2240 predicted sleep stages. In each step we updated the parameters of U-Sleep using the Adam optimizer^55^ with a learning rate *η* = 10^−7^ minimizing the standard and unweighted cross-entropy cost function. We continued training until 100 consecutive epochs of no validation loss improvement were observed. Due to the large training dataset size, we defined one epoch as 10^6^ sleep segments (or labels, equivalently) or 443 gradient steps. Note that we found applying regularization unnecessary when optimizing U-Sleep as overfitting was negligible, see Supplementary Fig. 3.

### Augmentation

Data augmentation refers to modifying the input data during training to improve generalization. We applied transformations to a random subset of the sampled batch elements, replacing variable lengths of segments within EEG and EOG channels or even entire channels with Gaussian noise. Specifically, for each sample in a batch, with probability 0.1, a fraction of the signals in that sample was replaced with noise from $N(\mu =\hat{\mu },{\sigma }^{2}=0.01)$, where $\hat{\mu }$ is the empirically measured mean of the sample’s signals. The fraction was sampled log-uniformly from [0.001, . . . , 0.3]. With probability 0.1 at most 1 channel was entirely replaced by noise. These augmentations were applied to force the model to consider both channels and complex distant relations in the signal.

### Input channel majority voting

When applying U-Sleep to new PSG data we utilize its ability to accept input data from arbitrary EEG and EOG electrode positions by predicting the full hypnogram for each combination of 1 EEG and 1 EOG channel possible for the given PSG. The resulting predictions are then combined to one final hypnogram. For each segment, the softmax scores (values ranging from 0 to 1 indicating the model’s confidence in each sleep stage) of all predictions are summed up and the sleep stage with the highest accumulated score is the final prediction for the segment.

The hypnogram based on an ensemble of predictions is likely to be more accurate than the individual hypnograms, as multiple predictions may smooth out errors if those are uncorrelated across channels^45,56^ and provide additional evidence to difficult, borderline cases.

### Evaluation

U-Sleep outputs sleep stages in {W, N1, N2, N3, REM} as described above. When evaluating U-Sleep we scored the full PSG, but did not consider the predicted class on a segment with a label different from the five sleep stages (e.g., a segment labelled ’MOVEMENT’ or, for whatever reason, not scored by a human expert at all). We predicted sleep stages using all combinations of available EEG and EOG channels for each PSG. Unless otherwise specified, we used majority voting fusing these predictions when evaluating U-Sleep. We refer to the supplementary material for channel-wise results.

We evaluated U-Sleep using the F1/Sørensen-Dice metric^57,58^, which is computed for each sleep stage *c* separately. The F1 score is defined as ${{\rm{F}}}_{\beta = 1}^{c}=\frac{2{\rm{TP}}}{2{\rm{TP}}+{\rm{FP}}+{\rm{FN}}}$, where TP, FP and FN are the number of true positives, false positives and false negatives for a given class *c*. The F1 score is used, because it emphasizes both recall and precision. We computed the F1 score for all five classes from (non-normalized) confusion matrices and report them separately or combined by calculating the unweighted mean. Note that unweighted F1 scores typically reduce the absolute scores due to lower performance on less abundant classes such as sleep stage N1.

Table 2 gives an overview over the results, reporting only F1 scores computed for a given class across all subjects of a testing set, which results in a single number without error bars. In Table 3 we consider F1 scores computed for each subject individually and report the mean and standard deviation, which may better reflect performance in a clinical setting.

Each PSG record in the datasets DOD-H and DOD-O was scored by five human experts. This allows us to compute consensus-scored hypnograms that may be regarded as ground truth data and then evaluate the performance of U-Sleep in relation to this ground truth as well as in comparison to individual human experts. We used the code provided with the DOD publication (see https://github.com/Dreem-Organization/dreem-learning-evaluation) for evaluating the consensus scores^31^, except that we did not balance the F1 scores measured for each class by the abundance of that class (we report unweighted mean F1 scores for consistency reasons). When comparing a human annotator to the consensus, the consensus hypnograms are generated from the *N* − 1 remaining expert scores. In accordance to the literature, U-Sleep and other automated methods reported in Table 3 were evaluated against consensus hypnograms based on the *N* − 1 most reliable annotators^31^.

### High-frequency sleep staging experiments

U-Sleep has the ability to make predictions at higher temporal resolutions compared to the the labels used during training. As an intermediate representation, U-Sleep computes a confidence score for each possible sleep stage at each sampled time point (i.e., at 128 Hz in the current system). An example of this is shown in Fig. 1. Sleep stages are inherently defined based on patterns observed over (longer) time periods. Thus, the question is whether the high-resolution outputs are informative of actual physiological sleeping patterns or only add more noise.

During training, our model considers the mean of the confidence scores over a 30 s segment, shuffling the scores within a segment would not change the learning and the prediction. Still, it is likely that the intermediate scores will reflect the true sleep stage at a time point, because only in that way the system can be independent of the—to a large extend arbitrary—positioning of the windows defining the segments.

One way to assess the usefulness of the scores is by linking them to a clinical diagnosis. We considered the datasets DOD-H and DOD-O (see Table 1 and the Supplementary Note: Datasets) with 25 healthy subjects and 55 OSA patients, respectively. As OSA patients suffered from abrupt awakenings and rapid transitions from deep sleep into lighter sleep stages, we expected a classifier to be able to separate the two populations with better-than-random performance given simple features describing the number of such transitions per time. For each subject in DOD-H and DOD-O, we predicted sleep stages at frequencies in {2, 4, 8, 16, 32, 64, 128, 256, 512, 768, 1280, 1920, 3840, 7680} predictions/min. We used all available combinations of EEG and EOG channels (16 for DOD-O and 24 for DOD-H) and computed the majority vote for each segment. For each subject we considered the two predictions/minute output for determining the onset and end of sleep (indicated by first and last sleep stage). For all frequencies, only the sleep stages within this time-frame were considered.

For each segment of 1.5 h of sleep we counted the number of occurrences of sleep stage transition triplets. A triplet is a sequence (*s*_1_, *s*_2_, *s*_3_) ∈ {W, N1, N2, N3, REM}^3^. We considered only triplets for which *s*_1_ ≠ *s*_2_ and *s*_2_ ≠ *s*_3_. This leaves 80 different triplets in which a fast transition to stage *s*_2_ occur (e.g., (N3, W, N1)) ignoring more typical triplets such as (N2, N2, N2).

We fit a random forest classifier^44^ (using the sklearn implementation^59^) to the triplet frequencies (occurrences per time). We fit the classifier to 79 out of the 80 subjects and predicted whether the last subject suffers from OSA or not, repeating the process for all subjects (leave-one-out cross validation). We repeated the whole experiment 50 times for each frequency bin with a small randomization in the hyperparameters of the random forest classifier. The latter is done to increase our confidence that any observed correlation is not related to a very specific set of hyperparameters. Specifically, in each repetition of the experiment we trained a random forests with 200 trees with respect to the Gini impurity measure and class weights *w*_*c*_ = *n*/(*k* ⋅ *n*_*c*_), where *w*_*c*_ is the weight associated with class *c*, *n* is the total number of samples, *n*_*c*_ the number of samples of class *c*, and *k* = 2 is the number of classes (’balanced’ mode in sklearn notation). A random value was chosen for the following hyperparameters: maximum_tree_depth ∈ {2, …, 7}, min_samples_leaf ∈ {2, . . . , 7}, min_samples_split ∈ {2, . . . , 7} and max_features ∈ {sqrt, log2}. We refer to https://scikit-learn.org/stable/modules/ensemble.html#forest for a detailed description of those parameters.

We determined the overall OSA classification for each subject by the majority vote over the predictions of the model across all segments of 1.5 h of sleep, ties were broken at random.

### Ethical approval

The Research Ethics Committee for SCIENCE and HEALTH, University of Copenhagen, has reviewed this research project and has found it compliant with the relevant Danish and International standards and guidelines for research ethics. The DCSM dataset was extracted and anonymized by the Danish Center for Sleep Medicine under a general approval from the Danish Data Protection Agency to analyze historical PSG data. All other datasets were acquired from third-party databases and handled according to the relevant data sharing agreements.

### Reporting summary

Further information on research design is available in the Nature Research Reporting Summary linked to this article.

## Supplementary information

Reporting Summary

Supplementary Materials

## Data Availability

We make the DCSM dataset publicaly available at 10.17894/ucph.282d3c1e-9b98-4c1e-886e-704afdfa9179. All other datasets are in principle also publicly available assuming the individual researcher and use-case is eligible for a given dataset as determined by the third-party dataset licence holders listed for each dataset individually in the Supplementary Material. Please refer to Table 1 for an overview of which datasets require approval and which are directly available. Confusion matrices for U-Sleep predictions on all channel combinations (including majority votes) for individual subjects in all test datasets may be downloaded from 10.17894/ucph.0d1554e9-d86b-4e08-b3c2-632b730cd362. These matrices allow re-computation of F1 metrics as reported here, as well as other metrics of interest. The repository also stores hyperparameter configuration files as well as dataset preprocessing and splitting information needed to reproduce the training of U-Sleep.

## Code availability

The in-house developed codebase used for training U-Sleep is publicaly available on GitHub at https://github.com/perslev/U-Time. The software includes a command-line-interface for initializing, training and evaluating models without the need to alter the underlying codebase. The software is based on TensorFlow^60^. Please refer to the README file of the repository for guidance on installation and a step-by-step guide on how to train a U-Sleep model on a subset or all of the datasets considered here. We trained U-Sleep on a single GPU (NVIDIA Titan X) with 12 GiB of memory. Because U-Sleep can score a full PSG in a single forward pass, segmenting 10+ h of signal takes only seconds on a laptop CPU and is practically instantaneous if running on a GPU. We make inference using the pre-trained U-Sleep model freely available at https://sleep.ai.ku.dk for non-commercial usage. Users may upload (anonymised or public domain) PSG files (European Data Format, EDF, or HDF5) to the service, choose parameters such as which channels to use and the inference frequency (e.g., 1/30 Hz or higher), and receive back the automatically scored hypnogram. The service also provides a simple interface to interactively visualize the scored hypnogram and to obtain key sleep statistics over selected periods of time. The raw sleep stages can be downloaded in several formats. We welcome community feedback on how we may improve the service with additional features.
