# Supplementary material for: U-Sleep: resilient high-frequency sleep staging
Source: NPJ Digit Med. 2021 Apr 15;4:72. doi: 10.1038/s41746-021-00440-5 (PMC8050216; doi:10.1038/s41746-021-00440-5)
Supplement: Supplementary file 1 — Reporting Summary [file 41746_2021_440_MOESM1_ESM.pdf]

nature research

Corresponding author(s): Christian Igel

Last updated by author(s): Oct 23, 2020

Reporting Summary

Nature Research wishes to improve the reproducibility of the work that we publish. This form provides structure for consistency and transparency in reporting. For further information on Nature Research policies, see our Editorial Policies and the Editorial Policy Checklist.

Statistics

For all statistical analyses, confirm that the following items are present in the figure legend, table legend, main text, or Methods section.

n/a	Confirmed	
☐	☒	The exact sample size (*n*) for each experimental group/condition, given as a discrete number and unit of measurement
☐	☒	A statement on whether measurements were taken from distinct samples or whether the same sample was measured repeatedly
☐	☒	The statistical test(s) used AND whether they are one- or two-sided<br>*Only common tests should be described solely by name; describe more complex techniques in the Methods section.*
☐	☒	A description of all covariates tested
☐	☒	A description of any assumptions or corrections, such as tests of normality and adjustment for multiple comparisons
☐	☒	A full description of the statistical parameters including central tendency (e.g. means) or other basic estimates (e.g. regression coefficient) AND variation (e.g. standard deviation) or associated estimates of uncertainty (e.g. confidence intervals)
☐	☒	For null hypothesis testing, the test statistic (e.g. *F*, *t*, *r*) with confidence intervals, effect sizes, degrees of freedom and *P* value noted<br>*Give P values as exact values whenever suitable.*
☒	☐	For Bayesian analysis, information on the choice of priors and Markov chain Monte Carlo settings
☒	☐	For hierarchical and complex designs, identification of the appropriate level for tests and full reporting of outcomes
☒	☐	Estimates of effect sizes (e.g. Cohen's *d*, Pearson's *r*), indicating how they were calculated

Our web collection on statistics for biologists contains articles on many of the points above.

Software and code

Policy information about availability of computer code

Data collection	All data considered in this manuscript were collected from publicly available (some requiring approval, please refer to the Data Availability statement) sleep resources such as PhysioNet (https://physionet.org) and the National Sleep Research Resource (https://sleepdata.org). Data from the later was downloaded using the NSRR Ruby Gem (https://github.com/nsrr/nsrr-gem) version 5.0.0.
Data analysis	Data was processed and used to train the U-Sleep model using our own custom built software package. The software package is provided with the manuscript for review. We will make the software publicly available on GitHub following the review period. Please refer to the README file for guidance on installation and usage. The public U-Sleep webservice may be accessed and reviewed at https://sleep.ai.ku.dk.

For manuscripts utilizing custom algorithms or software that are central to the research but not yet described in published literature, software must be made available to editors and reviewers. We strongly encourage code deposition in a community repository (e.g. GitHub). See the Nature Research guidelines for submitting code & software for further information.

Data

Policy information about availability of data

All manuscripts must include a data availability statement. This statement should provide the following information, where applicable:

- Accession codes, unique identifiers, or web links for publicly available datasets
- A list of figures that have associated raw data
- A description of any restrictions on data availability

All datasets used in this study are either publicly available or available upon reasonable request as determined by each third party license holder. Persistent links, references and additional details are provided in the supplementary material of the manuscript. U-Sleep classification scores, hyperparameter configuration files as well as dataset preprocessing and splitting information needed to fully reproduce the training of U-Sleep are available at https://sid.erda.dk/wsgi-bin/ls.py?share_id=HE5nA4Xs37 as well as described in the README file of the source code repository.

Field-specific reporting

Please select the one below that is the best fit for your research. If you are not sure, read the appropriate sections before making your selection.

☒ Life sciences ☐ Behavioural & social sciences ☐ Ecological, evolutionary & environmental sciences

For a reference copy of the document with all sections, see nature.com/documents/nr-reporting-summary-flat.pdf

Life sciences study design

All studies must disclose on these points even when the disclosure is negative.

Sample size

We collected as many and diverse polysomnography datasets as possible from all applicable public sleep data repositories as well as repositories for which we were able to apply and gain access for the purposes of this study. In total, we collected data from 15,660 study participants across 16 clinical studies. The combined dataset spans both large numbers of healthy individuals, patients with sleep and non-sleep related disorders, men and women, as well as different age-, BMI- and ethnic groups. The goal was to collect a dataset large enough to both facilitate the training of a resilient and accurate model for sleep staging and evaluate this model on several held-out clinical populations.

Data exclusions

Polysomnography records were excluded if either 1) they did not contain at least 1 EEG and at least 1 EOG channel (required by the U-Sleep model), or 2) no valid hypnogram annotation file was available for the study.

Replication

We evaluated our model on several datasets not used for model building.

Randomization

Each of the 16 clinical datasets considered were used either to train the sleep staging model or to evaluate it on data not used for model building. Each dataset was placed into one of these two groups ensuring 1) a large and highly variable training dataset, 2) significant variability in the held-out testing datasets (e.g. both healthy subjects and sleep-disordered individuals should be represented to maximize clinical significance in the evaluations) and 3) that our model could be directly compared to relevant competing methods as well as human expert annotators on data it was not trained on.

Each dataset assigned to the model building group was further randomly split into three sub-sets (training, validation and testing), as detailed in the manuscript. For this randomized assignment we ensured that multiple records from a single subject, or multiple subjects sharing family relations, were all assigned into the same sub-set in order not to positively bias our results.

Blinding

Blinding was not applicable as per the design of this study.

Reporting for specific materials, systems and methods

We require information from authors about some types of materials, experimental systems and methods used in many studies. Here, indicate whether each material, system or method listed is relevant to your study. If you are not sure if a list item applies to your research, read the appropriate section before selecting a response.

Materials & experimental systems

n/a	Involved in the study	
☒	☐	Antibodies
☒	☐	Eukaryotic cell lines
☒	☐	Palaeontology and archaeology
☒	☐	Animals and other organisms
☒	☐	Human research participants
☒	☐	Clinical data
☒	☐	Dual use research of concern

Methods

n/a	Involved in the study	
☒	☐	ChIP-seq
☒	☐	Flow cytometry
☒	☐	MRI-based neuroimaging
