# Supplementary material for: U-Sleep: resilient high-frequency sleep staging
Source: NPJ Digit Med. 2021 Apr 15;4:72. doi: 10.1038/s41746-021-00440-5 (PMC8050216; doi:10.1038/s41746-021-00440-5)
Supplement: Supplementary file 2 — Supplementary Materials [file 41746_2021_440_MOESM2_ESM.pdf]

Supplementary Material

Supplementary Notes **3**
Datasets . . . 3
Demographic Bias . . . 6

Supplementary References **56**

List of Figures

1 Demographic Bias . . . 7
2 Polysomnography Example . . . 8
3 U-Sleep Learning Curves . . . 9
4 Hypnogram Examples: ABC . . . 10
5 Hypnogram Examples: CCSHS . . . 11
6 Hypnogram Examples: CFS . . . 12
7 Hypnogram Examples: CHAT . . . 13
8 Hypnogram Examples: DCSM . . . 14
9 Hypnogram Examples: HPAP . . . 15
10 Hypnogram Examples: MESA . . . 16
11 Hypnogram Examples: MROS . . . 17
12 Hypnogram Examples: PHYS . . . 18
13 Hypnogram Examples: SEDF-SC . . . 19
14 Hypnogram Examples: SEDF-ST . . . 20
15 Hypnogram Examples: SHHS . . . 21
16 Hypnogram Examples: SOF . . . 22
17 Hypnogram Examples: ISRUC-SG1 . . . 23
18 Hypnogram Examples: ISRUC-SG2 . . . 24
19 Hypnogram Examples: ISRUC-SG3 . . . 25
20 Hypnogram Examples: MASS-C1 . . . 26
21 Hypnogram Examples: MASS-C3 . . . 27
22 Hypnogram Examples: SVUH . . . 28
23 Hypnogram Examples: DOD-H . . . 29
24 Hypnogram Examples: DOD-O . . . 30

List of Tables

1 Typical Sleep Stage Features . . . 31
2 U-Sleep Model Topology . . . 32
3 U-Sleep Hyperparameters . . . 33
4 Channel-Wise Results: ABC . . . 34
5 Channel-Wise Results: CCSHS . . . 35

6 Channel-Wise Results: CFS . . . 36
7 Channel-Wise Results: CHAT . . . 37
8 Channel-Wise Results: DCSM . . . 38
9 Channel-Wise Results: HPAP . . . 39
10 Channel-Wise Results: MESA . . . 40
11 Channel-Wise Results: MROS . . . 41
12 Channel-Wise Results: PHYS . . . 42
13 Channel-Wise Results: SEDF-SC . . . 43
14 Channel-Wise Results: SEDF-ST . . . 44
15 Channel-Wise Results: SHHS . . . 45
16 Channel-Wise Results: SOF . . . 46
17 Channel-Wise Results: ISRUC-SG1 . . . 47
18 Channel-Wise Results: ISRUC-SG2 . . . 48
19 Channel-Wise Results: ISRUC-SG3 . . . 49
20 Channel-Wise Results: MASS-C1 (part 1/2) . . . 50
21 Channel-Wise Results: MASS-C1 (part 2/2) . . . 51
22 Channel-Wise Results: MASS-C3 . . . 52
23 Channel-Wise Results: SVUH . . . 53
24 Channel-Wise Results: DOD-H . . . 54
25 Channel-Wise Results: DOD-O . . . 55

Supplementary Notes

Datasets

In the following, we briefly describe the datasets considered in this study.

ABC The Apnea, Bariatric surgery, and CPAP (ABC) study consists of PSG recordings from patients with severe obstructive sleep apnea (OSA) and morbid obesity (BMI of 35-45)[1,2]. The study addressed the effect of bariatric (weight loss) surgery in comparison to continuous positive airway pressure (CPAP) therapy for the treatment of OSA. The study pooled data from two different US sleep programs and spans a demographically diverse group of OSA patients. 53 subjects were enrolled in the original study, of which 49 were available to us for our work. EEG and EOG signals were recorded at 256 Hz and hardware low-pass filtered at 105 Hz and high-pass filtered at 0.16 Hz. Hypnograms were scored according to the AASM criteria. For more information, we refer to `https://doi.org/10.25822/nx52-bc11` and `https://clinicaltrials.gov/ct2/show/NCT01187771`.

CCSHS The Cleveland Children's Sleep and Health Study (CCSHS) is a large cohort of children and adolescents originally studied at ages 8-11[1,3]. The cohort is a stratified random sample of full-term and pre-term children born at 3 different hospitals around Cleveland, Ohio, US between 1988 and 1993. We considered PSG data as recorded in-lab during the third and final longitudinal visit which took place between 2006 and 2010. In our study, we had access to 515 samples of adolescents aged 16-19. EEG and EOG signals were recorded at 128 Hz and hardware high-pass filtered at 0.15 Hz. Hypnograms were scored according to the AASM criteria. For more information, we refer to `https://doi.org/10.25822/cg2n-4y91`.

CFS The Cleveland Family Study (CFS) is a large, family-based study of sleep apnea consiting of 2284 subjects from 361 families studied longitudinally between 1990 and 2006[1,4]. We considered data from the last visit (2006) at which full overnight PSG were measured. 730 subjects from 144 families participated in this study. When splitting data from `CFS` into train/test splits, we ensured that all family members appear in the same split. EEG and EOG signals were recorded at 128 Hz and hardware low-pass filtered at 105 Hz and high-pass filtered at 0.16 Hz. Hypnograms were scored according to the AASM criteria. For more information on this dataset, we refer to `https://doi.org/10.25822/jmyx-mz90`.

CHAT The Childhood Adenotonsillectomy Trial (CHAT) studied the effect of adenotonsillectomy surgery (removal of tonsils and adenoids) on mild to moderate obstructive sleep apnea (OSA) in children ages 5-10 years[1,5,6]. Subjects were assessed with full PSG at baseline and after a 7-month period. Study participants were recruited from 6 US sleep centres in Massachusetts, Missouri, New York, Ohio and Pennsylvania. We considered a total of 1638 PSG records from 1232 subjects (452 baseline, 407 follow-up, 779 control). Record `chat-baseline-300927` was excluded due to missing EOG channels. EEG and EOG signals were recorded at 200 Hz or higher (varies between studies) with variable hardware filtering applied depending on the acquisition system. Hypnograms were scored according to the AASM criteria. For more information, we refer to `https://doi.org/10.25822/d68d-8g03` and `https://clinicaltrials.gov/ct2/show/NCT00560859`.

HPAP The Home Positive Airway Pressure (HomePAP, abbreviated HPAP in this study) was a multi-site study with patients enrolled from 7 different US academic sleep centres to study the effectiveness of home-based portable monitoring as compared to laboratory-based PSG for the diagnosis and treatment of obstructive sleep apnea (OSA) in adults at least 18 years of age[1,7]. The study included 373 subjects of which we consider only the 247 who underwent lab-based PSG recordings. We excluded 9 subjects (IDs `1600052`, `1600138`, `1600280`, `1600047`, `1600194`, `1600361`, `1600087`, `1600368`, and `1600203`) due to missing EOG and/or reference channels. EEG and EOG signals were recorded at 200 Hz with no hardware filtering applied. Hypnograms were scored according to the AASM criteria. For more information on this dataset, we refer to `https://doi.org/10.25822/xmwv-yz91` and `https://clinicaltrials.gov/ct2/show/NCT00642486`.

MESA The *Multi-Ethnic Study of Atherosclerosis* (MESA) was a multi-ethnic longitudinal study of factors associated with the progression of cardiovascular disease across a cohort of black, white, Hispanic, and Chinese-American men and women aged 45-84 at study onset in 2000–2002[1,8]. Between 2010–2012, 2237 participants further enrolled in the *MESA Sleep* sub-study and underwent (among others) overnight unattended PSG. We had 2056 subjects available for our study. EEG and EOG signals were recorded at 256 Hz and hardware low-pass filtered at 100 Hz. Hypnograms were scored according to the AASM criteria. For more information, we refer to `https://doi.org/10.25822/n7hq-c406`.

MROS A sub-study of the larger *Osteoporotic Fractures in Men* (MrOS) study investigated the association between sleep patterns, sleep-disordered breathing and cognition in community-dwelling men aged 67 and above who were

not selected on the basis of sleep disorders or cognitive impairment[1,9,10]. Subjects were enrolled from 6 different US clinical sites in Alabama, Minnesota, Pennsylvania, Oregon, and California. Between 2003-2005, 3135 subjects enrolled of which 2909 underwent in-home overnight polysomnography (PSG). In this study, we considered a total of 3926 PSG records (2900 from visit 1 and 1026 from visit 2) from 2903 subjects. We excluded a total of 7 records (IDs aa2180, aa3370, aa1367, aa1715, aa1900, aa3903, aa3411 all from visit 1) due to missing EOG channels and/or sleep stage annotation files. EEG and EOG signals were recorded at 256 Hz and hardware high-pass filtered at 0.15 Hz. Hypnograms were scored according to the AASM criteria. For more information on the MROS dataset and studies, please refer to https://doi.org/10.25822/kc27-0425.

PHYS The over-night PSG data the from *2018 PhysioNet/CinC Challenge* were contributed by the Massachusetts General Hospital's Computational Clinical Neurophysiology Laboratory and the Clinical Data Animation Laboratory. The full dataset spans 1,985 patients who were monitored for the diagnosis of sleep disorders. The original challenge was automatic detection of arousal, but sleep stages were annotated by clinical staff. The dataset was split into two equal sized halves for training and testing. In our study we considered the 994 subjects publicly available in the training subset. EEG and EOG signals were recorded at 200 Hz. Hypnograms were scored according to the AASM criteria by a total of 7 annotators (1 scoring per PSG). For more information, we refer to https://physionet.org/content/challenge-2018 and[11,12].

SEDF-SC & SEDF-ST The *Sleep-EDF Database (Expanded)* consists of 197 whole-night PSG recordings. In the Sleep Cassette (SEDF-SC) sub-study, 153 PSGs were collected between 1987–1991 from healthy Caucasians aged 25–101 not taking sleep-related medication. The Sleep Telemetry (SEDF-ST) sub-study investigated the effect of temazepam intake on sleep in 22 Caucassian males and females. Participants took no other medication and were generally healthy but having mild difficulties falling asleep. Two recordings were collected from each individual on two nights at the hospital, one after temazepam intake and the other one after placebo intake. EEG and EOG signals were recorded at 100 Hz. Hypnograms were scored according to the Rechtschaffen and Kales criteria, which we aligned to AASM as described in the Methods section. The SEDF-SC database has been regularly used for benchmarking of automatic sleep stage classification algorithms. For more information on either sub-study, we refer to https://doi.org/10.13026/C2C30J and[12,13].

SHHS The *Sleep Heart Health Study* (SHHS) was a large, prospective cohort study investigating sleep-disordered breathing such as OSA as risk-factors for the development of cardiovascular disease[1,14]. A total of 6441 subjects were recruited from 6 already on-going National Heart, Lung, and Blood Institute studies (see https://clinicaltrials.gov/ct2/show/NCT00005275 for details). Adults of age 40 or older, who were able and willing to undergo home PSG, were enrolled between 1995–1998. Between 2001-2003, a second PSG was obtained for 3295 of the participants. For our study, we had a total of 8444 PSG records available (5793 visit 1; 2651 visit 2) collected from 5797 individuals. EEG and EOG signals were recorded at 125 Hz and 50 Hz, respectively, and hardware high-pass filtered at 0.15 Hz. Hypnograms were scored according to the Rechtschaffen and Kales criteria, which we aligned to AASM as described in the Methods section. For more information on SHHS, we refer to https://doi.org/10.25822/ghy8-ks59.

SOF A sub-study of the larger *Study of Osteoporotic Fractures* (SOF) investigated the association between sleep-disordered breathing and cognitive impairment in community-dwelling Caucasian women aged 65 and above[1,15,16]. Subjects were enrolled from four US cities between 1986–1988. An additional cohort of African-American women were recruited between 1997–1998. In our study, we considered the unattended, whole-night, in-home PSG data collected between 2002–2004 from 461 participants at SOF visit 8 (subjects originally enrolled from US metropolitan areas Minneapolis, Minnesota and and Pittsburgh, Pennsylvania between 1986-1988). EEG and EOG signals were recorded at 128 Hz and hardware high-pass filtered at 0.15 Hz. Hypnograms were scored according to the Rechtschaffen and Kales criteria, which we aligned to AASM as described in the Methods section. For more information, we refer to https://doi.org/10.13026/C2X676.

DCSM This new dataset was collected and prepared by the Danish Centre for Sleep Medicine (DCSM) and comprises 255 whole-night PSG recordings of patients visiting the center for diagnosis of non-specific sleep related disorders. The records are fully anonymized and were selected randomly. The included subjects thus likely vary in demographic characteristics, diagnostic background and sleep/non-sleep related medication usage. The PSGs were collected between 2015-2018. EEG and EOG signals were recorded at 256 Hz and bandpass filtered to interval 0.3 Hz - 70 Hz (3dB limits). Hypnograms were scored according to the AASM criteria. This dataset serves as an unbiased, random sample from the distribution of data generated by the DCSM. The DCSM dataset is publicaly available at https://sid.erda.dk/wsgi-bin/ls.py?share_id=fUH3xbOXv8. This repository will be frozen and issued a DOI for persistent access following the review process.

ISRUC-SG1, ISRUC-SG2 & ISRUC-SG3 The ISRUC dataset consists of randomly selected all-night PSG recordings acquired by the Sleep Medicine Centre of the Hospital of Coimbra University, Portugal[17]. It covers both healthy subjects and patients with sleep disorders under the effect of sleep medication. It is divided into three sub-groups (ISRUC-SG1, -SG2, -SG3) with 100 sleep disordered adults, 8 sleep disordered adults with PSGs aqquired twice on different nights, and 10 healthy control subjects in each of the three sub-groups, respectively. Data were aqquired between 2009–2013. All records were scored by two experts. We considered hypnograms from `annotator 1` for all records but `subject_2_visit_2` of `ISURC-SG2` for which we used the hypnogram of `annotator 2` due to missing data. EEG and EOG signals were recorded at 200 Hz and filtered using a bandpass Butterworth filter with lower and higher cutoff frequencies of 0.3 Hz and 35 Hz, respectively. Hypnograms were scored according to the AASM criteria. For more information on the ISRUC dataset, we refer to `https://sleeptight.isr.uc.pt`.

MASS-C1 & MASS-C3 The *Montreal Archive of Sleep Studies* (MASS) pooled 200 whole-night recordings from three different hospital-based sleep laboratories of the Center for Advanced Research in Sleep Medicine, Montreal, Canada[18]. Subjects were between 18–76 years at the time of recording, which occurred in the period 2001–2013. The subjects were organized into five subsets (C1-C5) according to the research protocols used for data acquisition. All included subjects were healthy controls, although an apnea-hypnea index of up to 20 (moderate sleep apnea) was allowed for subjects in C1. In this study, we considered PSG recordings from subsets C1 (53 subjects) and C3 (62 subjects) for which the experts annotated 30-second intervals in line with the other datasets. EEG and EOG signals were recorded at 256 Hz and hardware low-passed filtered at 0.10 Hz (EOG) or 0.30 Hz (EEG) and high-pass filtered at 30 Hz (EOG) or 100 Hz (EEG). Hypnograms were scored according to the AASM criteria. For more information, we refer to `http://ceams-carsm.ca/en/MASS`.

SVUH The *St. Vincent's University Hospital / University College Dublin Sleep Apnea Database* (SVUH) contains 25 full overnight PSG records of randomly selected individuals under diagnosis for either obstructive sleep apnea, central sleep apnea or primary snoring[12]. Subjects were enrolled over a 6-month period. We considered data from the revised database of 2001. Subjects were at least 18 years old and had no known cardiac disease, had no autonomic dysfunction, and took no medication known to interfere with heart rate. EEG and EOG signals were recorded at 128 Hz. Hypnograms were scored according to the Rechtschaffen and Kales criteria, which we aligned to AASM as described in the Methods section. For more information, we refer to `https://doi.org/10.13026/C26C7D`.

DOD-H & DOD-O The *Dreem Open Dataset – Healthy* (`DOD-H`) was collected from 25 volunteers at the French Armed Forces Biomedical Research Institute's Fatigue and Vigilance Unit in France. Subjects were without sleep complaints, aged 18-65 and locally recruited without regard to gender or ethnicity. The *Dreem Open Dataset – Obstructive* (`DOD-O`) was collected from the Stanford Sleep Medicine Center, California, US from 55 patients (clinical trial NCT03657329) with clinical suspicion for sleep-related breathing disorder. Individuals clinically diagnosed with sleep disorders other than OSA, suffering from morbid obesity, taking sleep medications or with certain cardiopulmonary or neurological comorbidities were exclude from the study. EEG and EOG signals from both `DOD-H` and `DOD-O` were sampled at 256 Hz and each PSG was scored by 5 individual experts from 3 different sleep clinics. All experts were registered Sleep Technologists with at least 5 years of clinical scoring experience. For more information on the `DOD` datasets and consensus scoring, we refer to the recent publications[19–21].

Demographic Bias

We conducted an analysis of potential demographic bias in the average U-Sleep performance. We considered the variables age, sex and BMI and accounted for dataset origin. We could not evaluate important variables such as disease state and ethnicity, because the required information was missing was missing for several datasets. Supplementary Figure 1 shows graphical representations of the test-set distribution of F1 scores as a function of age, sex, BMI and general disease stage, respectively. Note that these plots show only correlations, not causal relations.

We fitted a beta regression model (using the `betareg`[22] v3.1-3 package in `R`[23] v3.6.1) on 532 records from the test sets of datasets ABC, CCSHS, CFS, CHAT, HPAP, MROS, SHHS, SOF and SVUH. The 532 records represent all available test-set records for which we have age, sex and BMI information available. The regression model predicts the mean F1 score as a function of those parameters along with variables encoding the dataset origin of each sample giving a total 11 covariates. The estimated coefficients of the model were -0.004 ± 0.007 for BMI (95% CI, $z = -1.273$, $p = 0.203$), -0.012 ± 0.004 for age (95% CI, $z = -6.141$, $p < 0.001$), and -0.102 ± 0.102 for sex (difference if subject is Male, 95% CI, $z = -1.954$, $p = 0.051$). Coefficients testing were done using two-sided Z-tests. The interpretation of the coefficients is that the expected F1 performance drops with increasing BMI and increasing age as well as for male subjects. However, only age was significant ($p < 0.05$). It is likely that this observation is confounded by the general worsening of health with age.

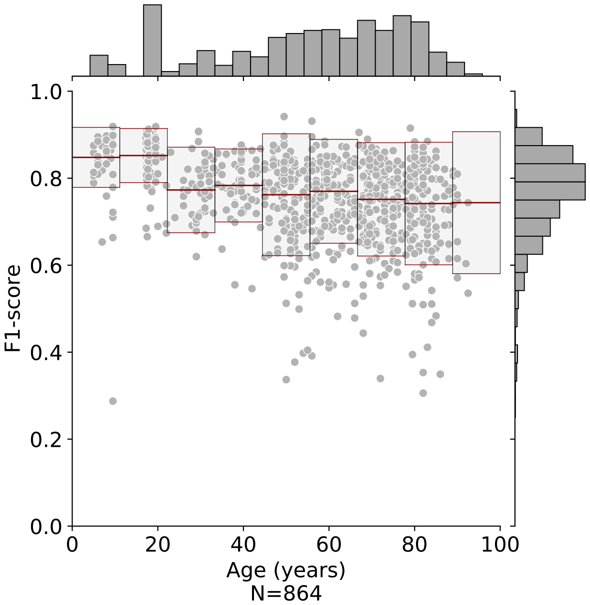

(a) Majority vote mean F1-scores by subject age.

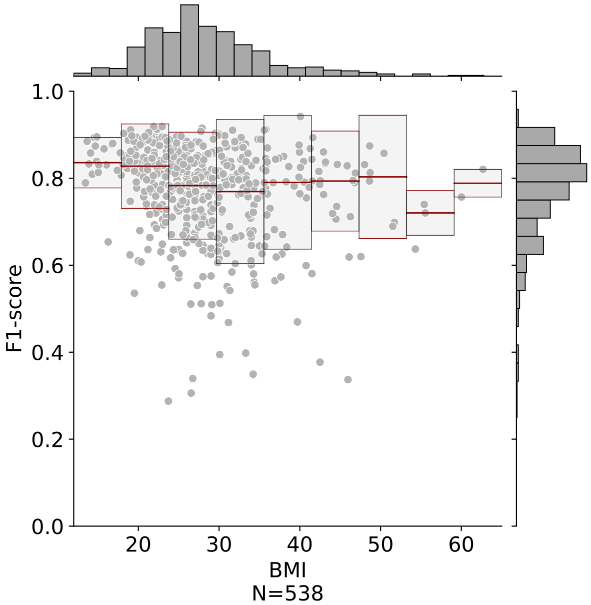

(b) Majority vote mean F1-scores by subject BMI.

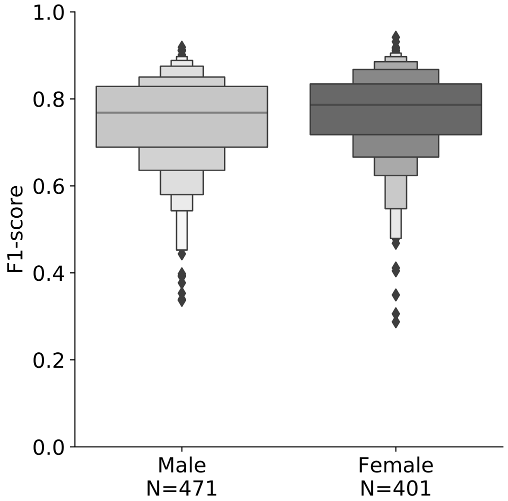

(c) Majority vote mean F1-scores by subject sex.

Figure 1: Correlations between U-Sleep per-subject mean F1 score performance (using majority votes) and individual demographic variables. In panels (a) and (b) the red center lines show median values in 10 equally sized bins. The lower and upper red lines together represent the interquartile range. In panel (c) letter-value plots[24] visualize the median (center black line) and 10 other quantiles (letter-values, specifically). Observations beyond the most extreme letter-values are labeled outliers and plotted as diamond shapes. Note that the widths of each box in the letter-value plots are arbitrary and serve only to visually separate individual boxes. Please refer to the Supplementary Note: Demographic Bias for statistical analysis and discussion.

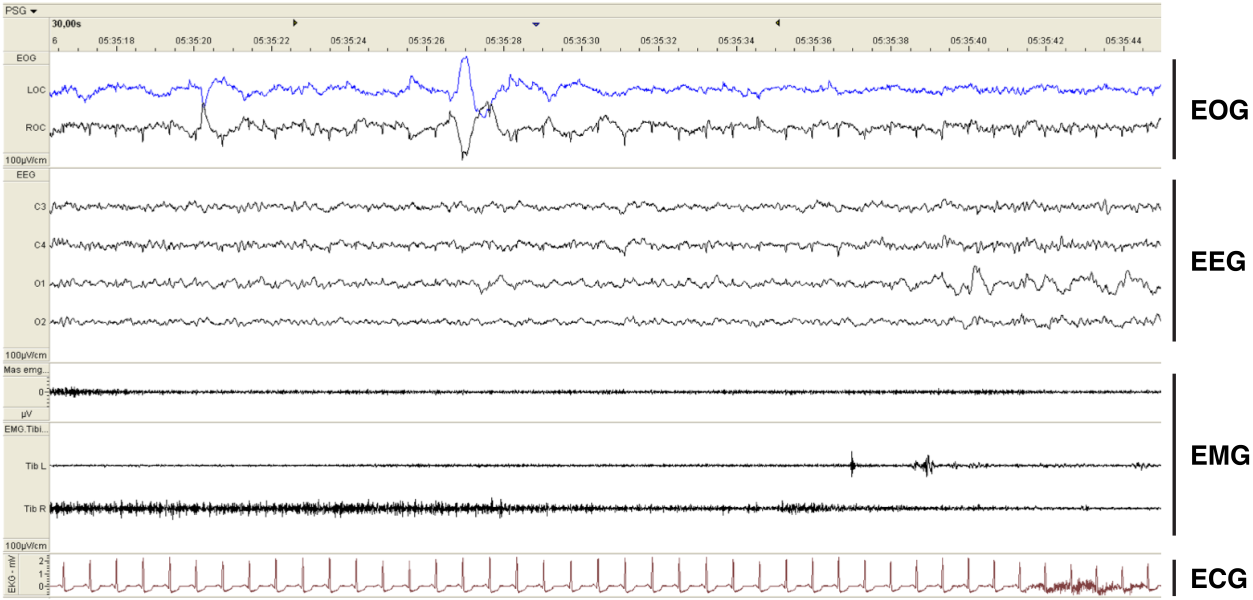

Figure 2: A segment of 30 seconds of a typical polysomnography (PSG) study showing multiple EOG, EEG, EMG and ECG channels. Human experts inspect segments such as this and assign it to one of the sleep stages in {W, N1, N2, N3, R}. U-Sleep requires only a single EEG channel and a single EOG channel (e.g., C3 and LOC above). Figure modified from Perslev et al.[26].

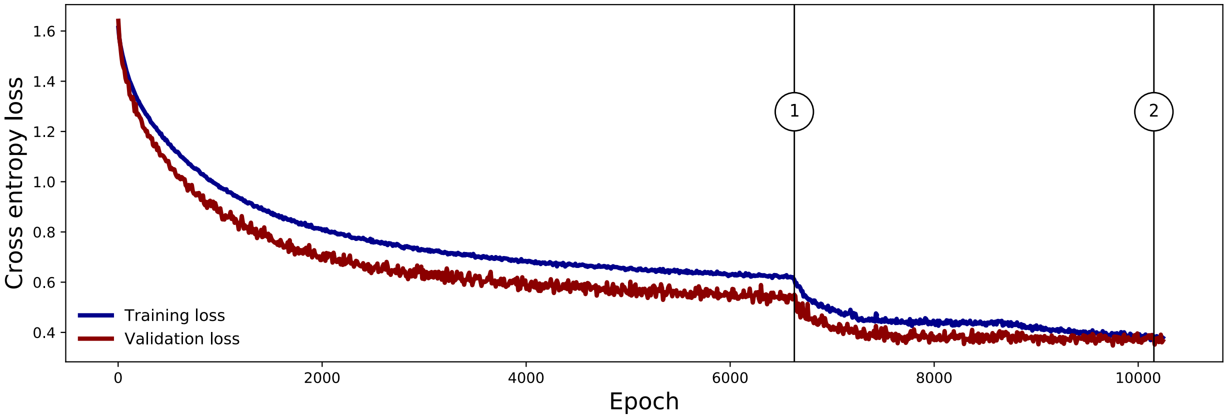

(a) The training and validation loss on the training and validation set. Little to no overfitting (reduced training loss with stagnant or increasing validation loss) is observed. Two points of interest are marked: 1) The sudden improvement in performance occurs as the model starts improving on the difficult N1 sleep stage. Up until this point, U-Sleep would rarely predict N1 stages at all, resulting in a lower mean performance. 2) The finally selected model at epoch 10154. Training for 150 epochs after this point did not further improve validation performance.

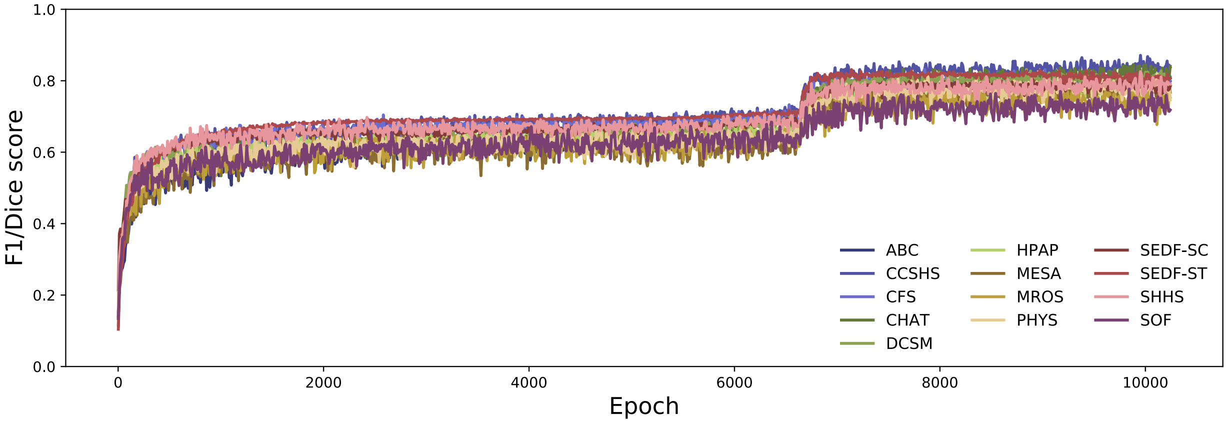

(b) Mean F1 score computed across all datasets using random subsets of the validation data after each epoch of training. The mean F1 increases steadily over time on all datasets, indicating that the model is able to simultaneously learn the function across all clinical cohorts.

Figure 3: U-Sleep learning curves. It took a total of $\approx 4,500,000$ gradient updates (processed batches of data) to train the model to convergence, equivalent to observing $\approx 9,582$ years of (non-unique) annotated PSG data. The total training set length is ≈ 19.4 years. The long training time needed to obtain the final model is a result of both the highly challenging task – learning sleep staging across clinical cohorts with varying and noisy labels, randomly varying input channels as well as augmentation – and that we chose to train U-Sleep using a very small learning rate (please refer to the Methods section). As we were interested only in a single, final version of the U-Sleep model, the long training time is only an issue because of the energy consumption. We estimate that training U-Sleep consumed up to a total of $1,121$ kWh (96.1 kg CO_2 eq.) using the `CarbonTracker` tool[25] with an added 25 % margin.

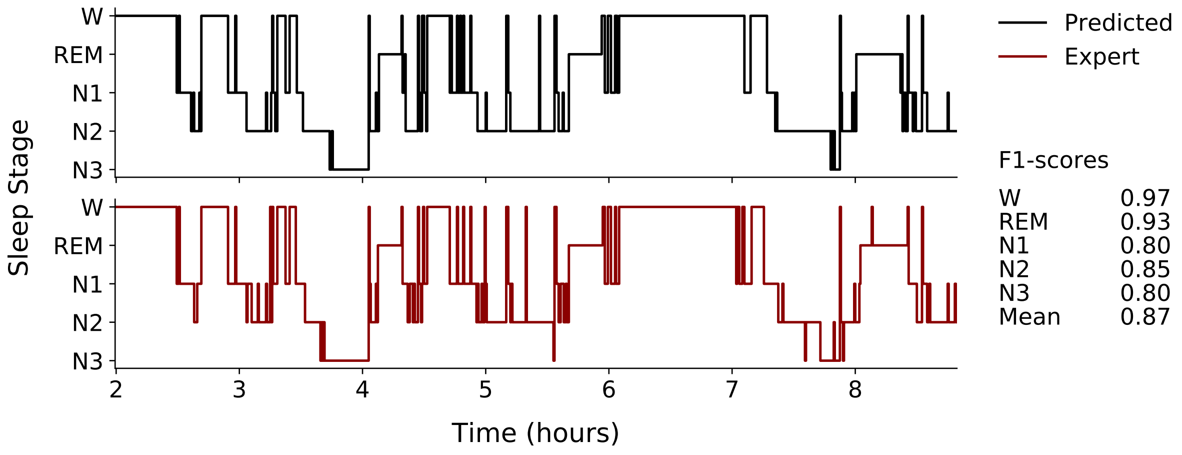

(a) Hypnogram with highest observed F1-score (record `abc-baseline-900026`).

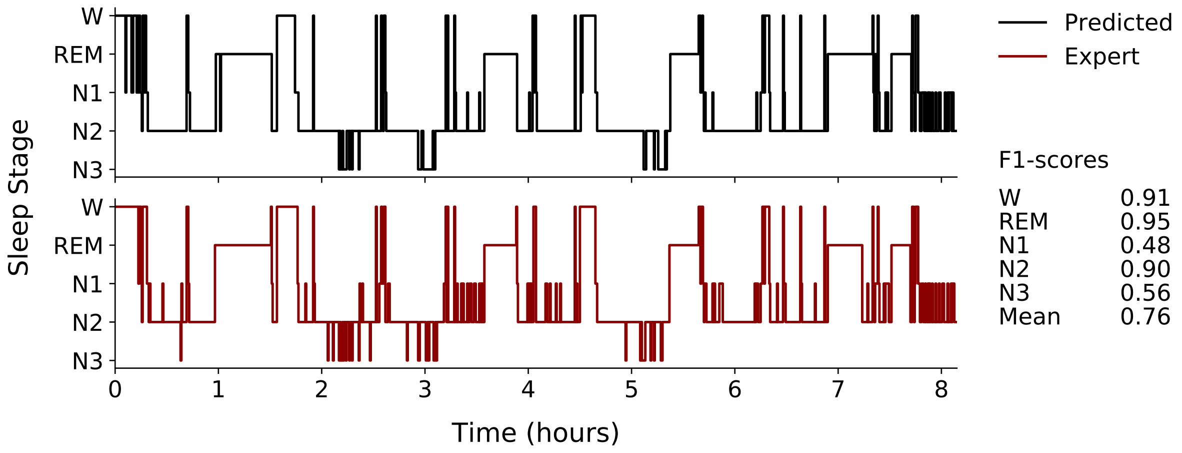

(b) Hypnogram with F1-score nearest dataset median (record `abc-baseline-900039`).

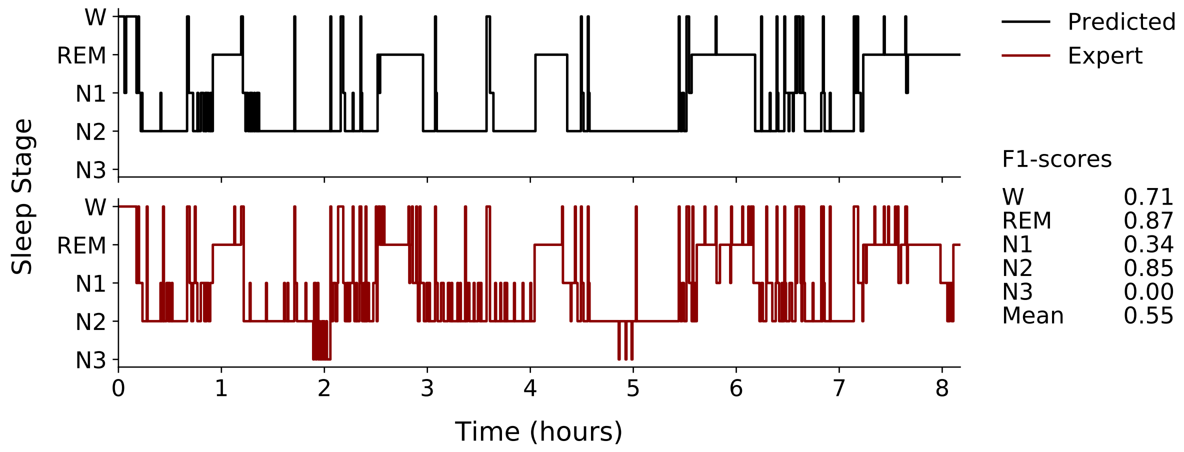

(c) Hypnogram with lowest observed F1-score (record `abc-baseline-900014`).

Figure 4: Highest, nearest median and lowest scoring (majority voted) hypnograms observed across records in the test-split of dataset `ABC`. Black hypnograms were predicted by U-Sleep, red hypnograms are human expert annotations. F1-scores for each stage are shown to the right of each set of hypnograms. Each hypnogram displays at most 30 minutes of wake prior to and following the first and last non-wake period, respectively, as determined by the human expert annotations.

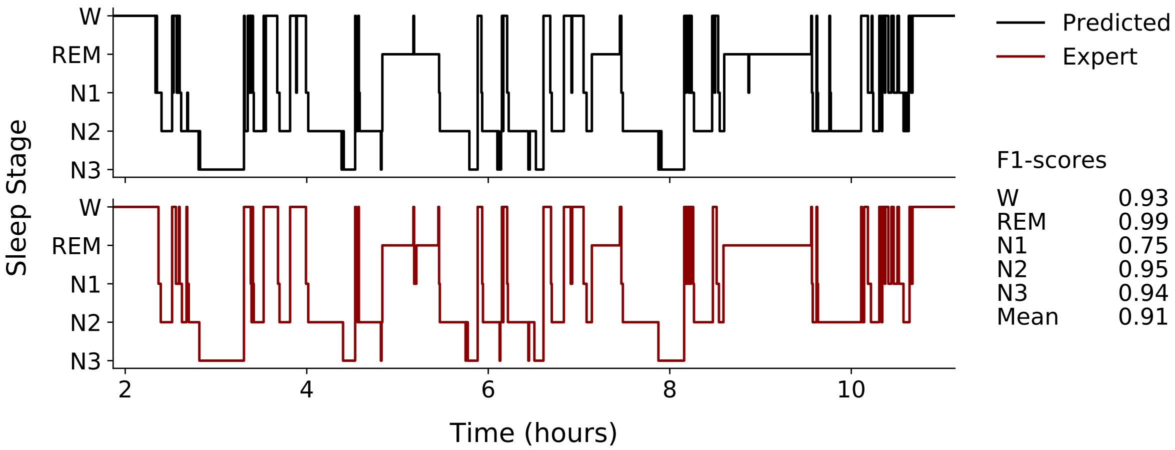

(a) Hypnogram with highest observed F1-score (record `ccshs-trec-1800544`).

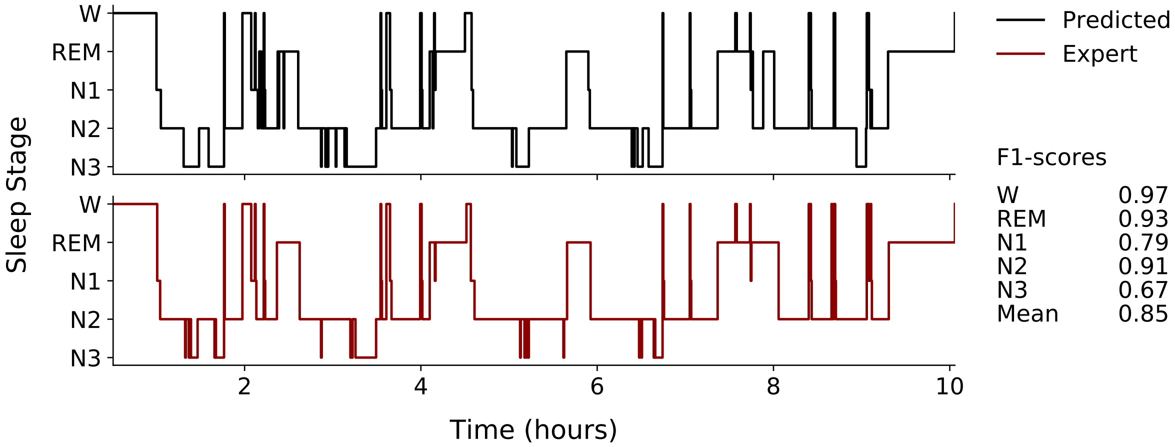

(b) Hypnogram with F1-score nearest dataset median (record `ccshs-trec-1800195`).

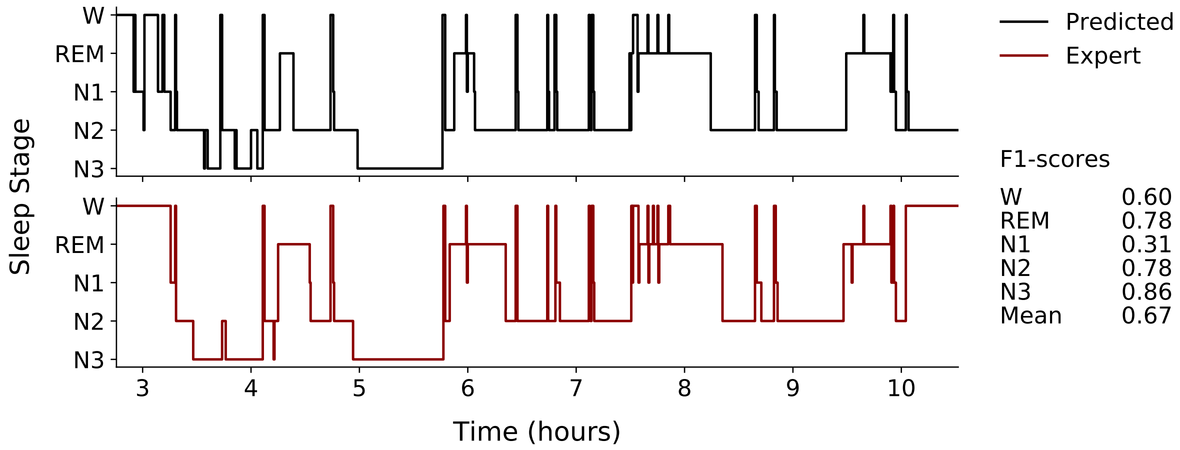

(c) Hypnogram with lowest observed F1-score (record `ccshs-trec-1800007`).

Figure 5: Highest, nearest median and lowest scoring (majority voted) hypnograms observed across records in the test-split of dataset `CCSHS`. Black hypnograms were predicted by U-Sleep, red hypnograms are human expert annotations. F1-scores for each stage are shown to the right of each set of hypnograms. Each hypnogram displays at most 30 minutes of wake prior to and following the first and last non-wake period, respectively, as determined by the human expert annotations.

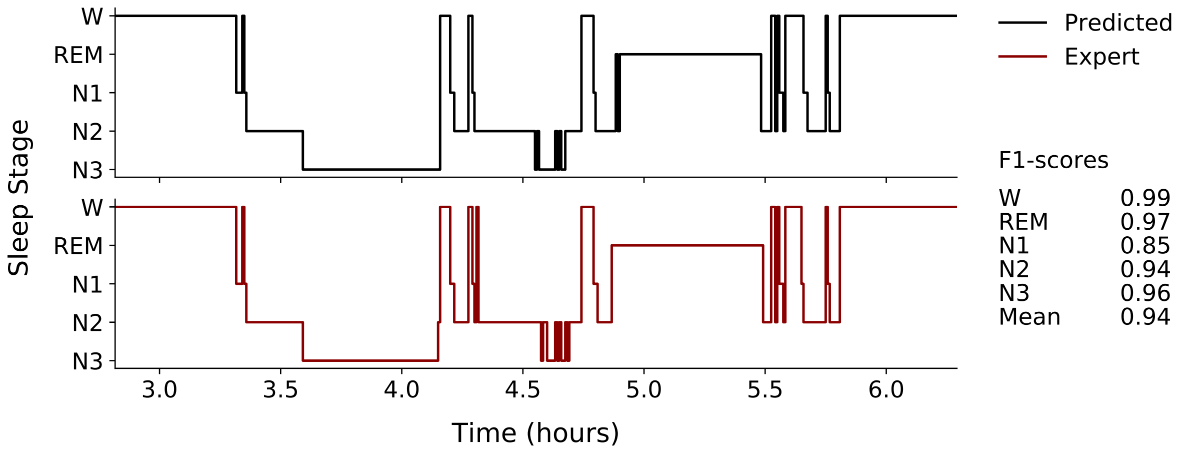

(a) Hypnogram with highest observed F1-score (record `cfs-visit5-802273`).

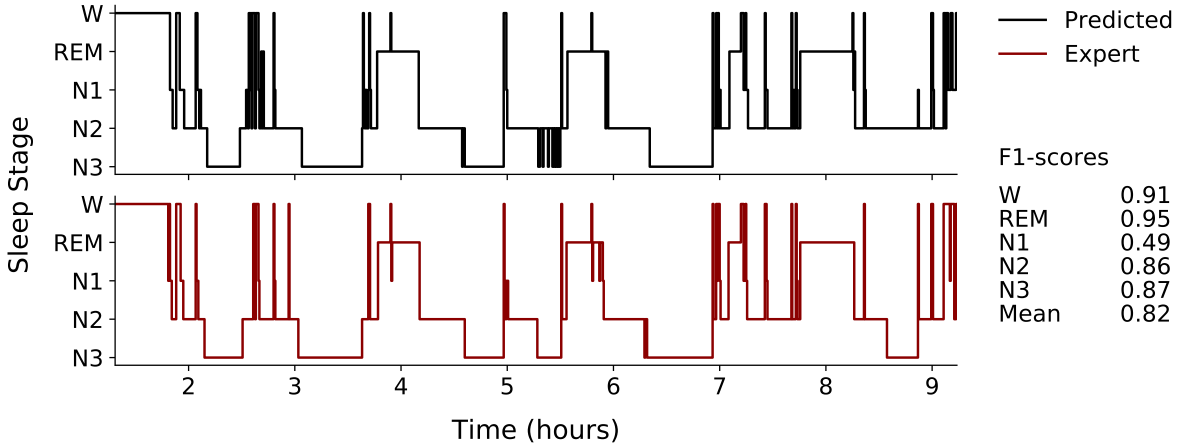

(b) Hypnogram with F1-score nearest dataset median (record `cfs-visit5-802658`).

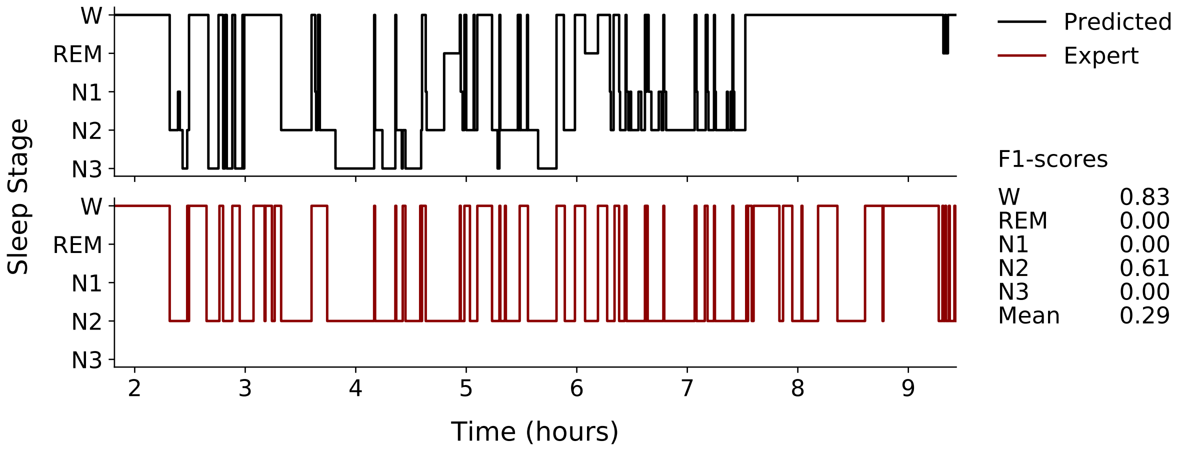

(c) Hypnogram with lowest observed F1-score (record `cfs-visit5-802299`).

Figure 6: Highest, nearest median and lowest scoring (majority voted) hypnograms observed across records in the test-split of dataset `CFS`. Black hypnograms were predicted by U-Sleep, red hypnograms are human expert annotations. F1-scores for each stage are shown to the right of each set of hypnograms. Each hypnogram displays at most 30 minutes of wake prior to and following the first and last non-wake period, respectively, as determined by the human expert annotations.

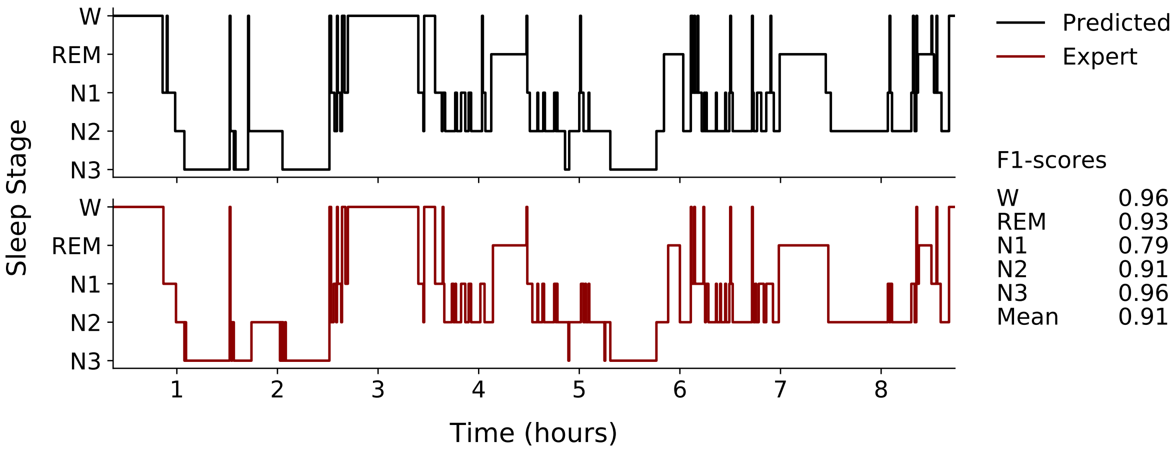

(a) Hypnogram with highest observed F1-score (record `chat-baseline-nonrandomized-300405`).

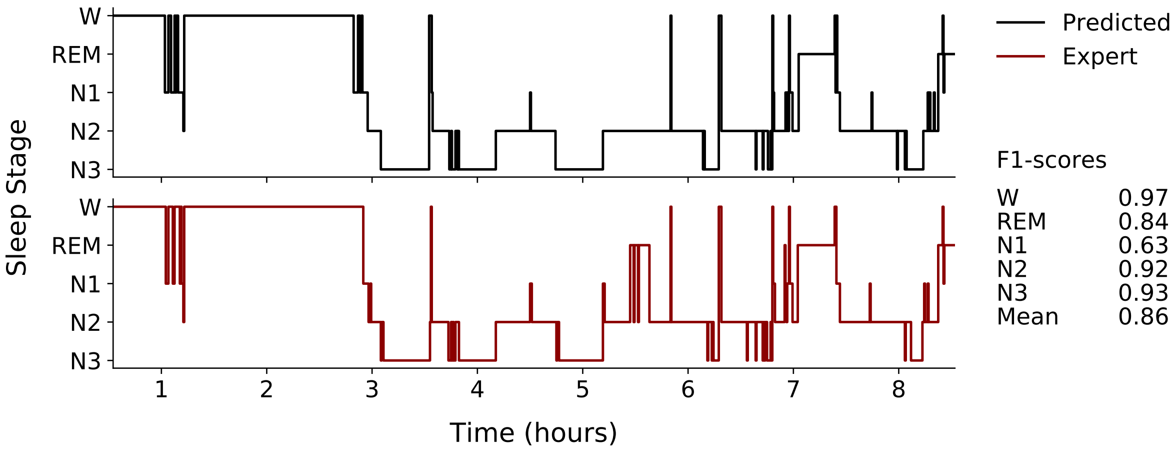

(b) Hypnogram with F1-score nearest dataset median (record `chat-baseline-nonrandomized-301034`).

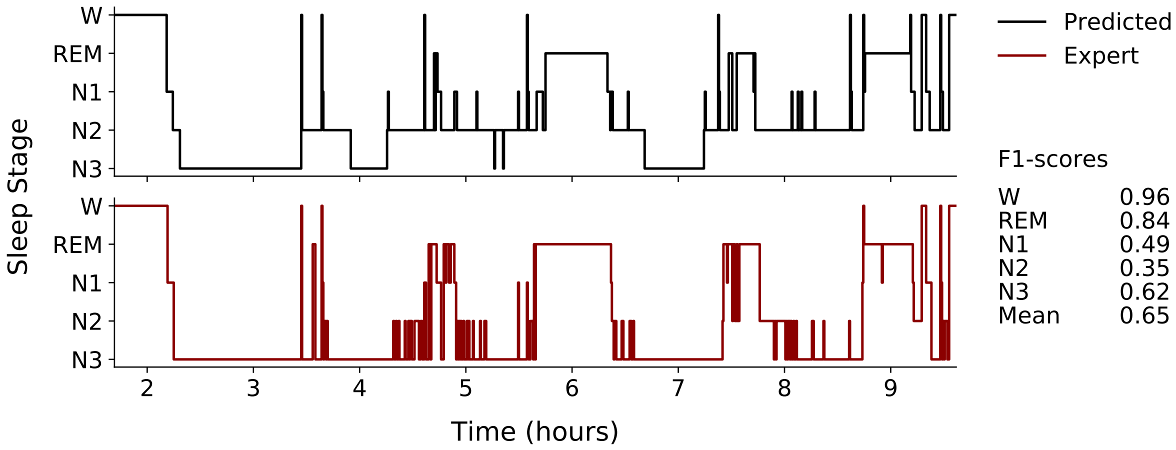

(c) Hypnogram with lowest observed F1-score (record `chat-baseline-300397`).

Figure 7: Highest, nearest median and lowest scoring (majority voted) hypnograms observed across records in the test-split of dataset `CHAT`. Black hypnograms were predicted by U-Sleep, red hypnograms are human expert annotations. F1-scores for each stage are shown to the right of each set of hypnograms. Each hypnogram displays at most 30 minutes of wake prior to and following the first and last non-wake period, respectively, as determined by the human expert annotations.

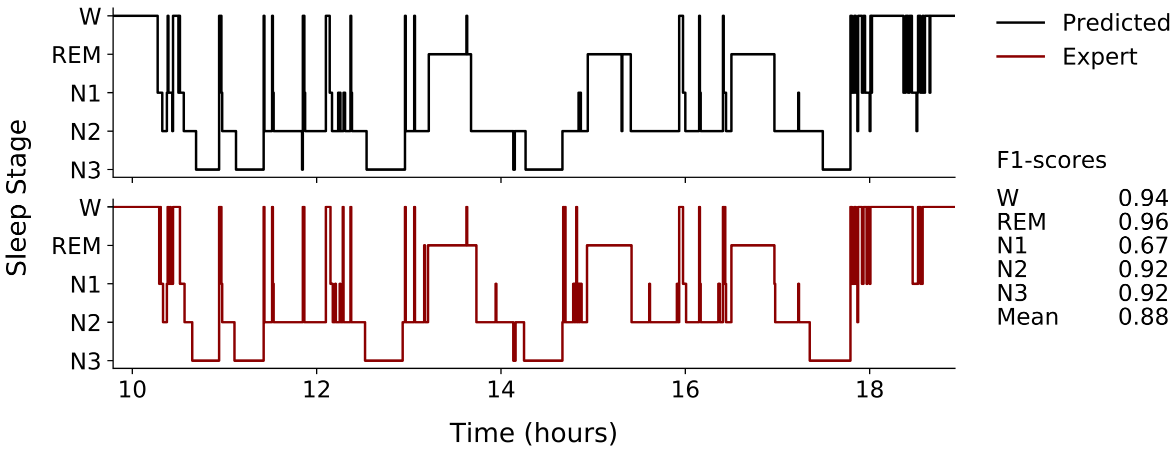

(a) Hypnogram with highest observed F1-score (record `285ab4bdf51f`).

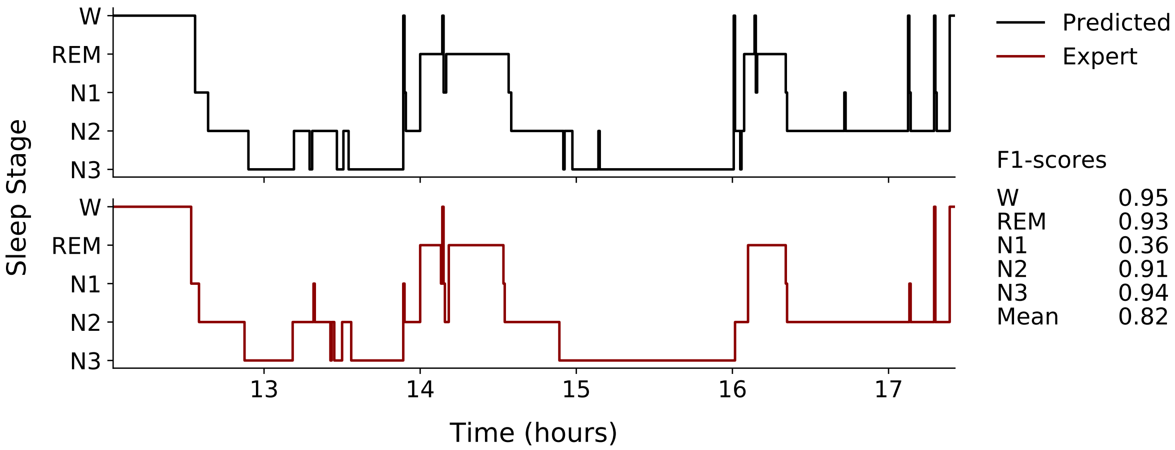

(b) Hypnogram with F1-score nearest dataset median (record `4dac221360bb`).

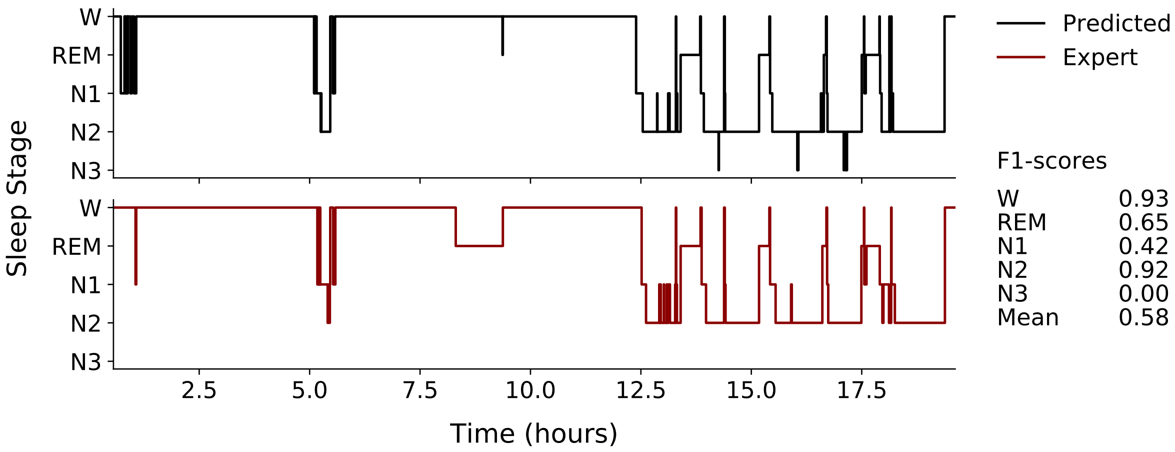

(c) Hypnogram with lowest observed F1-score (record `65fd36d709ae`).

Figure 8: Highest, nearest median and lowest scoring (majority voted) hypnograms observed across records in the test-split of dataset `DCSM`. Black hypnograms were predicted by U-Sleep, red hypnograms are human expert annotations. F1-scores for each stage are shown to the right of each set of hypnograms. Each hypnogram displays at most 30 minutes of wake prior to and following the first and last non-wake period, respectively, as determined by the human expert annotations.

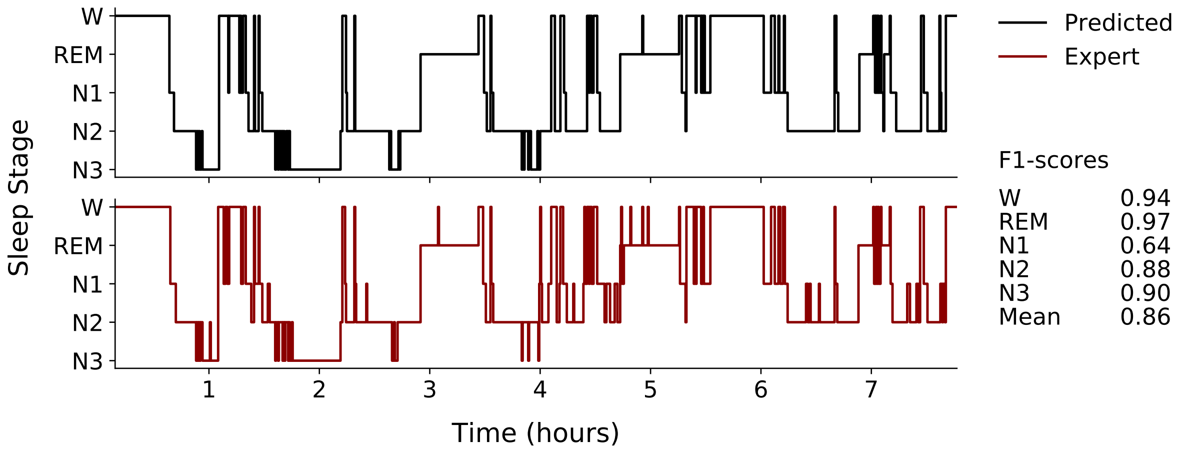

(a) Hypnogram with highest observed F1-score (record `homepap-lab-full-1600255`).

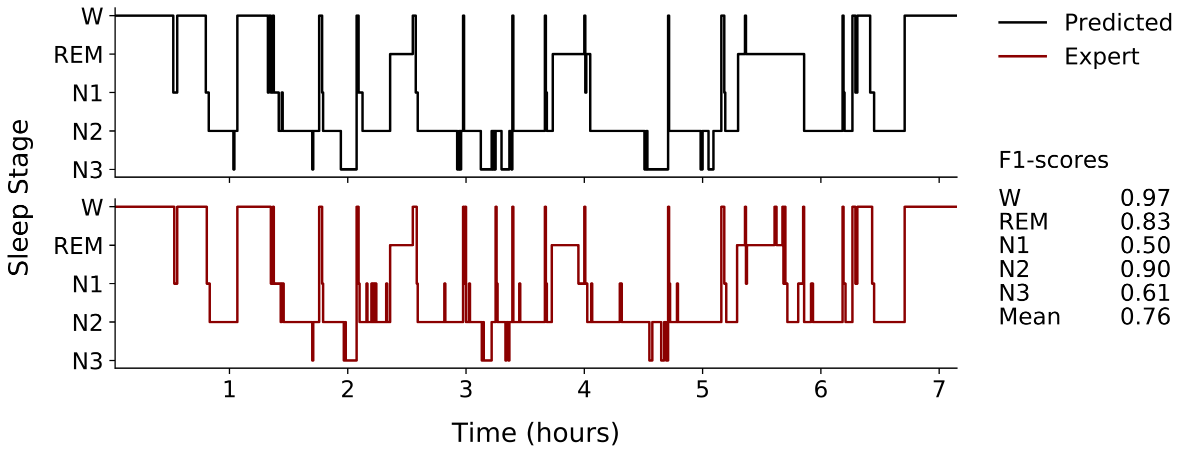

(b) Hypnogram with F1-score nearest dataset median (record `homepap-lab-full-1600319`).

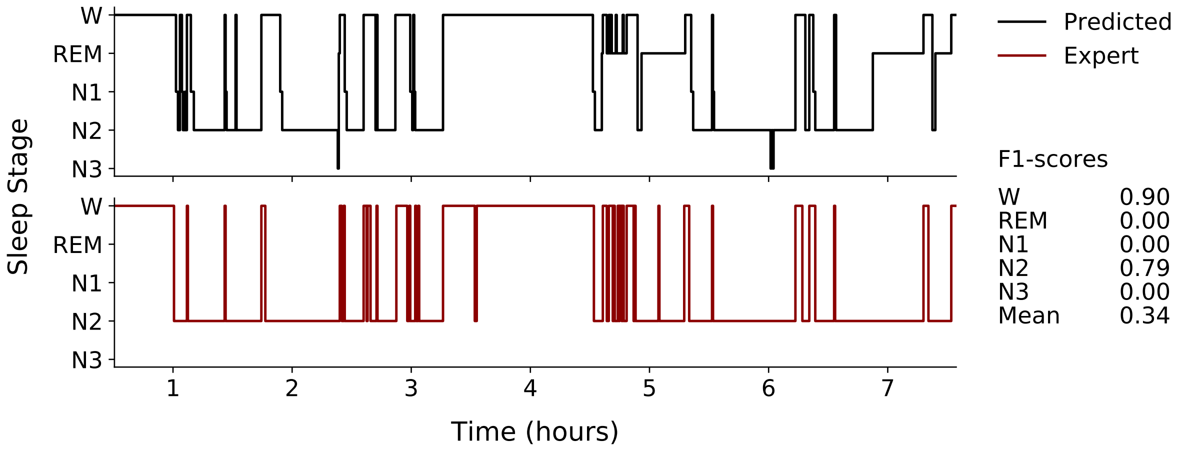

(c) Hypnogram with lowest observed F1-score (record `homepap-lab-split-1600251`).

Figure 9: Highest, nearest median and lowest scoring (majority voted) hypnograms observed across records in the test-split of dataset `HPAP`. Black hypnograms were predicted by U-Sleep, red hypnograms are human expert annotations. F1-scores for each stage are shown to the right of each set of hypnograms. Each hypnogram displays at most 30 minutes of wake prior to and following the first and last non-wake period, respectively, as determined by the human expert annotations.

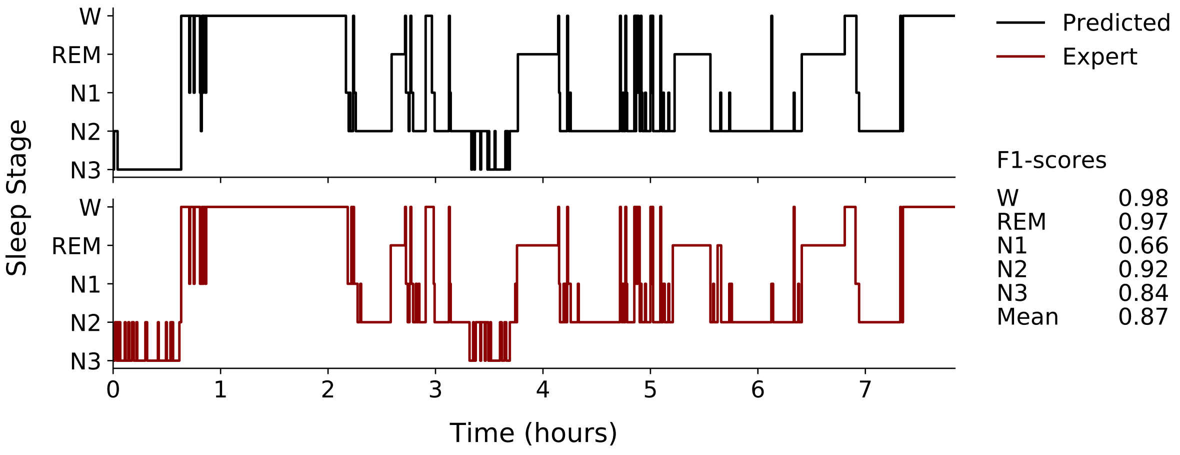

(a) Hypnogram with highest observed F1-score (record `mesa-sleep-4682`).

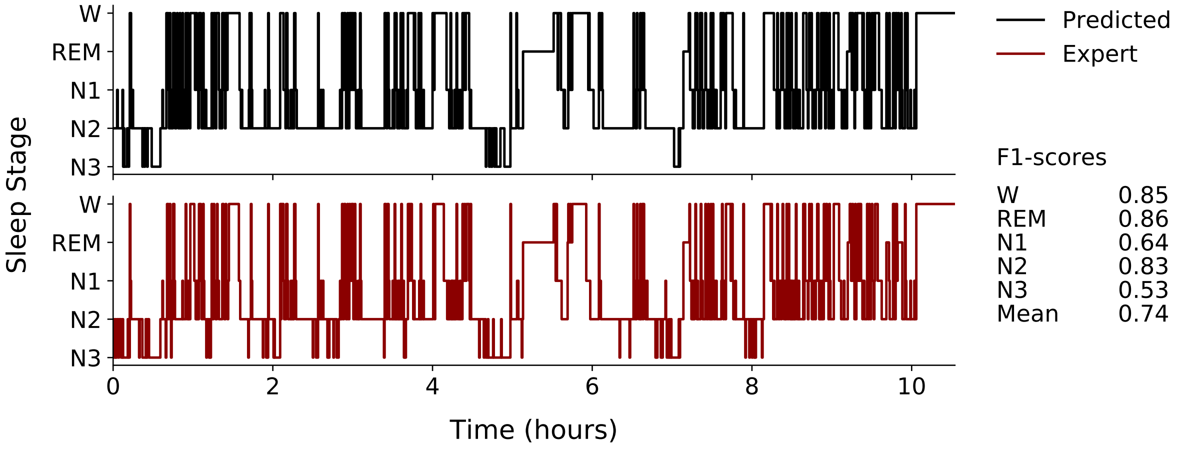

(b) Hypnogram with F1-score nearest dataset median (record `mesa-sleep-2834`).

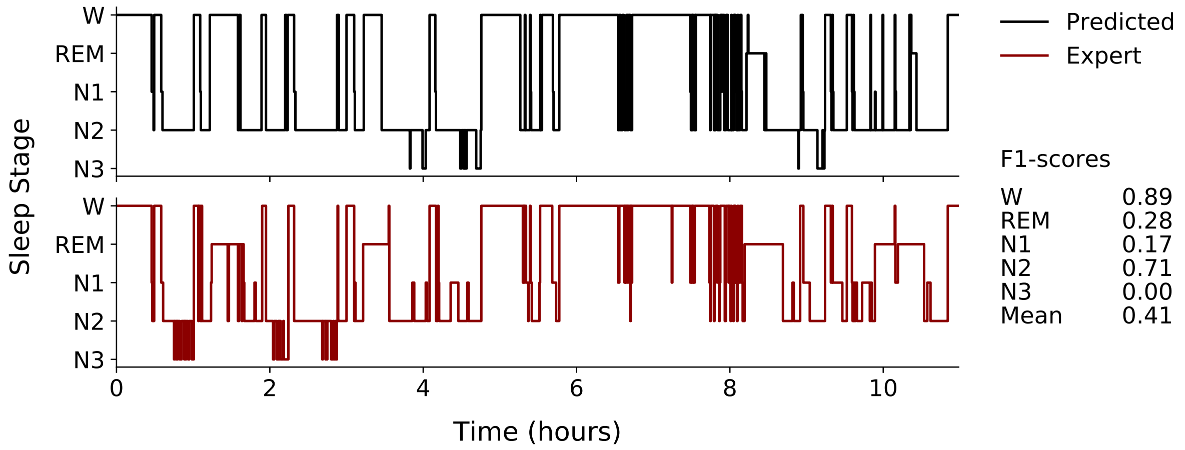

(c) Hypnogram with lowest observed F1-score (record `mesa-sleep-5680`).

Figure 10: Highest, nearest median and lowest scoring (majority voted) hypnograms observed across records in the test-split of dataset `MESA`. Black hypnograms were predicted by U-Sleep, red hypnograms are human expert annotations. F1-scores for each stage are shown to the right of each set of hypnograms. Each hypnogram displays at most 30 minutes of wake prior to and following the first and last non-wake period, respectively, as determined by the human expert annotations.

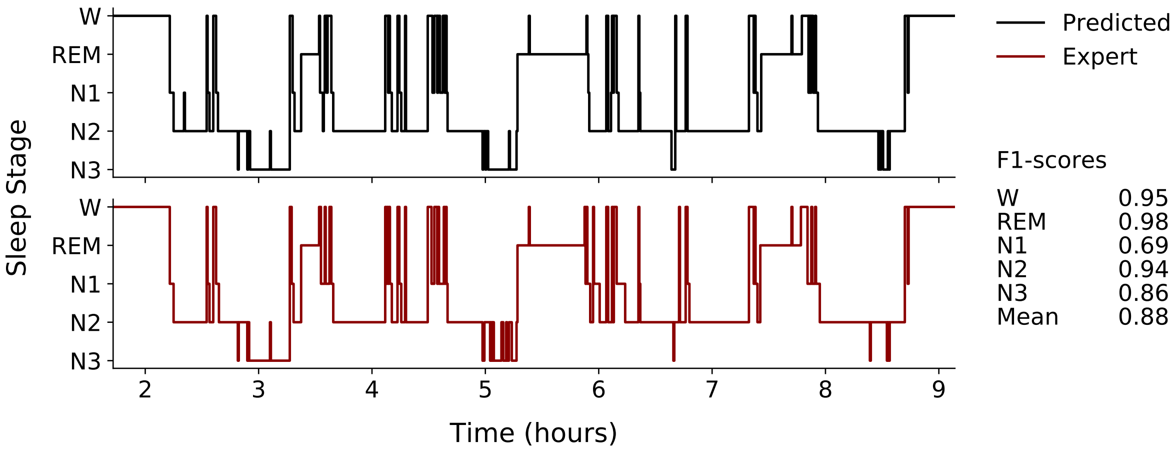

(a) Hypnogram with highest observed F1-score (record `mros-visit1-aa2023`).

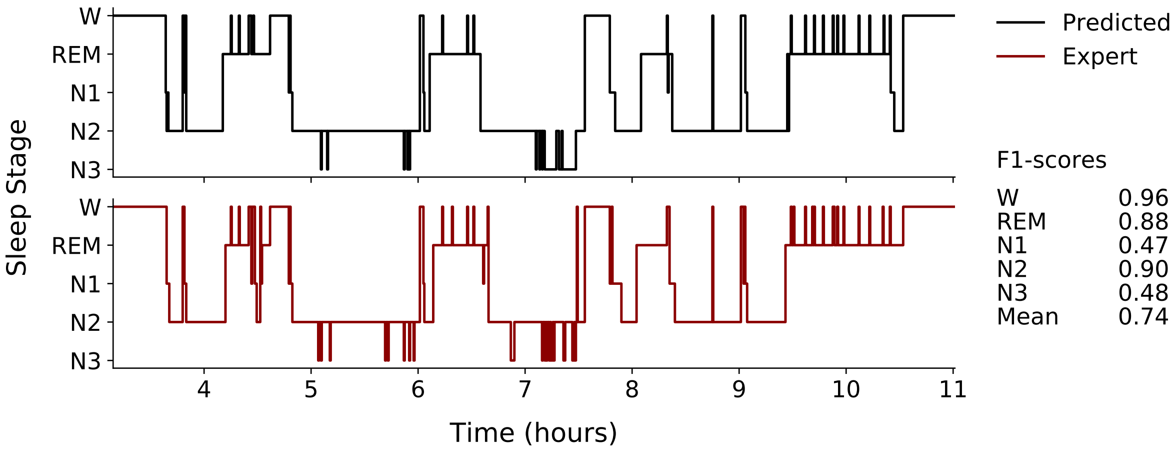

(b) Hypnogram with F1-score nearest dataset median (record `mros-visit1-aa2359`).

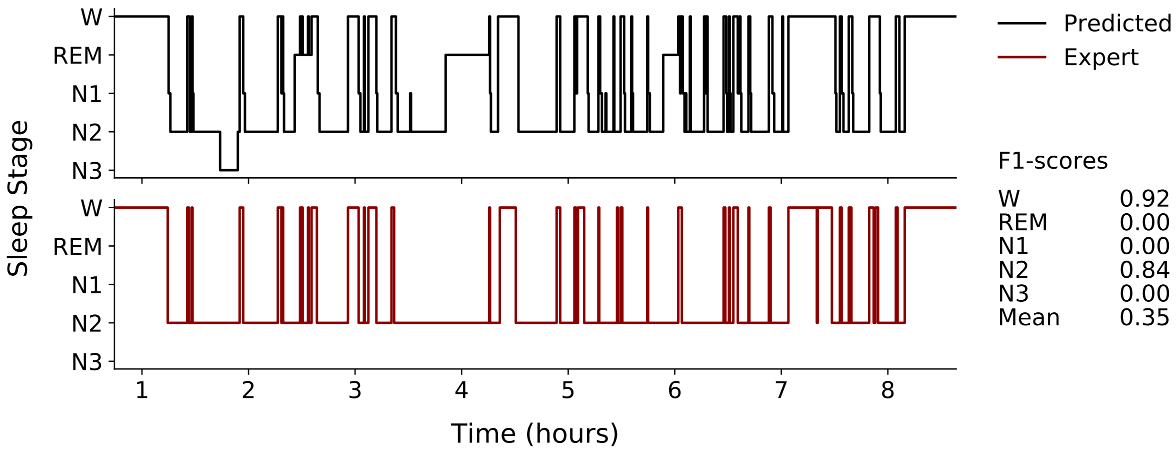

(c) Hypnogram with lowest observed F1-score (record `mros-visit2-aa3175`).

Figure 11: Highest, nearest median and lowest scoring (majority voted) hypnograms observed across records in the test-split of dataset `MROS`. Black hypnograms were predicted by U-Sleep, red hypnograms are human expert annotations. F1-scores for each stage are shown to the right of each set of hypnograms. Each hypnogram displays at most 30 minutes of wake prior to and following the first and last non-wake period, respectively, as determined by the human expert annotations.

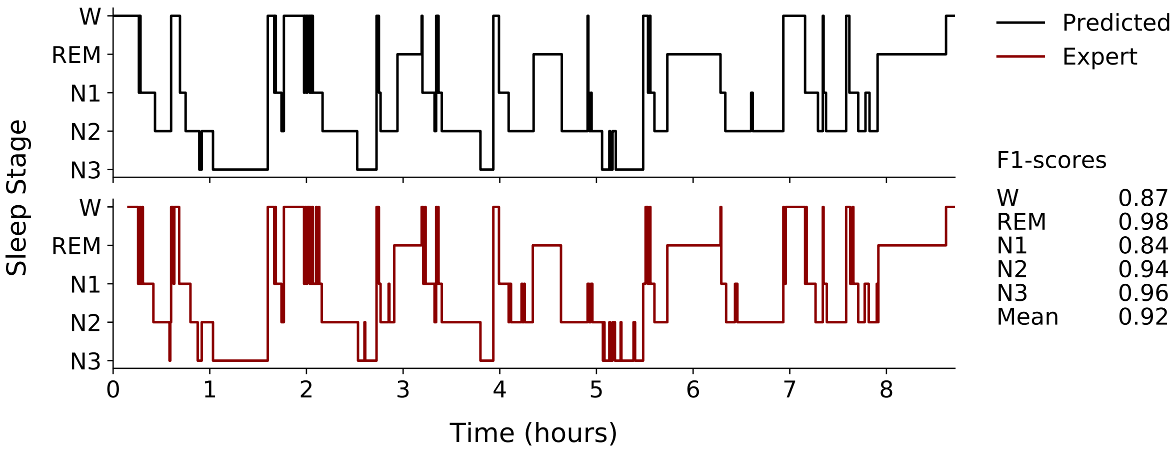

(a) Hypnogram with highest observed F1-score (record `tr07-0891`).

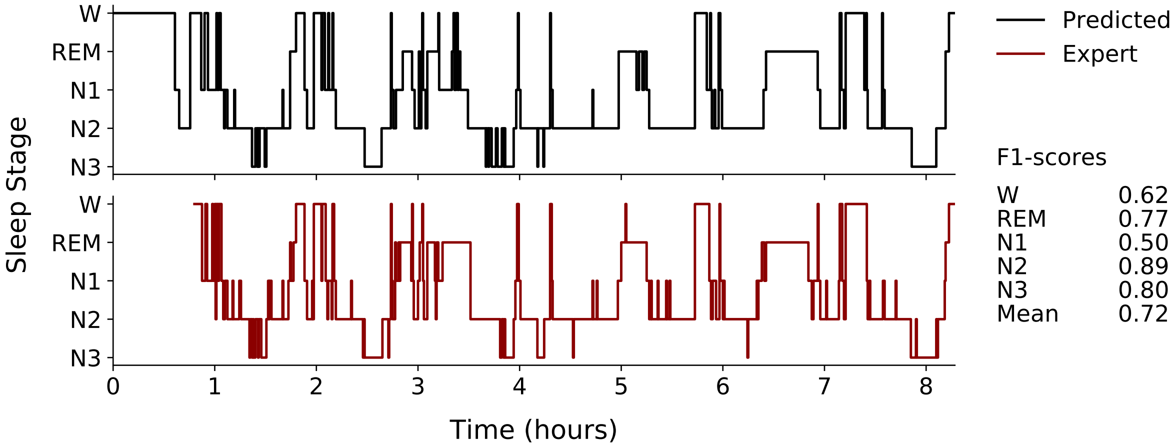

(b) Hypnogram with F1-score nearest dataset median (record `tr07-0394`).

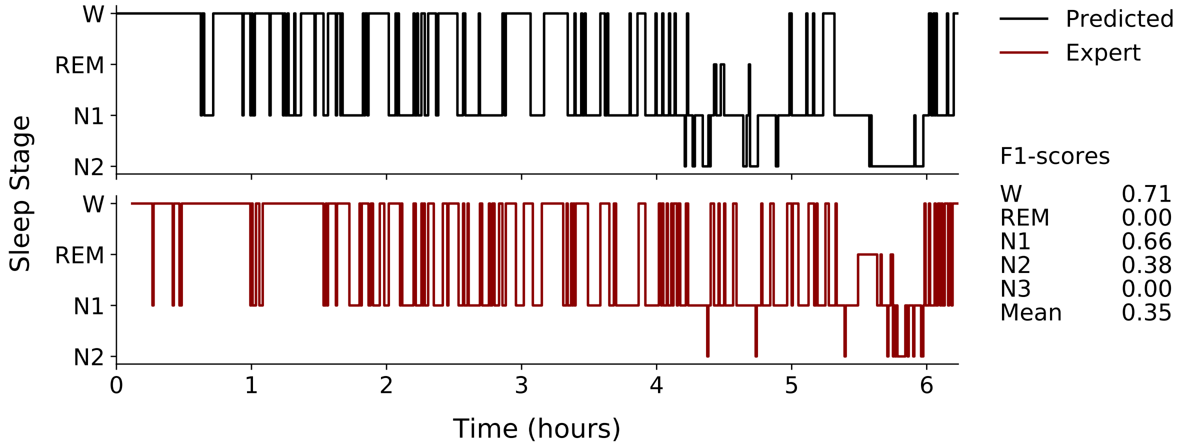

(c) Hypnogram with lowest observed F1-score (record `tr07-0828`).

Figure 12: Highest, nearest median and lowest scoring (majority voted) hypnograms observed across records in the test-split of dataset PHYS. Black hypnograms were predicted by U-Sleep, red hypnograms are human expert annotations. F1-scores for each stage are shown to the right of each set of hypnograms. Each hypnogram displays at most 30 minutes of wake prior to and following the first and last non-wake period, respectively, as determined by the human expert annotations.

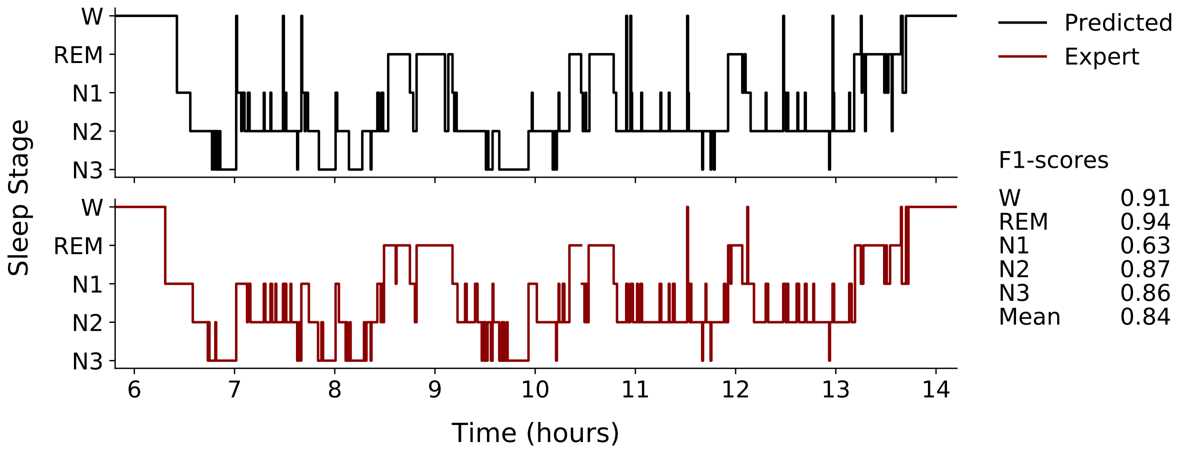

(a) Hypnogram with highest observed F1-score (record `SC4022E0-PSG`).

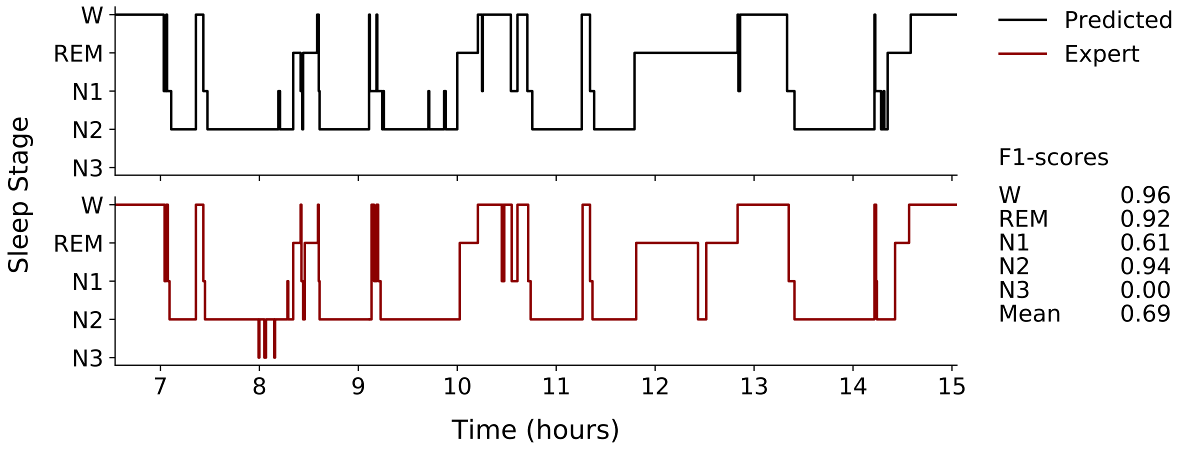

(b) Hypnogram with F1-score nearest dataset median (record `SC4201E0-PSG`).

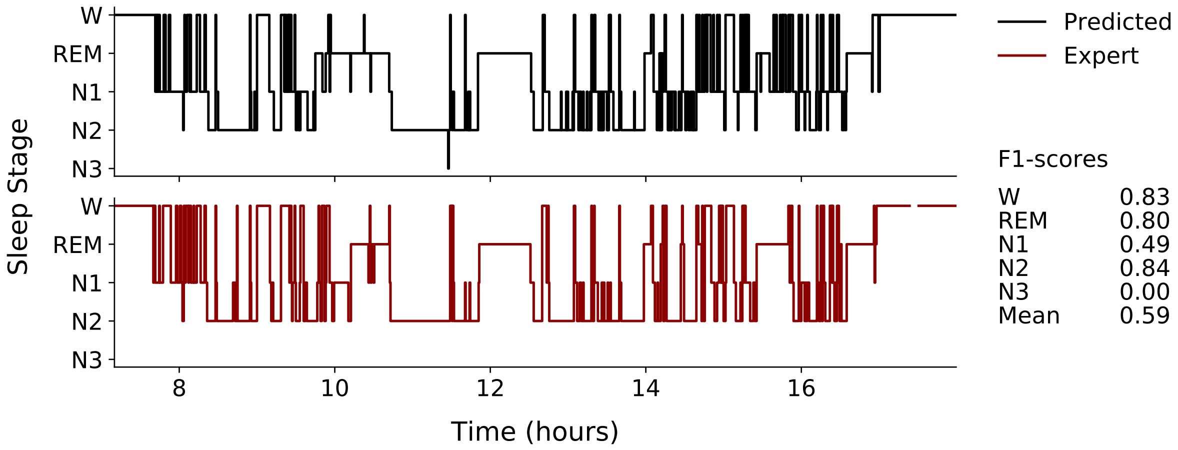

(c) Hypnogram with lowest observed F1-score (record `SC4571F0-PSG`).

Figure 13: Highest, nearest median and lowest scoring (majority voted) hypnograms observed across records in the test-split of dataset `SEDF-SC`. Black hypnograms were predicted by U-Sleep, red hypnograms are human expert annotations. F1-scores for each stage are shown to the right of each set of hypnograms. Each hypnogram displays at most 30 minutes of wake prior to and following the first and last non-wake period, respectively, as determined by the human expert annotations.

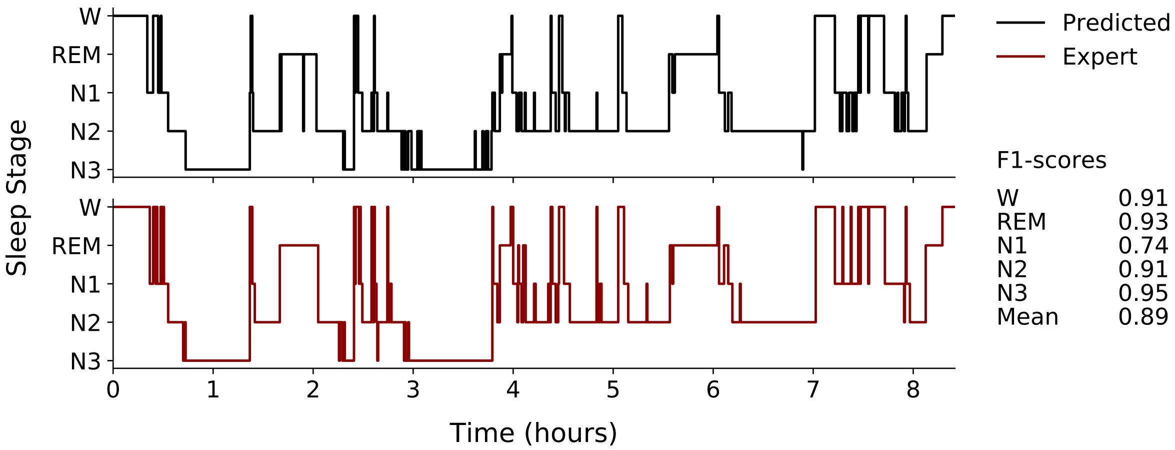

(a) Hypnogram with highest observed F1-score (record `ST7212J0-PSG`).

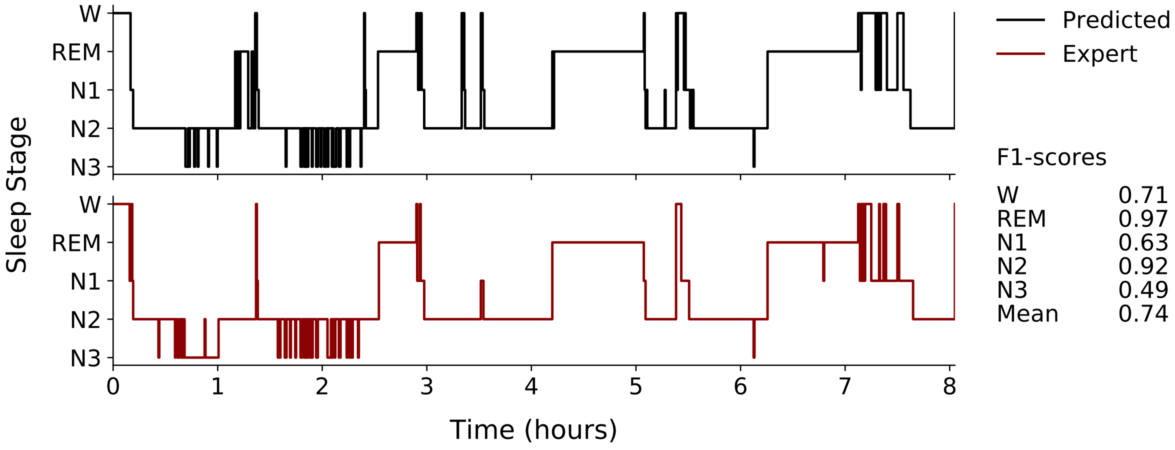

(b) Hypnogram with F1-score nearest dataset median (record `ST7182J0-PSG`).

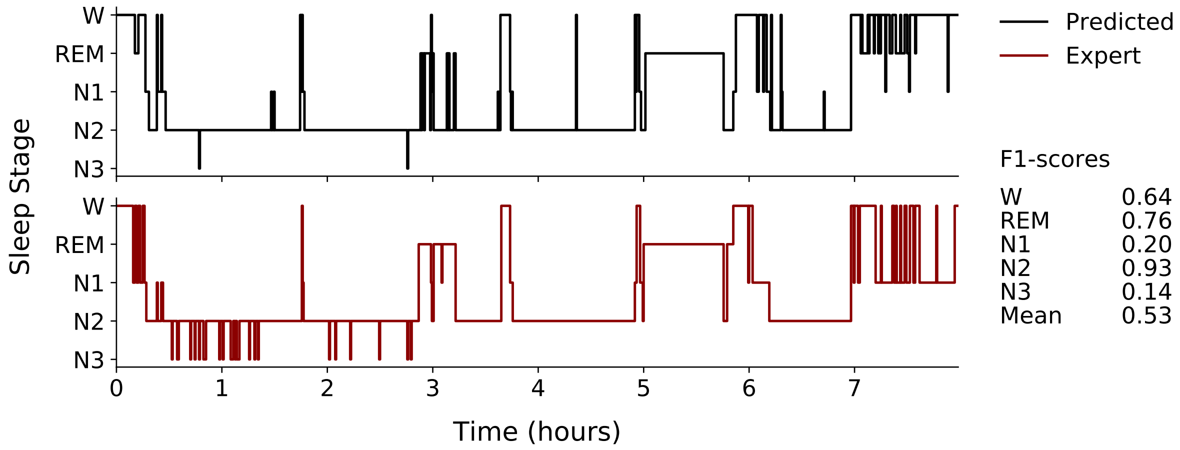

(c) Hypnogram with lowest observed F1-score (record `ST7181J0-PSG`).

Figure 14: Highest, nearest median and lowest scoring (majority voted) hypnograms observed across records in the test-split of dataset `SEDF-ST`. Black hypnograms were predicted by U-Sleep, red hypnograms are human expert annotations. F1-scores for each stage are shown to the right of each set of hypnograms. Each hypnogram displays at most 30 minutes of wake prior to and following the first and last non-wake period, respectively, as determined by the human expert annotations.

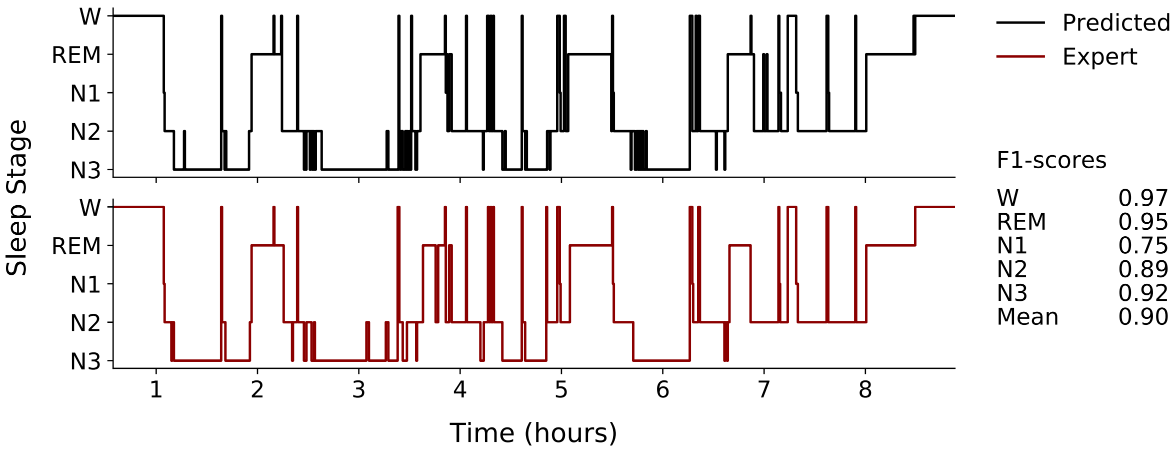

(a) Hypnogram with highest observed F1-score (record `shhs1-204781`).

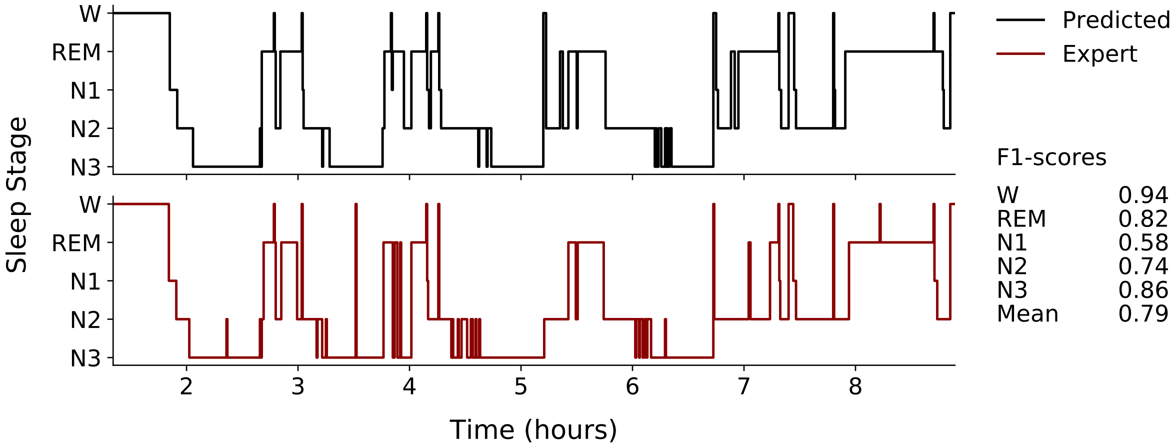

(b) Hypnogram with F1-score nearest dataset median (record `shhs1-204364`).

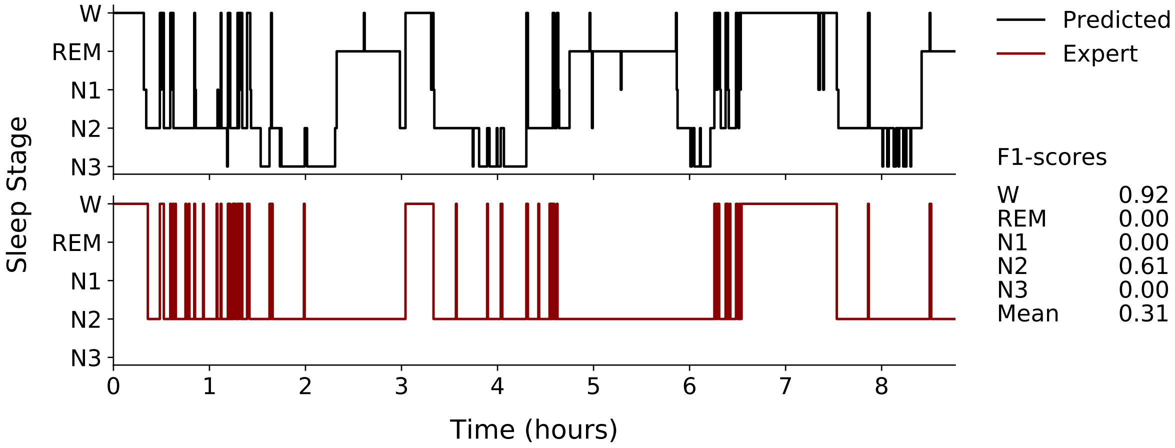

(c) Hypnogram with lowest observed F1-score (record `shhs1-201279`).

Figure 15: Highest, nearest median and lowest scoring (majority voted) hypnograms observed across records in the test-split of dataset `SHHS`. Black hypnograms were predicted by U-Sleep, red hypnograms are human expert annotations. F1-scores for each stage are shown to the right of each set of hypnograms. Each hypnogram displays at most 30 minutes of wake prior to and following the first and last non-wake period, respectively, as determined by the human expert annotations.

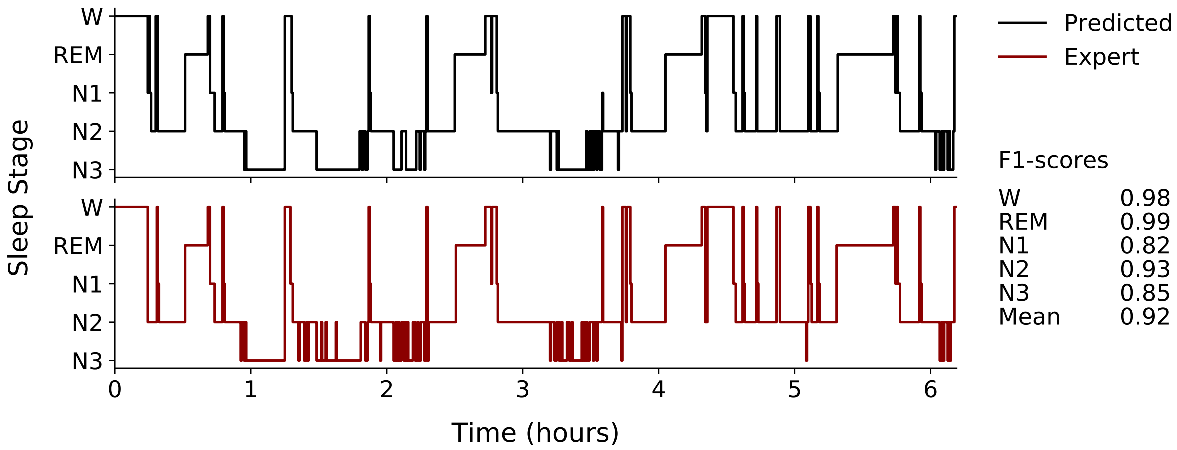

(a) Hypnogram with highest observed F1-score (record `sof-visit-8-10354`).

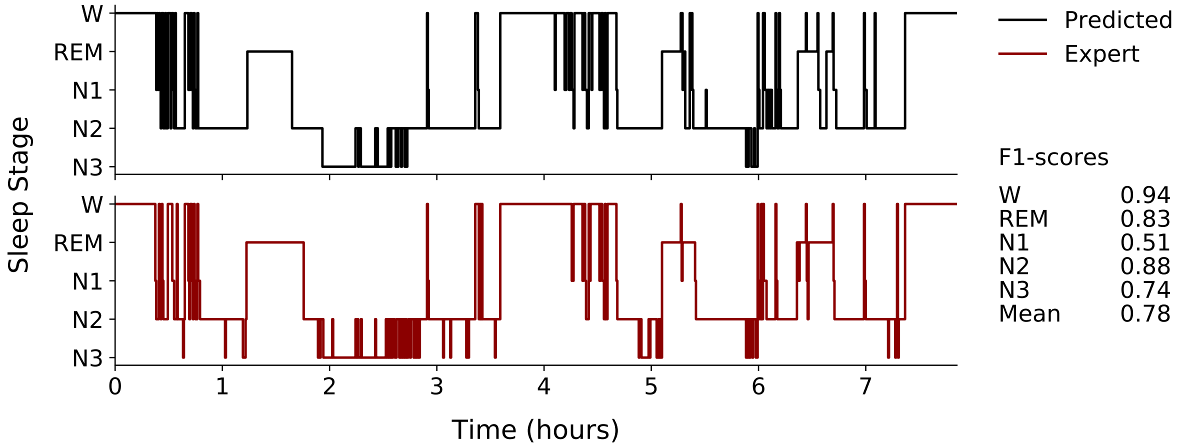

(b) Hypnogram with F1-score nearest dataset median (record `sof-visit-8-09115`).

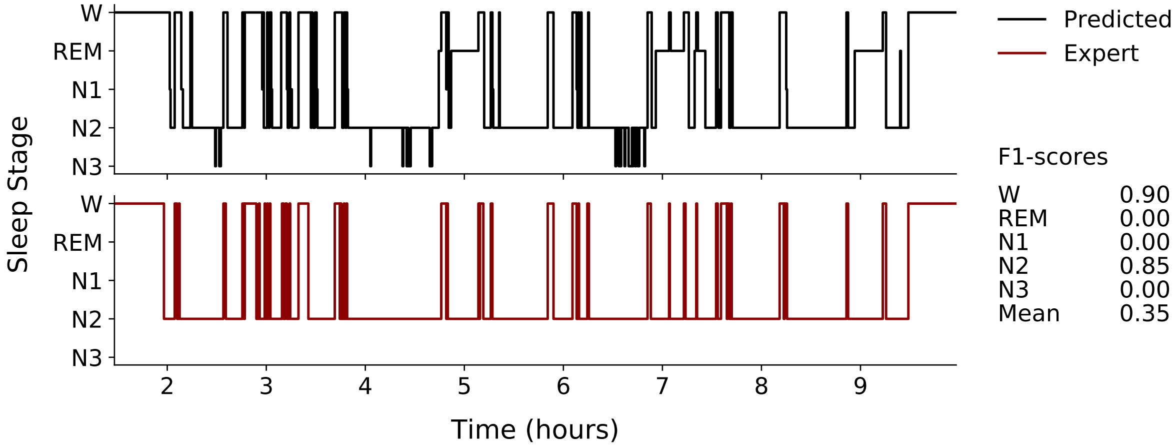

(c) Hypnogram with lowest observed F1-score (record `sof-visit-8-10514`).

Figure 16: Highest, nearest median and lowest scoring (majority voted) hypnograms observed across records in the test-split of dataset `SOF`. Black hypnograms were predicted by U-Sleep, red hypnograms are human expert annotations. F1-scores for each stage are shown to the right of each set of hypnograms. Each hypnogram displays at most 30 minutes of wake prior to and following the first and last non-wake period, respectively, as determined by the human expert annotations.

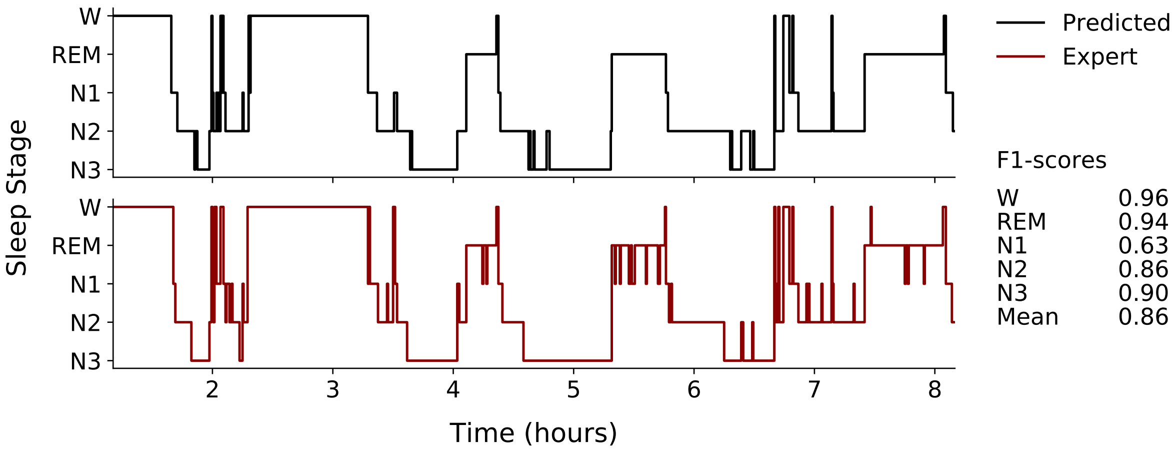

(a) Hypnogram with highest observed F1-score (record `subject_48`).

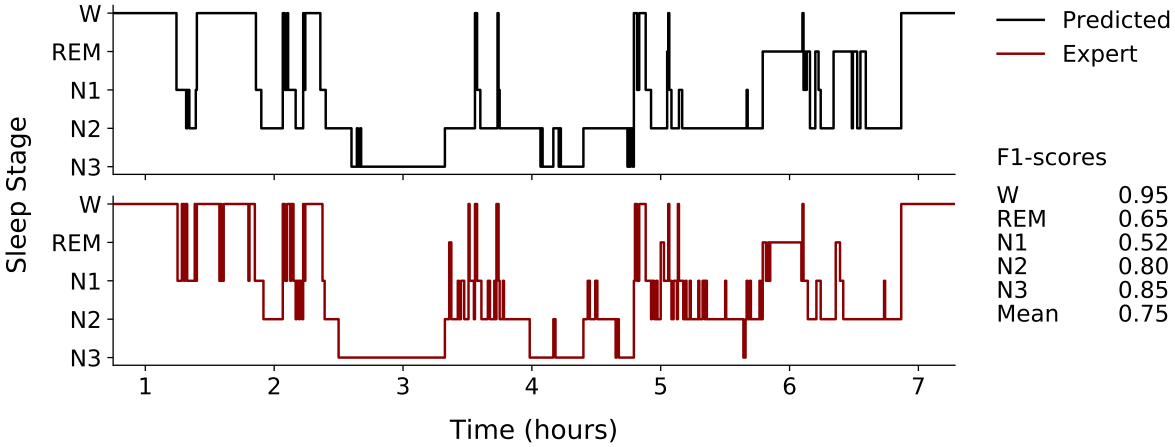

(b) Hypnogram with F1-score nearest dataset median (record `subject_5`).

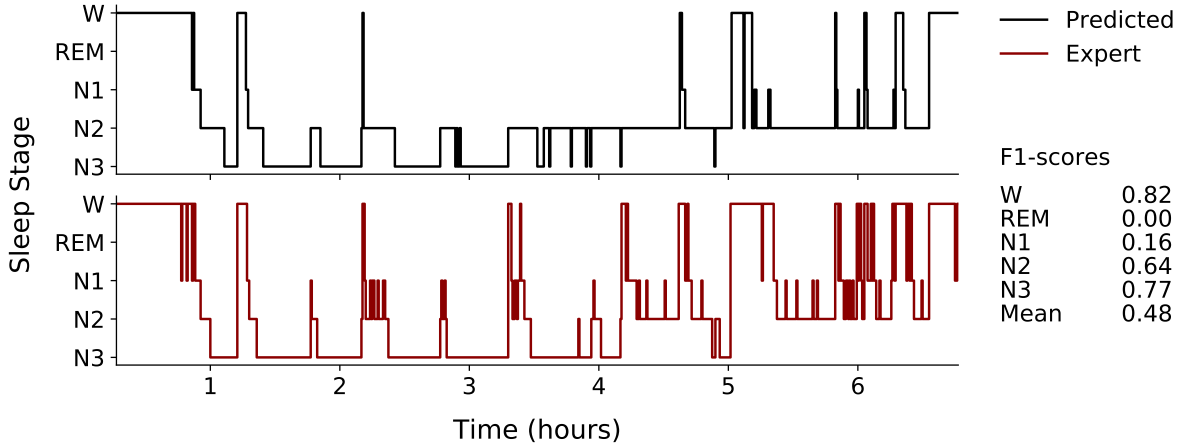

(c) Hypnogram with lowest observed F1-score (record `subject_54`).

Figure 17: Highest, nearest median and lowest scoring (majority voted) hypnograms observed across records in the test-split of dataset `ISRUC-SG1`. Black hypnograms were predicted by U-Sleep, red hypnograms are human expert annotations. F1-scores for each stage are shown to the right of each set of hypnograms. Each hypnogram displays at most 30 minutes of wake prior to and following the first and last non-wake period, respectively, as determined by the human expert annotations.

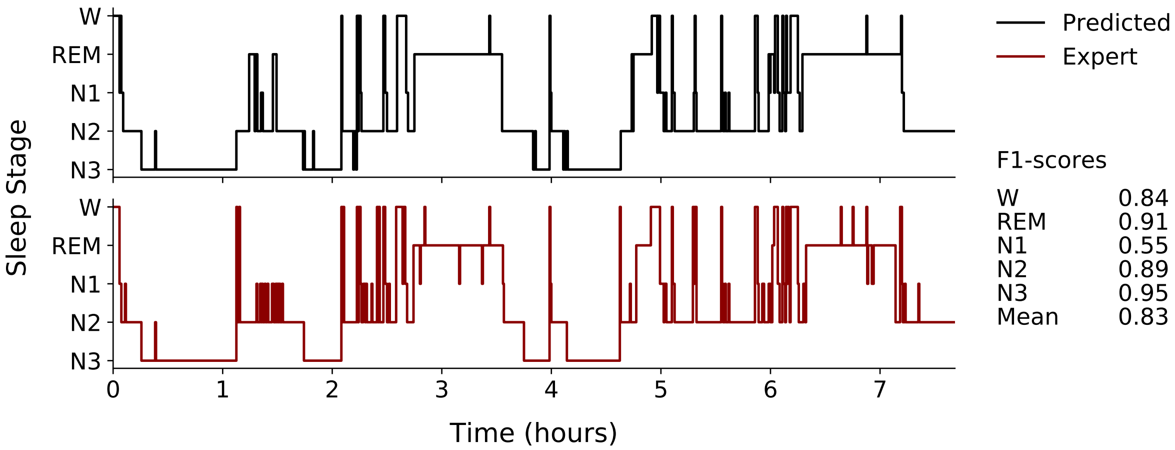

(a) Hypnogram with highest observed F1-score (record `subject_8_visit_2`).

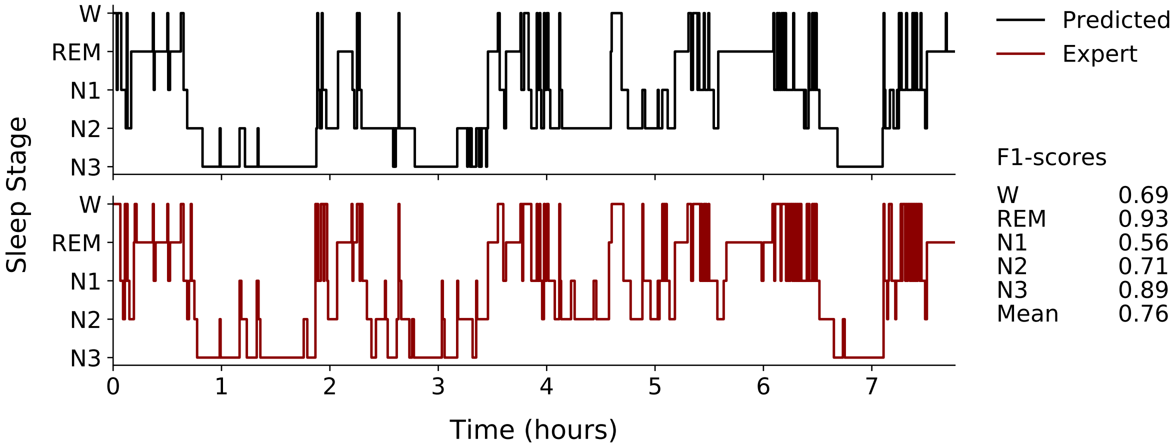

(b) Hypnogram with F1-score nearest dataset median (record `subject_1_visit_1`).

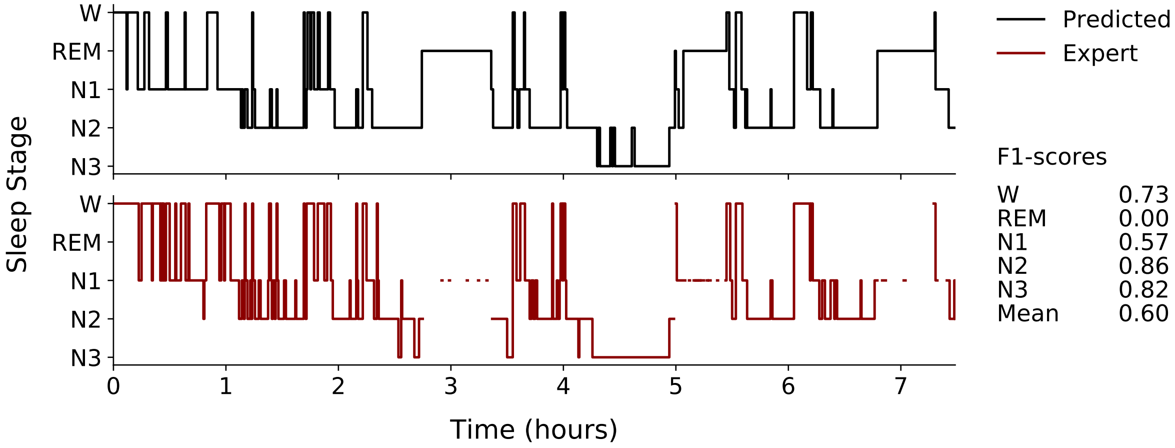

(c) Hypnogram with lowest observed F1-score (record `subject_7_visit_2`).

Figure 18: Highest, nearest median and lowest scoring (majority voted) hypnograms observed across records in the test-split of dataset `ISRUC-SG2`. Black hypnograms were predicted by U-Sleep, red hypnograms are human expert annotations. F1-scores for each stage are shown to the right of each set of hypnograms. Each hypnogram displays at most 30 minutes of wake prior to and following the first and last non-wake period, respectively, as determined by the human expert annotations.

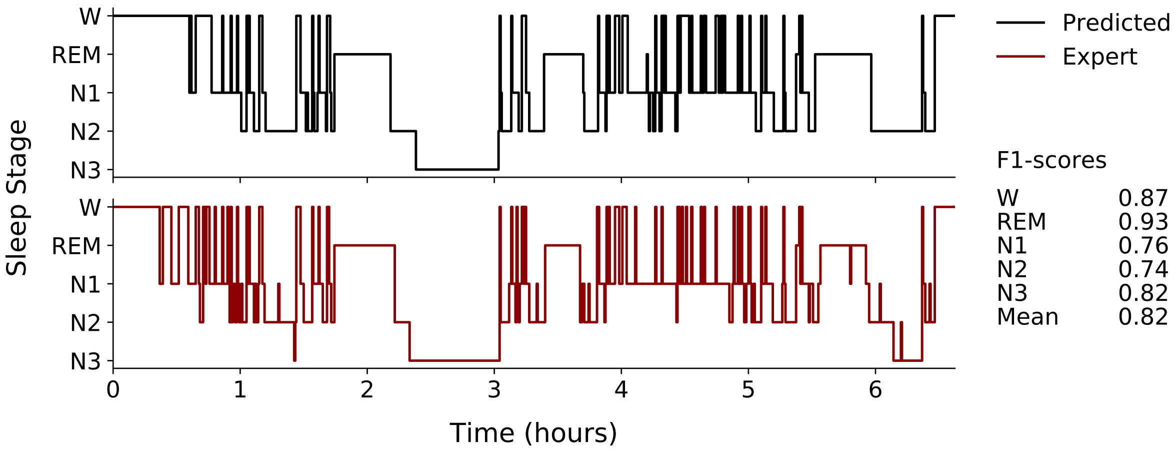

(a) Hypnogram with highest observed F1-score (record `subject_10`).

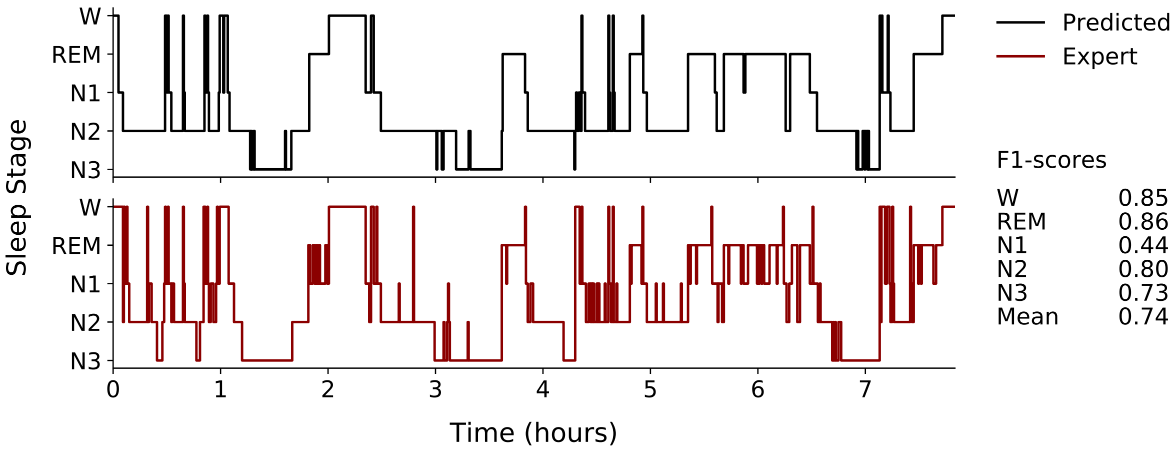

(b) Hypnogram with F1-score nearest dataset median (record `subject_2`).

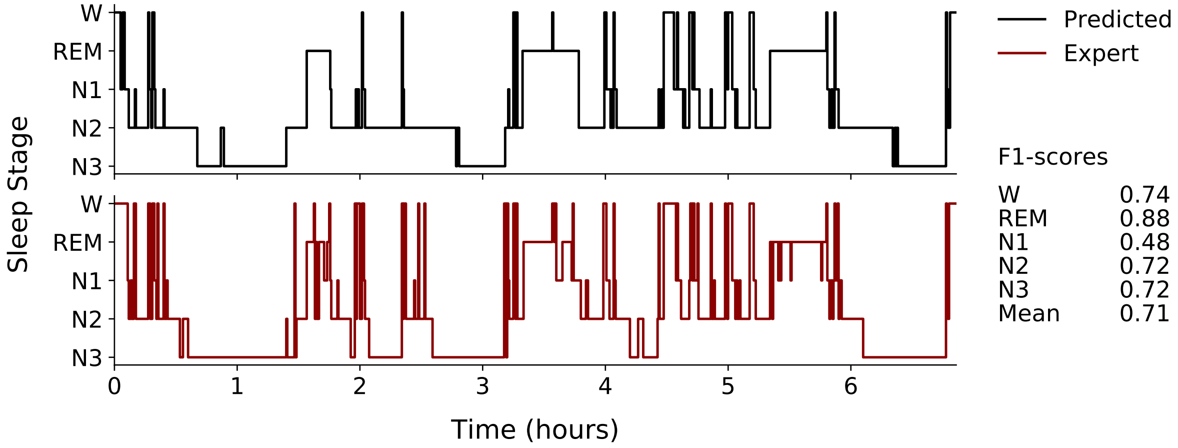

(c) Hypnogram with lowest observed F1-score (record `subject_3`).

Figure 19: Highest, nearest median and lowest scoring (majority voted) hypnograms observed across records in the test-split of dataset `ISRUC-SG3`. Black hypnograms were predicted by U-Sleep, red hypnograms are human expert annotations. F1-scores for each stage are shown to the right of each set of hypnograms. Each hypnogram displays at most 30 minutes of wake prior to and following the first and last non-wake period, respectively, as determined by the human expert annotations.

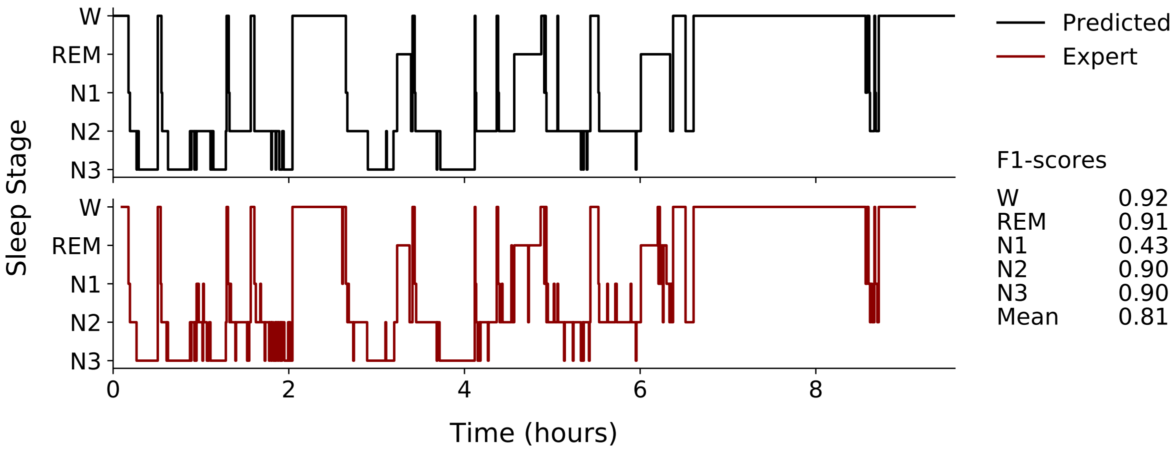

(a) Hypnogram with highest observed F1-score (record `01-01-0016`).

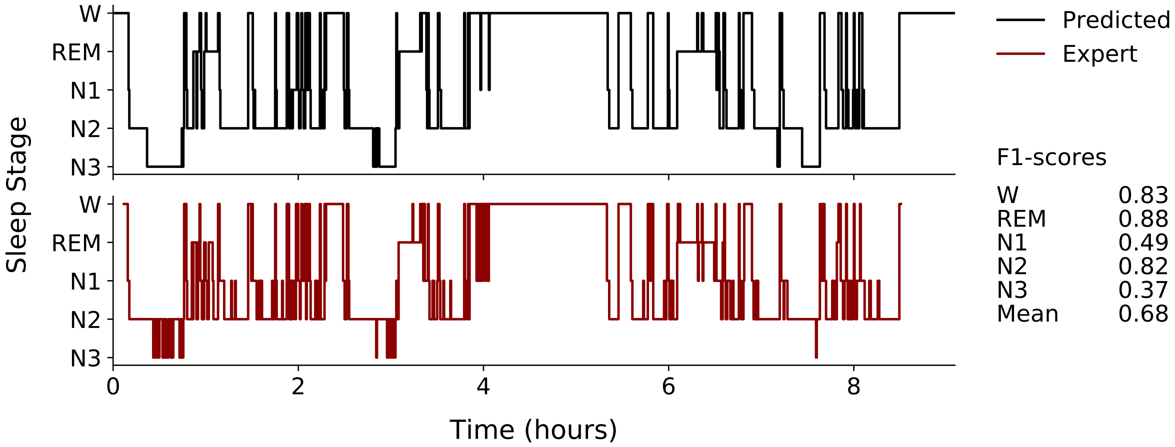

(b) Hypnogram with F1-score nearest dataset median (record `01-01-0047`).

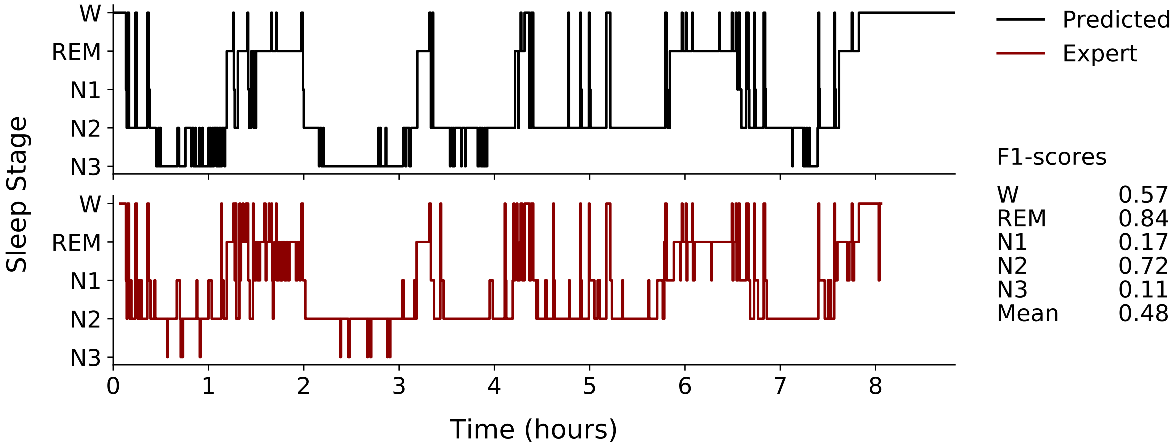

(c) Hypnogram with lowest observed F1-score (record `01-01-0031`).

Figure 20: Highest, nearest median and lowest scoring (majority voted) hypnograms observed across records in the test-split of dataset `MASS-C1`. Black hypnograms were predicted by U-Sleep, red hypnograms are human expert annotations. F1-scores for each stage are shown to the right of each set of hypnograms. Each hypnogram displays at most 30 minutes of wake prior to and following the first and last non-wake period, respectively, as determined by the human expert annotations.

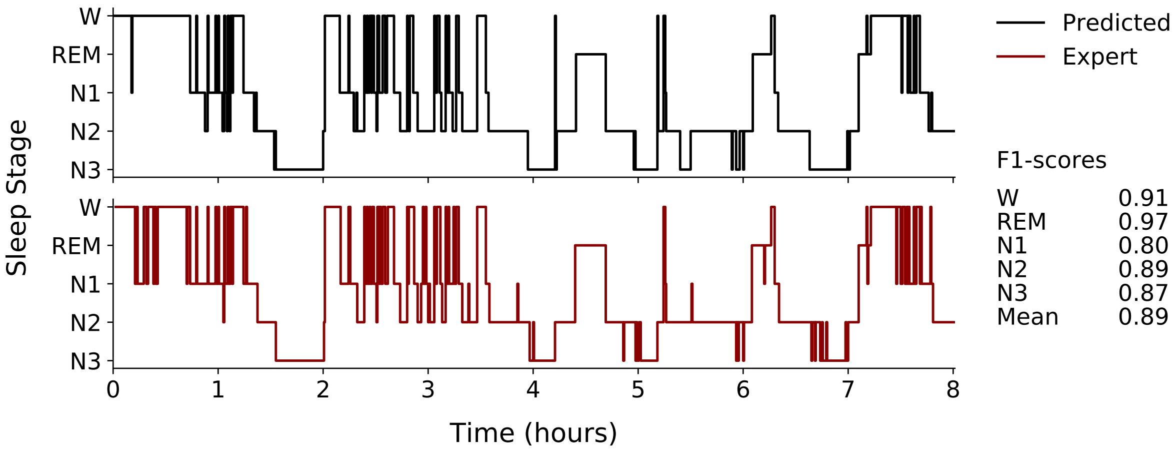

(a) Hypnogram with highest observed F1-score (record `01-03-0035`).

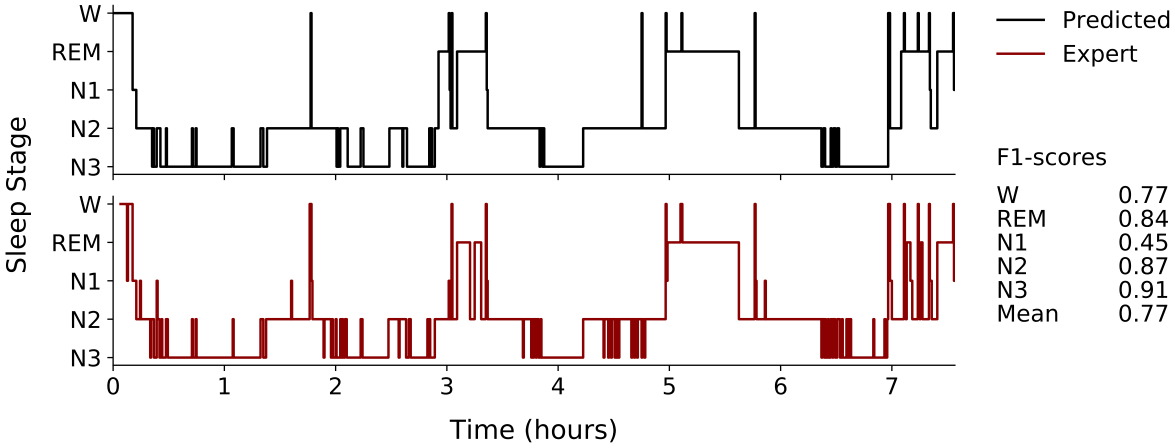

(b) Hypnogram with F1-score nearest dataset median (record `01-03-0008`).

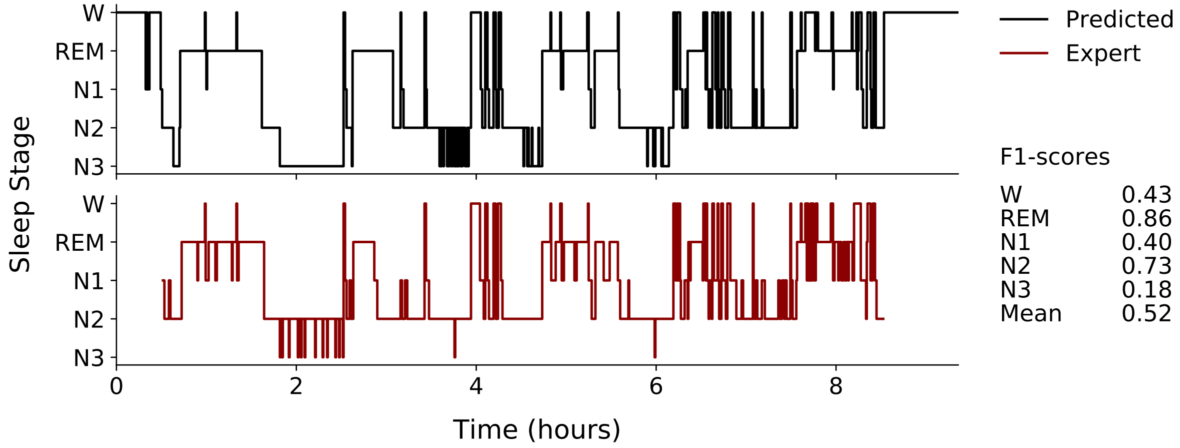

(c) Hypnogram with lowest observed F1-score (record `01-03-0058`).

Figure 21: Highest, nearest median and lowest scoring (majority voted) hypnograms observed across records in the test-split of dataset `MASS-C3`. Black hypnograms were predicted by U-Sleep, red hypnograms are human expert annotations. F1-scores for each stage are shown to the right of each set of hypnograms. Each hypnogram displays at most 30 minutes of wake prior to and following the first and last non-wake period, respectively, as determined by the human expert annotations.

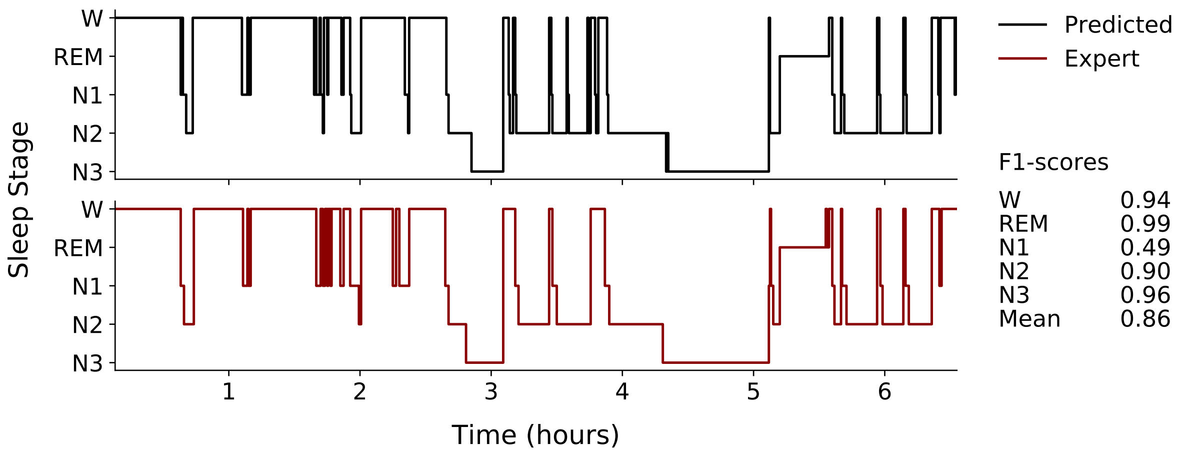

(a) Hypnogram with highest observed F1-score (record `ucddb022`).

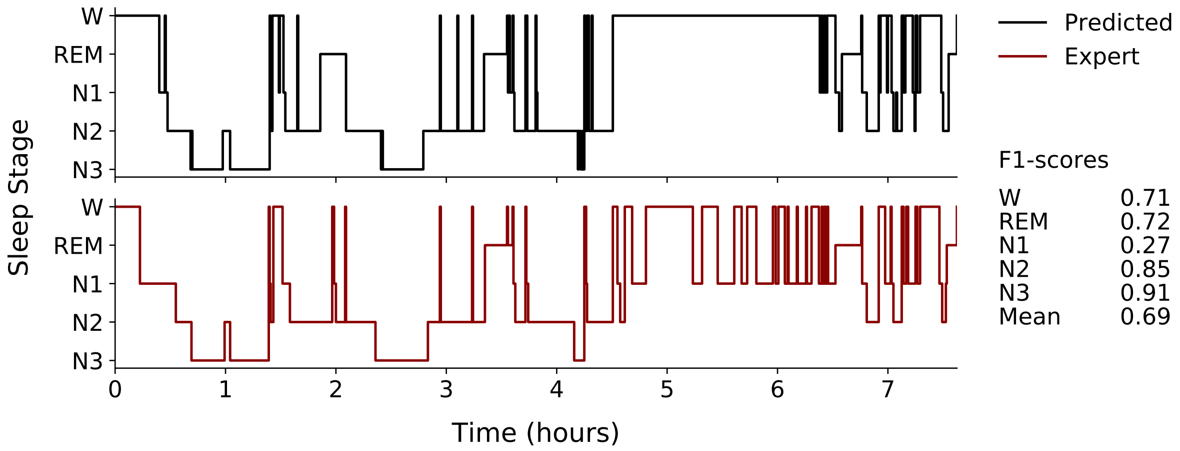

(b) Hypnogram with F1-score nearest dataset median (record `ucddb015`).

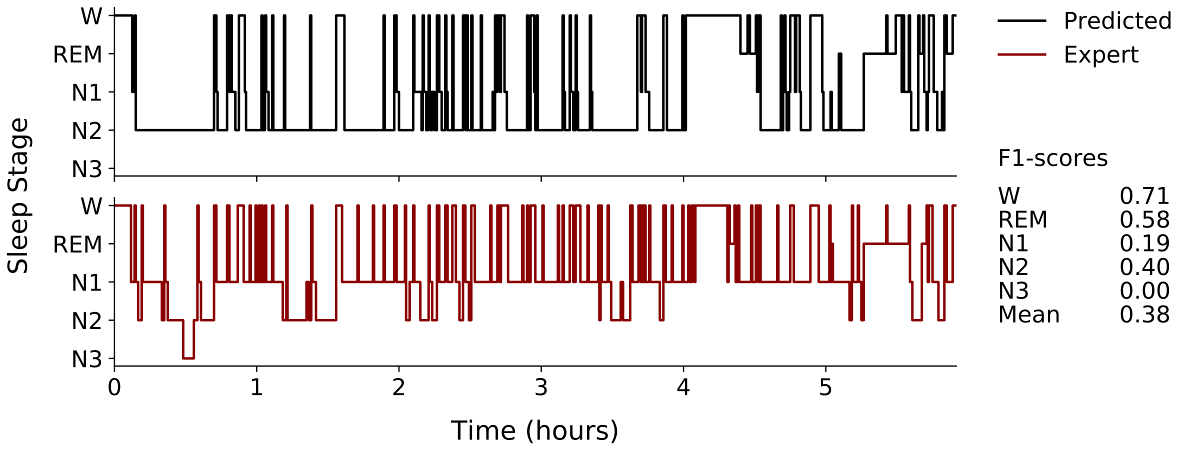

(c) Hypnogram with lowest observed F1-score (record `ucddb025`).

Figure 22: Highest, nearest median and lowest scoring (majority voted) hypnograms observed across records in the test-split of dataset `SVUH`. Black hypnograms were predicted by U-Sleep, red hypnograms are human expert annotations. F1-scores for each stage are shown to the right of each set of hypnograms. Each hypnogram displays at most 30 minutes of wake prior to and following the first and last non-wake period, respectively, as determined by the human expert annotations.

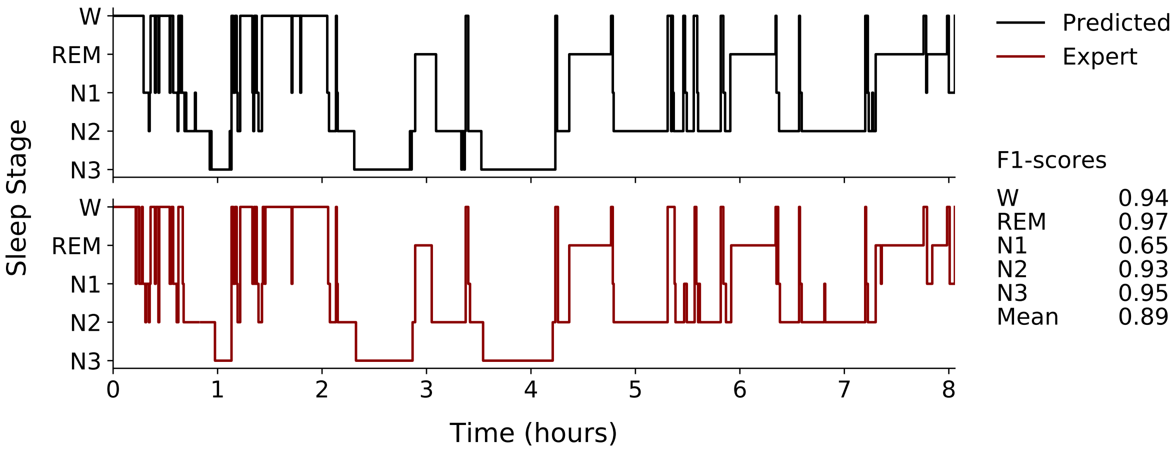

(a) Hypnogram with highest observed F1-score (record `889dcc46-9998-4b54-9c49-f291f153d101`).

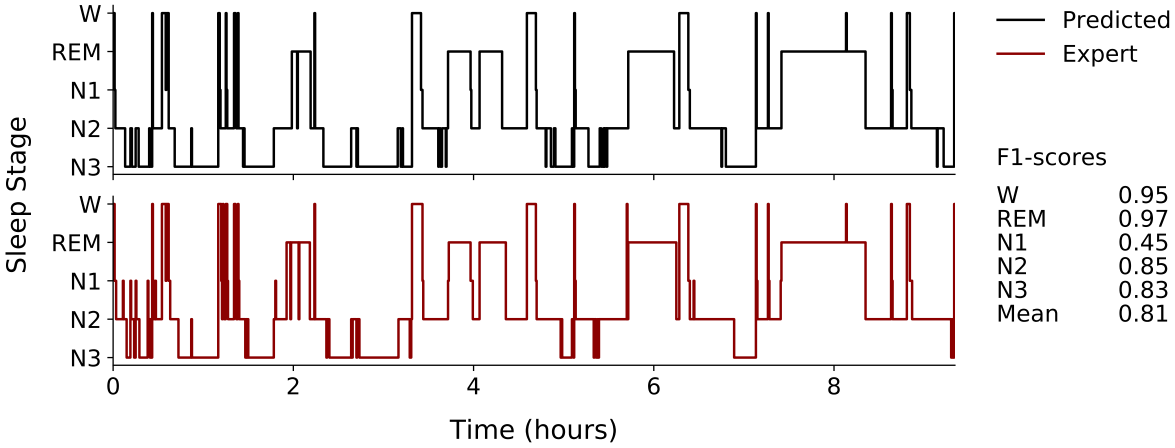

(b) Hypnogram with F1-score nearest dataset median (record `01e60017-d3b5-41cf-bcfb-bde09d46003f`).

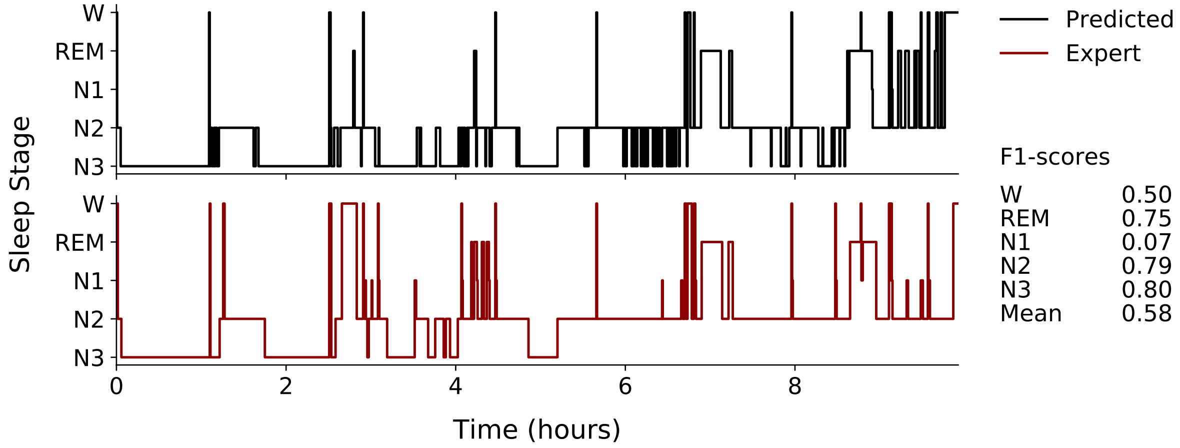

(c) Hypnogram with lowest observed F1-score (record `42f25159-530e-47be-ab07-0895e565ad08`).

Figure 23: Highest, nearest median and lowest scoring (majority voted) hypnograms observed across records in the test-split of dataset `DOD-H`. Black hypnograms were predicted by U-Sleep, red hypnograms are human expert annotations. F1-scores for each stage are shown to the right of each set of hypnograms. Each hypnogram displays at most 30 minutes of wake prior to and following the first and last non-wake period, respectively, as determined by the human expert annotations.

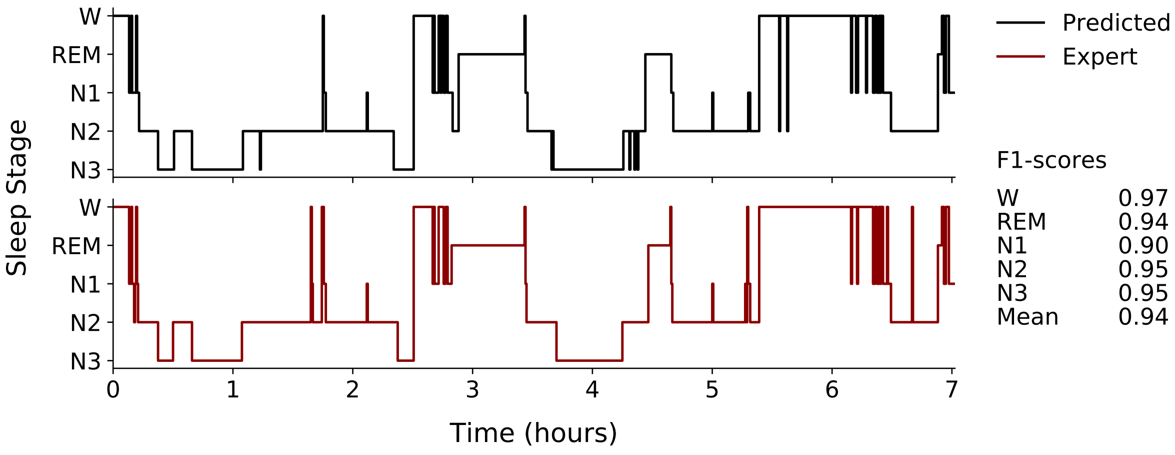

(a) Hypnogram with highest observed F1-score (record `c900fd7f-649d-4ce9-b618-d83c4dea898a`).

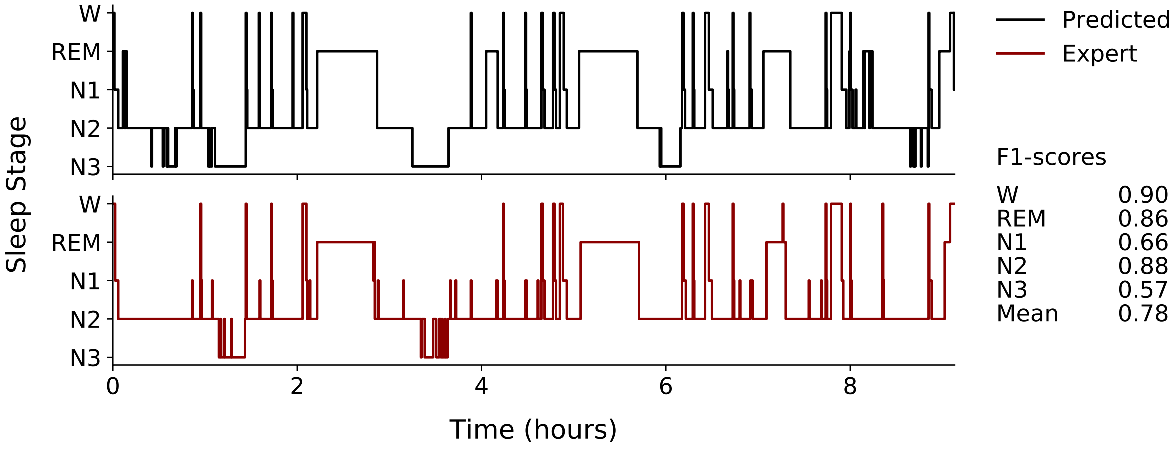

(b) Hypnogram with F1-score nearest dataset median (record `7259faa4-fef7-4d76-834d-a1a5e4a04b85`).

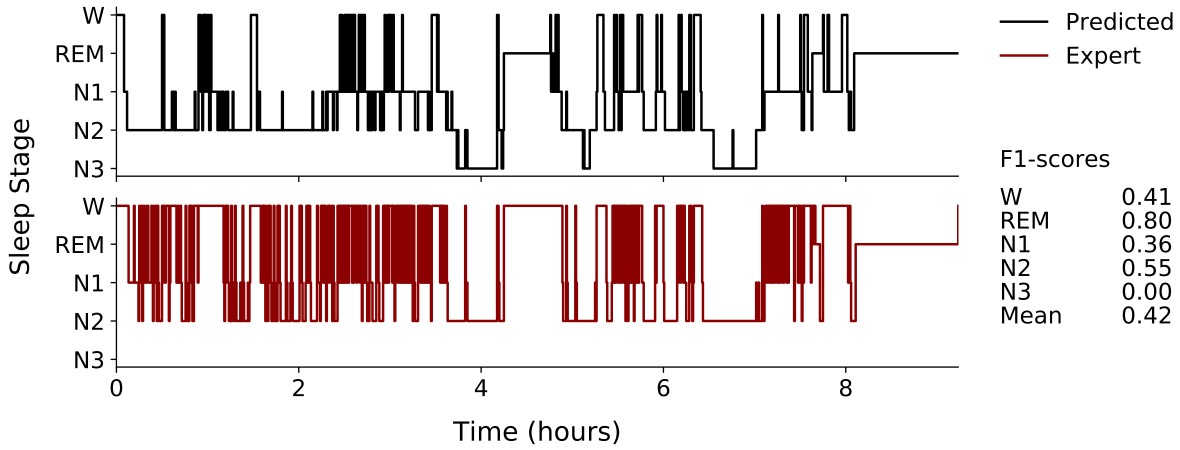

(c) Hypnogram with lowest observed F1-score (record `d203e2a0-b261-4b11-9b76-74709094690d`).

Figure 24: Highest, nearest median and lowest scoring (majority voted) hypnograms observed across records in the test-split of dataset `DOD-O`. Black hypnograms were predicted by U-Sleep, red hypnograms are human expert annotations. F1-scores for each stage are shown to the right of each set of hypnograms. Each hypnogram displays at most 30 minutes of wake prior to and following the first and last non-wake period, respectively, as determined by the human expert annotations.

Table 1: Brief characterization of typical features of the 5 sleep stages as defined by the AASM manual[27].

Name	Encoding	Description
Wake	W	Spans wakefulness to drowsiness. Consists of at least 50% alpha waves (8-13 Hz EEG signals). Rapid and reading eye movements. Eye blinks may occur.
Non-REM 1	N1	Short, light sleep stage comprising about 5%-10% of a night's sleep. Dominated by theta waves (4-7 Hz EEG signals). Slow eye movements in W $\rightarrow$ N1 transition. Some EMG activity, but lower than wake.
Non-REM 2	N2	Comprises 40%-50% of a normal night's sleep. EEG dispalys theta-waves like N1, but intercepted by so-called K-complexes and/or sleep spindles (short bursts of 13-16Hz EEG signal).
Non-REM 3	N3	Comprises about 20%-25% of a typical night's sleep. High amplitude, slow 0.3-3 Hz EEG signals. Low EMG activity.
REM	R	Rapid-eye-movements may occur. Displays both theta waves and alpha (like wake), but typically 1-2 Hz slower. EMG significantly reduced. Dreaming may occur in this stage, which comprises 20%-25% of the night's sleep.

Table 2: U-Sleep model topology for input window size $i = 3840$ (30 seconds of 128 Hz signal), number of input channels $C = 2$, sequence length $T = 35$, number of output classes $K = 5$ and complexity factor scaling value $\alpha = 1.67$. The complexity scaling modifies the filter number in each block or layer as described in the Methods section to a number $c' = \lfloor c \cdot \sqrt{\alpha} \rfloor$ where c is the original number of filters. Note that $T \cdot i = 134400$. Each encoder block performs the following operations: convolution → activation function → batch normalization → (zero-padding if input length is odd) → max pooling (kernel width 2, stride 2). Each encoder block also outputs a residual connection (the output of the layer immediately before max pooling) which is passed to its corresponding decoder block. Each decoder block performs the following operations on its input: nearest-neighbour up-sampling (kernel width 2) → convolution → activation function → batch normalization → (crop if needed to match residual connection input) → concatenation with residual connection → convolution → activation function → batch normalization. The average pooling layer (ID=28) has striding of $i = 3840$.

ID	Layer Type	Output dim	Kernel	Filters	Activation	Pad
-	Input (symbolic)	$T \cdot i \times C$	-	-	-	-
1	Input	134400×2	-	-	-	-
2	Encoder Block	67200×6	9	6	ELU	same
3	Encoder Block	33600×9	9	9	ELU	same
4	Encoder Block	16800×11	9	11	ELU	same
5	Encoder Block	8400×15	9	15	ELU	same
6	Encoder Block	4200×20	9	20	ELU	same
7	Encoder Block	2100×28	9	28	ELU	same
8	Encoder Block	1050×40	9	40	ELU	same
9	Encoder Block	525×55	9	55	ELU	same
10	Encoder Block	263×77	9	77	ELU	same
11	Encoder Block	132×108	9	108	ELU	same
12	Encoder Block	66×152	9	152	ELU	same
13	Encoder Block	33×214	9	214	ELU	same
14	Convolution + Batch Norm.	33×302	9	306	ELU	same
15	Decoder Block (res=13)	66×428	9	214	ELU	same
16	Decoder Block (res=12)	132×304	9	152	ELU	same
17	Decoder Block (res=11)	264×216	9	108	ELU	same
18	Decoder Block (res=10)	526×154	9	77	ELU	same
19	Decoder Block (res=9)	1050×110	9	55	ELU	same
20	Decoder Block (res=8)	2100×80	9	40	ELU	same
21	Decoder Block (res=7)	4200×56	9	28	ELU	same
22	Decoder Block (res=6)	8400×40	9	20	ELU	same
23	Decoder Block (res=5)	16800×30	9	15	ELU	same
24	Decoder Block (res=4)	33600×22	9	11	ELU	same
25	Decoder Block (res=3)	67200×18	9	9	ELU	same
26	Decoder Block (res=2)	134400×12	9	6	ELU	same
27	Convolution	134400×6	1	6	TanH	same
28	Average Pooling	35×6	3840	-	-	valid
29	Convolution	35×5	1	5	ELU	same
30	Convolution	35×5	1	5	Softmax	same

Trainable parameters: $3,114,337$

Table 3: U-Sleep Model, Optimization and Pre-Processing Hyperparameters.

Parameter	Value	Notes
Optimizer	Adam	See[28].
Learning rate -	10^{-7}	
β_1 -	0.9	
β_2 -	0.999	
ϵ -	$1 \cdot 10^{-8}$	
Loss function	Cross-entropy	
Regularization -	None	
Class balancing -	None	
Base Topology	1D U-Net	The input dimensionality is the number. of data points in a single PSG segment (one segment is 30 seconds in typical sleep staging, giving input dimensionality 3840 for sample rate $S = 128$). T is the number of contiguous segments the model operates on at once. T may be dynamically adjusted. Zero-padding is needed to decode to dimensions equal to the input[29–32]. ELU=Exponential Linear Units[33].
Input dim. -	3840	
Window size (T) -	35	
Depth -	12	
Up-sampling -	Nearest neighbour	
Activations -	ELU	
Conv. kernel size -	9	
Conv. kernel dilation size -	9	
Max-pool kernel size -	2	
Padding -	True ('same')	
Batch normalization -	True	
Parameters -	$\approx 3.12 \cdot 10^6$	
Pre-processing	Robust scaling	Record- and channel-wise transformation to distribution of median 0 and IQR 1. Re-sampling uses polyphase filtering (implementation: `scipy.signal.resample_poly`[34]). Clamping of absolute values deviating from the median by more than 20 times the IQR of the channel.
Post-processing	None	
Re-sampling (S)	128 Hz	
Batch size (B)	64	For element in a batch, a class from the label set {W, N1, N2, N3, R} is determined by uniform sampling. A random PSG record of this class is sampled, from which the input window is sampled randomly so that the selected class is in the window.
Class sampling prob. -	Uniform	
Training epochs	∞	Training continues until 100 consecutive epochs without validation performance improvements. 443 steps amounts to roughly 10^6 30-second segments (or labels, equivalently).
Steps per epoch	443	
Early stopping criteria	Validation F1	Mean per-class F1 scores computed over random subsets of up to 20 validation records from each dataset.
Model selection criteria	Validation F1	

Table 4: ABC - Test data - Channel-Wise F1/dice scores (computed across subjects)

	Records	Wake	N1	N2	N3	REM	mean
F4-M1+E2-M1	20	0.81	0.49	0.78	0.60	0.79	0.69
F3-M2+E2-M1	20	0.83	0.48	0.78	0.53	0.85	0.70
C3-M2+E2-M1	20	0.84	0.50	0.81	0.62	0.86	0.73
O1-M2+E2-M1	20	0.84	0.51	0.81	0.63	0.88	0.73
F3-M2+E1-M2	20	0.85	0.48	0.83	0.71	0.90	0.75
O2-M1+E2-M1	20	0.86	0.51	0.83	0.71	0.88	0.76
O1-M2+E1-M2	20	0.85	0.48	0.84	0.73	0.90	0.76
C4-M1+E2-M1	20	0.85	0.51	0.83	0.73	0.87	0.76
F4-M1+E1-M2	20	0.86	0.50	0.83	0.71	0.89	0.76
C3-M2+E1-M2	20	0.86	0.53	0.84	0.75	0.91	0.78
O2-M1+E1-M2	20	0.87	0.52	0.84	0.76	0.90	0.78
C4-M1+E1-M2	20	0.87	0.53	0.84	0.76	0.90	0.78
Mean		0.85	0.50	0.82	0.69	0.88	0.75
Standard deviation		0	0.02	0.02	0.07	0.03	0.03
Majority vote	20	0.87	0.53	0.84	0.72	0.90	0.77

Table 5: CCSHS - Test data - Channel-Wise F1/dice scores (computed across subjects)

	Records	Wake	N1	N2	N3	REM	mean
C4-A1+ROC-A1	78	0.91	0.57	0.90	0.86	0.90	0.83
C4-A1+LOC-A2	78	0.92	0.57	0.90	0.86	0.91	0.83
C3-A2+ROC-A1	78	0.92	0.58	0.90	0.87	0.91	0.84
C3-A2+LOC-A2	78	0.92	0.59	0.90	0.87	0.91	0.84
Mean		0.92	0.58	0.90	0.86	0.91	0.83
Standard deviation		0	0.01	0.00	0.00	0.00	0.01
Majority vote	78	0.93	0.63	0.91	0.88	0.93	0.85

Table 6: CFS - Test data - Channel-Wise F1/dice scores (computed across subjects)

	Records	Wake	N1	N2	N3	REM	mean
C3-A2+ROC-A1	92	0.93	0.48	0.87	0.83	0.91	0.80
C3-A2+LOC-A2	92	0.93	0.50	0.87	0.82	0.90	0.81
C4-A1+LOC-A2	92	0.93	0.49	0.88	0.84	0.90	0.81
C4-A1+ROC-A1	92	0.93	0.51	0.88	0.84	0.91	0.81
Mean		0.93	0.50	0.88	0.83	0.91	0.81
Standard deviation		0	0.01	0.00	0.01	0.00	0.00
Majority vote	92	0.93	0.52	0.89	0.84	0.91	0.82

Table 7: CHAT - Test data - Channel-Wise F1/dice scores (computed across subjects)

	Records	Wake	N1	N2	N3	REM	mean
O2-M1+E1-M2	128	0.91	0.58	0.84	0.89	0.86	0.81
T3-M2+E1-M2	128	0.90	0.60	0.84	0.89	0.87	0.82
T4-M1+E1-M2	128	0.91	0.60	0.84	0.89	0.87	0.82
C4-M1+E1-M2	128	0.92	0.60	0.84	0.89	0.87	0.82
F3-M2+E1-M2	128	0.91	0.60	0.85	0.89	0.88	0.82
C3-M2+E2-M1	128	0.91	0.60	0.85	0.89	0.88	0.83
T3-M2+E2-M1	128	0.90	0.61	0.85	0.89	0.88	0.83
O1-M2+E1-M2	128	0.91	0.60	0.85	0.89	0.88	0.83
T4-M1+E2-M1	128	0.91	0.61	0.85	0.89	0.89	0.83
C3-M2+E1-M2	128	0.91	0.60	0.85	0.90	0.88	0.83
O2-M1+E2-M1	128	0.92	0.60	0.85	0.89	0.88	0.83
F4-M1+E1-M2	128	0.92	0.60	0.85	0.89	0.89	0.83
O1-M2+E2-M1	128	0.91	0.61	0.86	0.89	0.89	0.83
F3-M2+E2-M1	128	0.92	0.62	0.86	0.89	0.89	0.84
F4-M1+E2-M1	128	0.92	0.61	0.86	0.89	0.90	0.84
C4-M1+E2-M1	128	0.93	0.62	0.86	0.89	0.89	0.84
Mean		0.91	0.60	0.85	0.89	0.88	0.83
Standard deviation		0	0.01	0.01	0.00	0.01	0.01
Majority vote	128	0.93	0.64	0.87	0.90	0.90	0.85

Table 8: DCSM - Test data - Channel-Wise F1/dice scores (computed across subjects)

	Records	Wake	N1	N2	N3	REM	mean
O2-M1+E1-M2	39	0.96	0.47	0.82	0.78	0.85	0.78
O1-M2+E1-M2	39	0.96	0.47	0.83	0.79	0.86	0.78
F3-M2+E1-M2	39	0.97	0.44	0.84	0.81	0.87	0.79
C3-M2+E1-M2	39	0.96	0.45	0.84	0.81	0.87	0.79
O2-M1+E2-M2	39	0.97	0.47	0.84	0.81	0.87	0.79
F4-M1+E1-M2	39	0.97	0.46	0.84	0.80	0.89	0.79
O1-M2+E2-M2	39	0.97	0.48	0.84	0.81	0.87	0.79
F3-M2+E2-M2	39	0.97	0.45	0.84	0.82	0.89	0.79
C4-M1+E1-M2	39	0.97	0.46	0.85	0.80	0.90	0.79
F4-M1+E2-M2	39	0.97	0.46	0.85	0.82	0.88	0.80
C3-M2+E2-M2	39	0.97	0.47	0.85	0.82	0.89	0.80
C4-M1+E2-M2	39	0.97	0.46	0.85	0.82	0.90	0.80
Mean		0.97	0.46	0.84	0.81	0.88	0.79
Standard deviation		0	0.01	0.01	0.01	0.01	0.01
Majority vote	39	0.97	0.48	0.86	0.83	0.89	0.81

Table 9: HPAP - Test data - Channel-Wise F1/dice scores (computed across subjects)

	Records	Wake	N1	N2	N3	REM	mean
O2-M1+E1	34	0.89	0.44	0.80	0.72	0.86	0.74
C4-M1+E1	34	0.89	0.41	0.80	0.73	0.89	0.75
F4-M1+E2-M1	32	0.89	0.42	0.82	0.74	0.88	0.75
O2-M1+E2-M1	32	0.90	0.43	0.81	0.73	0.88	0.75
F4-M1+E1	34	0.87	0.47	0.80	0.72	0.88	0.75
C3-M2+E1-M2	32	0.90	0.48	0.82	0.71	0.85	0.75
C4-M1+E2-M1	32	0.90	0.43	0.82	0.73	0.88	0.75
C3-M2+E1	34	0.89	0.47	0.82	0.71	0.86	0.75
O2-M1+E2	34	0.89	0.45	0.81	0.75	0.87	0.75
F3-M2+E1	34	0.89	0.46	0.82	0.74	0.87	0.76
C3-M2+E2-M1	32	0.89	0.48	0.83	0.72	0.87	0.76
O1-M2+E1	34	0.89	0.48	0.81	0.73	0.88	0.76
F3-M2+E1-M2	32	0.90	0.47	0.82	0.74	0.87	0.76
F4-M1+E2	34	0.87	0.47	0.81	0.76	0.89	0.76
C4-M1+E2	34	0.89	0.44	0.82	0.75	0.89	0.76
C3-M2+E2	34	0.89	0.47	0.83	0.74	0.86	0.76
F4-M1+E1-M2	32	0.89	0.47	0.82	0.73	0.89	0.76
C4-M1+E1-M2	32	0.90	0.47	0.82	0.73	0.88	0.76
O1-M2+E2-M1	32	0.90	0.47	0.83	0.73	0.88	0.76
F3-M2+E2-M1	32	0.89	0.47	0.83	0.75	0.88	0.76
O2-M1+E1-M2	32	0.90	0.49	0.82	0.73	0.88	0.76
F3-M2+E2	34	0.89	0.47	0.83	0.76	0.87	0.76
O1-M2+E1-M2	32	0.91	0.48	0.83	0.74	0.88	0.77
O1-M2+E2	34	0.89	0.49	0.82	0.76	0.89	0.77
Mean		0.89	0.46	0.82	0.74	0.88	0.76
Standard deviation		0	0.02	0.01	0.01	0.01	0.01
Majority vote	36	0.91	0.48	0.84	0.78	0.90	0.78

Table 10: MESA - Test data - Channel-Wise F1/dice scores (computed across subjects)

	Records	Wake	N1	N2	N3	REM	mean
Fz-Cz+E2-FPz	100	0.88	0.50	0.83	0.60	0.81	0.72
Fz-Cz+E1-FPz	100	0.90	0.51	0.85	0.66	0.85	0.75
C4-M1+E1-FPz	100	0.91	0.56	0.85	0.62	0.89	0.77
C4-M1+E2-FPz	100	0.91	0.57	0.85	0.65	0.88	0.77
Cz-Oz+E2-FPz	100	0.91	0.58	0.85	0.65	0.88	0.77
Cz-Oz+E1-FPz	100	0.92	0.56	0.86	0.63	0.90	0.77
Mean		0.90	0.55	0.85	0.63	0.87	0.76
Standard deviation		0	0.03	0.01	0.02	0.03	0.02
Majority vote	100	0.92	0.59	0.87	0.65	0.90	0.79

Table 11: MROS - Test data - Channel-Wise F1/dice scores (computed across subjects)

	Records	Wake	N1	N2	N3	REM	mean
C4-M1+E2-M1	134	0.92	0.44	0.86	0.63	0.87	0.75
C4-M1+E1-M2	134	0.92	0.42	0.86	0.66	0.88	0.75
C3-M2+E1-M2	134	0.92	0.43	0.86	0.68	0.87	0.75
C3-M2+E2-M1	134	0.93	0.45	0.86	0.68	0.86	0.76
Mean		0.92	0.43	0.86	0.66	0.87	0.75
Standard deviation		0	0.01	0.00	0.02	0.01	0.00
Majority vote	134	0.93	0.46	0.87	0.68	0.88	0.77

Table 12: PHYS - Test data - Channel-Wise F1/dice scores (computed across subjects)

	Records	Wake	N1	N2	N3	REM	mean
O1-M2+E1-M2	100	0.84	0.59	0.82	0.78	0.86	0.78
O2-M1+E1-M2	100	0.84	0.59	0.82	0.79	0.86	0.78
C3-M2+E1-M2	100	0.83	0.59	0.83	0.79	0.87	0.78
F3-M2+E1-M2	100	0.83	0.58	0.83	0.80	0.87	0.78
C4-M1+E1-M2	100	0.83	0.59	0.84	0.80	0.87	0.79
F4-M1+E1-M2	100	0.83	0.59	0.84	0.81	0.87	0.79
Mean		0.83	0.59	0.83	0.79	0.86	0.78
Standard deviation		0	0.00	0.01	0.01	0.01	0.00
Majority vote	100	0.84	0.60	0.84	0.81	0.87	0.79

Table 13: SEDF-SC - Test data - Channel-Wise F1/dice scores (computed across subjects)

	Records	Wake	N1	N2	N3	REM	mean
Pz-Oz+EOG	23	0.92	0.53	0.83	0.68	0.84	0.76
Fpz-Cz+EOG	23	0.92	0.54	0.86	0.71	0.88	0.78
Mean		0.92	0.54	0.84	0.69	0.86	0.77
Standard deviation		0	0.01	0.01	0.02	0.02	0.01
Majority vote	23	0.93	0.57	0.86	0.71	0.88	0.79

Table 14: SEDF-ST - Test data - Channel-Wise F1/dice scores (computed across subjects)

	Records	Wake	N1	N2	N3	REM	mean
Fpz-Cz+EOG	8	0.80	0.55	0.87	0.64	0.91	0.76
Pz-Oz+EOG	8	0.79	0.60	0.89	0.63	0.90	0.76
Mean		0.79	0.57	0.88	0.63	0.91	0.76
Standard deviation		0	0.03	0.01	0.01	0.01	0.00
Majority vote	8	0.80	0.58	0.88	0.64	0.91	0.76

Table 15: SHHS - Test data - Channel-Wise F1/dice scores (computed across subjects)

	Records	Wake	N1	N2	N3	REM	mean
C4-A1+EOG(R)-PG1	140	0.91	0.45	0.86	0.74	0.91	0.77
C3-A2+EOG(L)-PG1	135	0.91	0.49	0.86	0.75	0.89	0.78
C4-A1+EOG(L)-PG1	140	0.92	0.48	0.87	0.76	0.91	0.79
C3-A2+EOG(R)-PG1	135	0.92	0.50	0.87	0.76	0.90	0.79
Mean		0.92	0.48	0.86	0.75	0.90	0.78
Standard deviation		0	0.02	0.00	0.01	0.01	0.01
Majority vote	140	0.93	0.51	0.87	0.76	0.92	0.80

Table 16: SOF - Test data - Channel-Wise F1/dice scores (computed across subjects)

	Records	Wake	N1	N2	N3	REM	mean
C4-A1+ROC-A1	68	0.93	0.45	0.84	0.70	0.89	0.76
C3-A2+LOC-A2	68	0.91	0.37	0.84	0.78	0.91	0.76
C3-A2+ROC-A1	68	0.92	0.42	0.84	0.74	0.91	0.77
C4-A1+LOC-A2	68	0.93	0.42	0.85	0.76	0.91	0.77
Mean		0.92	0.42	0.84	0.74	0.91	0.77
Standard deviation		0	0.03	0.00	0.03	0.01	0.00
Majority vote	68	0.93	0.45	0.86	0.77	0.92	0.78

Table 17: ISRUC-SG1 - Test data - Channel-Wise F1/dice scores (computed across subjects)

	Records	Wake	N1	N2	N3	REM	mean
C3-M2+E2-M1	100	0.87	0.50	0.74	0.69	0.82	0.72
C3-M2+E1-M2	100	0.84	0.49	0.77	0.75	0.85	0.74
F3-M2+E2-M1	99	0.88	0.50	0.77	0.72	0.85	0.75
F4-M1+E2-M1	99	0.89	0.50	0.77	0.73	0.85	0.75
O1-M2+E1-M2	100	0.86	0.48	0.78	0.76	0.87	0.75
F4-M1+E1-M2	99	0.86	0.49	0.78	0.76	0.88	0.75
O1-M2+E2-M1	100	0.88	0.49	0.78	0.76	0.86	0.75
O2-M1+E2-M1	100	0.87	0.51	0.77	0.77	0.86	0.76
C4-M1+E2-M1	100	0.88	0.50	0.78	0.77	0.86	0.76
O2-M1+E1-M2	100	0.86	0.48	0.78	0.80	0.87	0.76
F3-M2+E1-M2	99	0.86	0.50	0.79	0.79	0.87	0.76
C4-M1+E1-M2	100	0.86	0.48	0.79	0.81	0.88	0.76
Mean		0.87	0.49	0.77	0.76	0.86	0.75
Standard deviation		0	0.01	0.01	0.03	0.01	0.01
Majority vote	100	0.89	0.52	0.79	0.77	0.88	0.77

Table 18: ISRUC-SG2 - Test data - Channel-Wise F1/dice scores (computed across subjects)

	Records	Wake	N1	N2	N3	REM	mean
C4-M1+E2-M1	16	0.80	0.48	0.76	0.80	0.84	0.74
C3-M2+E2-M1	16	0.79	0.46	0.77	0.81	0.85	0.74
O1-M2+E2-M1	16	0.80	0.48	0.76	0.82	0.85	0.74
O2-M1+E2-M1	16	0.80	0.48	0.77	0.81	0.85	0.74
F3-M2+E2-M1	16	0.81	0.49	0.77	0.82	0.83	0.74
C4-M1+E1-M2	16	0.84	0.47	0.77	0.82	0.83	0.75
F4-M1+E2-M1	16	0.81	0.49	0.78	0.82	0.86	0.75
F3-M2+E1-M2	16	0.85	0.48	0.78	0.83	0.82	0.75
C3-M2+E1-M2	16	0.85	0.47	0.78	0.83	0.85	0.76
O1-M2+E1-M2	16	0.84	0.47	0.78	0.84	0.86	0.76
O2-M1+E1-M2	16	0.85	0.49	0.78	0.83	0.85	0.76
F4-M1+E1-M2	16	0.87	0.48	0.78	0.83	0.85	0.76
Mean		0.83	0.48	0.77	0.82	0.84	0.75
Standard deviation		0	0.01	0.01	0.01	0.01	0.01
Majority vote	16	0.85	0.49	0.78	0.83	0.86	0.76

Table 19: ISRUC-SG3 - Test data - Channel-Wise F1/dice scores (computed across subjects)

	Records	Wake	N1	N2	N3	REM	mean
O1-M2+E2-M1	10	0.84	0.54	0.77	0.72	0.87	0.75
C3-M2+E2-M1	10	0.86	0.54	0.77	0.71	0.86	0.75
F4-M1+E1-M2	10	0.91	0.54	0.76	0.69	0.84	0.75
F3-M2+E2-M1	10	0.85	0.53	0.77	0.74	0.85	0.75
O2-M1+E2-M1	10	0.85	0.54	0.77	0.75	0.85	0.75
O1-M2+E1-M2	10	0.91	0.54	0.76	0.70	0.85	0.75
C4-M1+E1-M2	10	0.91	0.49	0.78	0.76	0.84	0.75
C4-M1+E2-M1	10	0.85	0.53	0.78	0.75	0.85	0.75
F4-M1+E2-M1	10	0.86	0.55	0.76	0.72	0.87	0.75
C3-M2+E1-M2	10	0.92	0.53	0.77	0.73	0.85	0.76
O2-M1+E1-M2	10	0.90	0.53	0.79	0.78	0.83	0.76
F3-M2+E1-M2	10	0.92	0.52	0.79	0.78	0.85	0.77
Mean		0.88	0.53	0.77	0.74	0.85	0.75
Standard deviation		0	0.02	0.01	0.03	0.01	0.01
Majority vote	10	0.90	0.55	0.78	0.74	0.85	0.77

Table 20: MASS-C1 (part 1/2) - Test data - Channel-Wise F1/dice scores (computed across subjects). This table displays all MASS-C1 evaluations on EOG(R) channel combinations. Please refer to Table 21 for MASS-C1 evaluations with EOG(L) channels and for mean, standard deviation and majority vote summaries across both EOG(L) and EOG(R) channel combinations.

	Records	Wake	N1	N2	N3	REM	mean
P3-LER+EOG(R)	6	0.93	0.32	0.66	0.39	0.90	0.64
T4-LER+EOG(R)	6	0.93	0.34	0.68	0.39	0.91	0.65
F8-LER+EOG(R)	6	0.91	0.36	0.72	0.46	0.85	0.66
O2-LER+EOG(R)	6	0.92	0.38	0.72	0.44	0.85	0.66
T6-LER+EOG(R)	6	0.93	0.35	0.71	0.44	0.90	0.66
C4-LER+EOG(R)	6	0.90	0.39	0.73	0.47	0.85	0.67
T3-LER+EOG(R)	6	0.92	0.35	0.73	0.47	0.87	0.67
F4-LER+EOG(R)	6	0.93	0.34	0.72	0.46	0.90	0.67
F3-LER+EOG(R)	6	0.93	0.37	0.72	0.45	0.88	0.67
P4-LER+EOG(R)	6	0.93	0.38	0.73	0.46	0.87	0.67
O1-LER+EOG(R)	6	0.93	0.38	0.73	0.46	0.89	0.68
Pz-LER+EOG(R)	6	0.93	0.40	0.75	0.49	0.85	0.68
C3-LER+EOG(R)	6	0.93	0.37	0.74	0.48	0.89	0.68
P4-CLE+EOG(R)	47	0.93	0.29	0.78	0.57	0.88	0.69
Cz-LER+EOG(R)	6	0.92	0.38	0.75	0.51	0.89	0.69
T5-LER+EOG(R)	6	0.93	0.39	0.75	0.50	0.88	0.69
Fz-LER+EOG(R)	6	0.93	0.40	0.75	0.50	0.90	0.69
F7-LER+EOG(R)	6	0.93	0.39	0.75	0.51	0.90	0.70
Fp1-LER+EOG(R)	3	0.93	0.34	0.80	0.50	0.93	0.70
Cz-CLE+EOG(R)	47	0.93	0.35	0.78	0.57	0.88	0.70
Fp2-LER+EOG(R)	3	0.93	0.39	0.80	0.51	0.91	0.71
T3-CLE+EOG(R)	47	0.93	0.37	0.79	0.58	0.88	0.71
F4-CLE+EOG(R)	47	0.93	0.38	0.79	0.59	0.88	0.71
F7-CLE+EOG(R)	47	0.94	0.40	0.80	0.60	0.87	0.72
P3-CLE+EOG(R)	47	0.93	0.40	0.80	0.60	0.87	0.72
T5-CLE+EOG(R)	47	0.94	0.40	0.80	0.60	0.88	0.72
Fz-CLE+EOG(R)	47	0.94	0.39	0.81	0.61	0.88	0.73
F3-CLE+EOG(R)	47	0.93	0.42	0.80	0.61	0.87	0.73
C3-CLE+EOG(R)	47	0.94	0.40	0.81	0.62	0.88	0.73
O1-CLE+EOG(R)	47	0.94	0.40	0.81	0.62	0.88	0.73
O2-CLE+EOG(R)	47	0.93	0.42	0.82	0.61	0.88	0.73
F8-CLE+EOG(R)	47	0.93	0.41	0.82	0.63	0.88	0.73
C4-CLE+EOG(R)	47	0.93	0.39	0.83	0.65	0.88	0.74
T4-CLE+EOG(R)	47	0.94	0.41	0.83	0.64	0.88	0.74
Pz-CLE+EOG(R)	47	0.94	0.42	0.83	0.64	0.88	0.74
T6-CLE+EOG(R)	47	0.94	0.41	0.83	0.65	0.88	0.74

Table 21: MASS-C1 (part 2/2) - Test data - Channel-Wise F1/dice scores (computed across subjects). This table displays all MASS-C1 evaluations on EOG(L) channel combinations. Please refer to Table 20 for MASS-C1 evaluations with EOG(R) channels. The displayed mean, standard deviation and majority vote scores represent summaries computed across both EOG(L) (this table) and EOG(R) (Table 20) channel combinations.

	Records	Wake	N1	N2	N3	REM	mean
F8-LER+EOG(L)	6	0.91	0.38	0.74	0.48	0.82	0.67
P3-LER+EOG(L)	6	0.93	0.36	0.71	0.44	0.90	0.67
O2-LER+EOG(L)	6	0.93	0.37	0.72	0.46	0.87	0.67
C4-LER+EOG(L)	6	0.91	0.40	0.74	0.49	0.84	0.68
Pz-LER+EOG(L)	6	0.92	0.41	0.75	0.49	0.84	0.68
T6-LER+EOG(L)	6	0.92	0.41	0.75	0.49	0.88	0.69
T4-LER+EOG(L)	6	0.93	0.38	0.75	0.48	0.91	0.69
C3-LER+EOG(L)	6	0.91	0.38	0.77	0.53	0.87	0.69
T3-LER+EOG(L)	6	0.93	0.39	0.76	0.50	0.90	0.70
P4-LER+EOG(L)	6	0.92	0.41	0.77	0.52	0.87	0.70
O1-LER+EOG(L)	6	0.93	0.39	0.77	0.51	0.90	0.70
F3-LER+EOG(L)	6	0.93	0.42	0.76	0.51	0.88	0.70
T5-LER+EOG(L)	6	0.92	0.42	0.79	0.56	0.87	0.71
F4-LER+EOG(L)	6	0.93	0.43	0.78	0.53	0.90	0.71
F8-CLE+EOG(L)	47	0.94	0.40	0.78	0.58	0.87	0.71
F7-LER+EOG(L)	6	0.93	0.40	0.79	0.58	0.88	0.72
T3-CLE+EOG(L)	47	0.94	0.43	0.78	0.56	0.88	0.72
T4-CLE+EOG(L)	47	0.94	0.42	0.79	0.57	0.87	0.72
Fz-LER+EOG(L)	6	0.93	0.44	0.79	0.55	0.89	0.72
O1-CLE+EOG(L)	47	0.94	0.41	0.79	0.58	0.88	0.72
T6-CLE+EOG(L)	47	0.94	0.41	0.79	0.59	0.88	0.72
Cz-CLE+EOG(L)	47	0.94	0.45	0.79	0.57	0.87	0.72
Cz-LER+EOG(L)	6	0.93	0.44	0.80	0.58	0.89	0.73
P4-CLE+EOG(L)	47	0.94	0.42	0.80	0.59	0.89	0.73
C4-CLE+EOG(L)	47	0.94	0.43	0.81	0.60	0.87	0.73
O2-CLE+EOG(L)	47	0.94	0.44	0.82	0.59	0.86	0.73
C3-CLE+EOG(L)	47	0.94	0.41	0.81	0.62	0.87	0.73
Fp1-LER+EOG(L)	3	0.94	0.43	0.83	0.56	0.91	0.73
Pz-CLE+EOG(L)	47	0.94	0.42	0.82	0.62	0.87	0.73
P3-CLE+EOG(L)	47	0.93	0.43	0.82	0.63	0.86	0.73
Fz-CLE+EOG(L)	47	0.94	0.43	0.81	0.61	0.87	0.73
T5-CLE+EOG(L)	47	0.94	0.44	0.83	0.63	0.87	0.74
F4-CLE+EOG(L)	47	0.93	0.45	0.83	0.63	0.86	0.74
F3-CLE+EOG(L)	47	0.94	0.47	0.83	0.63	0.85	0.74
F7-CLE+EOG(L)	47	0.94	0.48	0.83	0.63	0.86	0.75
Fp2-LER+EOG(L)	3	0.92	0.48	0.85	0.64	0.88	0.75
Mean [Tables 20 & 21]		0.93	0.40	0.78	0.55	0.88	0.71
Standard deviation [Tables 20 & 21]		0	0.03	0.04	0.07	0.02	0.03
Majority vote [Tables 20 & 21]	53	0.94	0.41	0.81	0.61	0.88	0.73

Table 22: MASS-C3 - Test data - Channel-Wise F1/dice scores (computed across subjects)

	Records	Wake	N1	N2	N3	REM	mean
C3-LER+EOG(L)	62	0.91	0.50	0.83	0.69	0.90	0.76
O2-LER+EOG(L)	62	0.91	0.50	0.82	0.69	0.90	0.77
C3-LER+EOG(R)	62	0.90	0.51	0.83	0.70	0.89	0.77
O2-LER+EOG(R)	62	0.91	0.51	0.83	0.70	0.90	0.77
F8-LER+EOG(L)	62	0.92	0.50	0.84	0.70	0.91	0.77
F8-LER+EOG(R)	62	0.92	0.51	0.85	0.72	0.90	0.78
T3-LER+EOG(L)	62	0.92	0.50	0.85	0.72	0.91	0.78
F7-LER+EOG(L)	62	0.92	0.52	0.85	0.72	0.91	0.78
Oz-LER+EOG(R)	62	0.90	0.52	0.86	0.75	0.89	0.78
P3-LER+EOG(R)	62	0.89	0.51	0.88	0.78	0.87	0.78
F7-LER+EOG(R)	62	0.92	0.53	0.85	0.73	0.90	0.79
Fp2-LER+EOG(L)	62	0.91	0.53	0.85	0.73	0.90	0.79
Cz-LER+EOG(L)	62	0.92	0.52	0.85	0.73	0.90	0.79
Cz-LER+EOG(R)	62	0.91	0.52	0.86	0.74	0.90	0.79
T3-LER+EOG(R)	62	0.92	0.51	0.86	0.74	0.90	0.79
Oz-LER+EOG(L)	62	0.91	0.53	0.86	0.74	0.90	0.79
Fz-LER+EOG(L)	62	0.92	0.53	0.85	0.73	0.91	0.79
P3-LER+EOG(L)	62	0.89	0.51	0.88	0.77	0.88	0.79
Fz-LER+EOG(R)	62	0.91	0.53	0.85	0.74	0.90	0.79
Fp2-LER+EOG(R)	62	0.91	0.55	0.86	0.74	0.90	0.79
Pz-LER+EOG(L)	62	0.92	0.53	0.86	0.74	0.90	0.79
Pz-LER+EOG(R)	62	0.92	0.53	0.86	0.74	0.89	0.79
T6-LER+EOG(L)	62	0.91	0.52	0.87	0.75	0.90	0.79
T6-LER+EOG(R)	62	0.91	0.53	0.87	0.75	0.90	0.79
T4-LER+EOG(R)	62	0.91	0.55	0.87	0.76	0.88	0.79
F3-LER+EOG(L)	62	0.91	0.53	0.87	0.75	0.90	0.79
F3-LER+EOG(R)	62	0.91	0.54	0.87	0.76	0.90	0.79
T5-LER+EOG(L)	62	0.92	0.53	0.88	0.76	0.90	0.80
O1-LER+EOG(L)	62	0.92	0.53	0.88	0.76	0.90	0.80
T4-LER+EOG(L)	62	0.91	0.55	0.88	0.76	0.89	0.80
C4-LER+EOG(R)	62	0.91	0.56	0.88	0.76	0.88	0.80
F4-LER+EOG(L)	62	0.92	0.54	0.88	0.76	0.90	0.80
T5-LER+EOG(R)	62	0.92	0.54	0.88	0.77	0.89	0.80
F4-LER+EOG(R)	62	0.92	0.55	0.88	0.77	0.89	0.80
P4-LER+EOG(R)	62	0.91	0.56	0.88	0.77	0.89	0.80
C4-LER+EOG(L)	62	0.91	0.55	0.88	0.77	0.89	0.80
O1-LER+EOG(R)	62	0.92	0.54	0.88	0.77	0.90	0.80
P4-LER+EOG(L)	62	0.91	0.55	0.88	0.77	0.89	0.80
Fp1-LER+EOG(R)	62	0.91	0.55	0.88	0.77	0.90	0.80
Fp1-LER+EOG(L)	62	0.91	0.55	0.88	0.76	0.90	0.80
Mean		0.91	0.53	0.86	0.74	0.90	0.79
Standard deviation		0	0.02	0.02	0.02	0.01	0.01
Majority vote	62	0.93	0.54	0.87	0.75	0.91	0.80

Table 23: SVUH - Test data - Channel-Wise F1/dice scores (computed across subjects)

	Records	Wake	N1	N2	N3	REM	mean
C4-A1+EOG(L)	25	0.78	0.37	0.77	0.66	0.85	0.69
C3-A2+EOG(R)	25	0.80	0.36	0.80	0.79	0.86	0.72
C3-A2+EOG(L)	25	0.79	0.36	0.81	0.81	0.87	0.73
C4-A1+EOG(R)	25	0.80	0.38	0.81	0.81	0.86	0.73
Mean		0.79	0.37	0.80	0.77	0.86	0.72
Standard deviation		0	0.01	0.02	0.06	0.01	0.02
Majority vote	25	0.80	0.37	0.81	0.78	0.88	0.73

Table 24: DOD-H - Test data - Channel-Wise F1/dice scores (computed across subjects)

	Records	Wake	N1	N2	N3	REM	mean
FP2-F4+EOG2	25	0.83	0.47	0.85	0.79	0.89	0.77
FP2-F4+EOG1	25	0.83	0.48	0.85	0.80	0.89	0.77
FP1-F3+EOG2	25	0.84	0.50	0.85	0.79	0.90	0.78
FP1-F3+EOG1	25	0.83	0.51	0.86	0.80	0.91	0.78
FP1-M2+EOG1	25	0.88	0.56	0.83	0.74	0.92	0.79
FP1-M2+EOG2	25	0.88	0.55	0.83	0.76	0.91	0.79
FP1-O1+EOG1	25	0.88	0.57	0.83	0.76	0.90	0.79
F3-F4+EOG1	25	0.85	0.52	0.86	0.81	0.92	0.79
FP1-O1+EOG2	25	0.89	0.57	0.83	0.76	0.90	0.79
F3-M2+EOG1	25	0.88	0.58	0.83	0.75	0.93	0.79
F3-F4+EOG2	25	0.87	0.51	0.87	0.82	0.90	0.79
FP2-O2+EOG1	25	0.88	0.58	0.84	0.77	0.91	0.79
FP2-M1+EOG1	25	0.88	0.58	0.84	0.76	0.92	0.80
F3-O1+EOG1	25	0.89	0.58	0.84	0.76	0.91	0.80
FP2-M1+EOG2	25	0.89	0.59	0.84	0.76	0.92	0.80
F4-M1+EOG2	25	0.89	0.58	0.84	0.76	0.92	0.80
FP2-O2+EOG2	25	0.89	0.59	0.84	0.76	0.91	0.80
F3-M2+EOG2	25	0.88	0.58	0.85	0.77	0.92	0.80
F4-O2+EOG1	25	0.89	0.58	0.84	0.78	0.91	0.80
F4-M1+EOG1	25	0.88	0.59	0.85	0.77	0.92	0.80
C3-M2+EOG1	25	0.87	0.59	0.85	0.77	0.92	0.80
F4-O2+EOG2	25	0.89	0.59	0.85	0.77	0.91	0.80
F3-O1+EOG2	25	0.90	0.60	0.85	0.78	0.92	0.81
C3-M2+EOG2	25	0.88	0.58	0.86	0.79	0.93	0.81
Mean		0.87	0.56	0.85	0.77	0.91	0.79
Standard deviation		0	0.04	0.01	0.02	0.01	0.01
Majority vote	25	0.91	0.60	0.87	0.79	0.94	0.82

Table 25: DOD-O - Test data - Channel-Wise F1/dice scores (computed across subjects)

	Records	Wake	N1	N2	N3	REM	mean
C3-M2+EOG2	55	0.88	0.51	0.81	0.70	0.88	0.76
F3-M2+EOG2	55	0.88	0.50	0.82	0.69	0.91	0.76
C4-M1+EOG1	55	0.89	0.51	0.83	0.71	0.89	0.77
F3-M2+EOG1	55	0.88	0.50	0.83	0.70	0.92	0.77
C4-M1+EOG2	55	0.87	0.50	0.84	0.71	0.91	0.77
F3-F4+EOG2	55	0.85	0.47	0.86	0.74	0.91	0.77
C3-M2+EOG1	55	0.89	0.51	0.83	0.71	0.90	0.77
F3-O1+EOG2	55	0.87	0.49	0.84	0.73	0.91	0.77
F4-O2+EOG2	55	0.87	0.49	0.84	0.73	0.92	0.77
F4-O2+EOG1	55	0.88	0.50	0.84	0.72	0.91	0.77
F3-F4+EOG1	55	0.87	0.47	0.86	0.74	0.92	0.77
F3-O1+EOG1	55	0.88	0.49	0.85	0.73	0.92	0.77
O1-M2+EOG2	55	0.87	0.49	0.85	0.76	0.89	0.77
O2-M1+EOG1	55	0.88	0.50	0.85	0.76	0.89	0.78
O1-M2+EOG1	55	0.88	0.50	0.86	0.76	0.90	0.78
O2-M1+EOG2	55	0.87	0.50	0.86	0.77	0.90	0.78
Mean		0.88	0.50	0.84	0.73	0.91	0.77
Standard deviation		0	0.01	0.01	0.03	0.01	0.01
Majority vote	55	0.90	0.52	0.86	0.74	0.92	0.79

References

[1] Zhang, G. Q. *et al.* The National Sleep Research Resource: Towards a sleep data commons. *Journal of the American Medical Informatics Association* **25**, 1351–1358 (2018).

[2] Bakker, J. P. *et al.* Gastric banding surgery versus continuous positive airway pressure for obstructive sleep apnea: A randomized controlled trial. *American Journal of Respiratory and Critical Care Medicine* **197**, 1080–1083 (2018).

[3] Rosen, C. L. *et al.* Prevalence and risk factors for sleep-disordered breathing in 8- to 11-year-old children: Association with race and prematurity. *Journal of Pediatrics* **142**, 383–389 (2003).

[4] Redline, S. *et al.* The familial aggregation of obstructive sleep apnea. *American Journal of Respiratory and Critical Care Medicine* **151**, 682–687 (1995).

[5] Marcus, C. L. *et al.* A randomized trial of adenotonsillectomy for childhood sleep apnea. *New England Journal of Medicine* **368**, 2366–2376 (2013).

[6] Redline, S. *et al.* The childhood adenotonsillectomy trial (chat): Rationale, design, and challenges of a randomized controlled trial evaluating a standard surgical procedure in a pediatric population. *Sleep* **34**, 1509–17 (2011).

[7] Rosen, C. L. *et al.* A multisite randomized trial of portable sleep studies and positive airway pressure autotitration versus laboratory-based polysomnography for the diagnosis and treatment of obstructive sleep apnea: The HomePAP study. *Sleep* **35**, 757–677 (2012).

[8] Chen, X. *et al.* Racial/ethnic differences in sleep disturbances: The Multi-Ethnic Study of Atherosclerosis (MESA). *Sleep* **38**, 877–88 (2015).

[9] Blackwell, T. *et al.* Associations between sleep architecture and sleep-disordered breathing and cognition in older community-dwelling men: The osteoporotic fractures in men sleep study. *Journal of the American Geriatrics Society* **59**, 2217–2225 (2011).

[10] Song, Y. *et al.* Relationships between sleep stages and changes in cognitive function in older men: The MrOS sleep study. *Sleep* **38**, 411–421 (2015).

[11] Ghassemi, M. M. *et al.* You snooze, you win: the PhysioNet/Computing in Cardiology challenge 2018. In *2018 Computing in Cardiology Conference (CinC)*, vol. 45, 1–4 (2018).

[12] Goldberger, A. L. *et al.* PhysioBank, PhysioToolkit, and PhysioNet: Components of a new research resource for complex physiologic signals. *Circulation* **101**, e215–e220 (2000).

[13] Kemp, B., Zwinderman, A. H., Tuk, B., Kamphuisen, H. A. C. & Oberye, J. J. L. Analysis of a sleep-dependent neuronal feedback loop: the slow-wave microcontinuity of the EEG. *IEEE Transactions on Biomedical Engineering* **47**, 1185–1194 (2000).

[14] Quan, S. F. *et al.* The Sleep Heart Health Study: Design, rationale, and methods. *Sleep* **20**, 1077–1085 (1997).

[15] Cummings, S. R. *et al.* Appendicular Bone Density and Age Predict Hip Fracture in Women. *JAMA: The Journal of the American Medical Association* **263**, 665–668 (1990).

[16] Spira, A. P. *et al.* Sleep-disordered breathing and cognition in older women. *Journal of the American Geriatrics Society* **56**, 45–50 (2008).

[17] Khalighi, S., Sousa, T., dos Santos, J. M. & Nunes, U. ISRUC-Sleep: A comprehensive public dataset for sleep researchers. *Computer Methods and Programs in Biomedicine* **124**, 180–192 (2016).

[18] O'Reilly, C., Gosselin, N., Carrier, J. & Nielsen, T. Montreal archive of sleep studies: An open-access resource for instrument benchmarking and exploratory research. *Journal of Sleep Research* **23**, 628–635 (2014).

[19] Guillot, A., Sauvet, F., During, E. H. & Thorey, V. Dreem open datasets: Multi-scored sleep datasets to compare human and automated sleep staging. *IEEE Transactions on Neural Systems and Rehabilitation Engineering* **28**, 1955–1965 (2019).

[20] Arnal, P. J. *et al.* The Dreem Headband as an alternative to polysomnography for EEG signal acquisition and sleep staging. *bioRxiv* (2019). URL `https://www.biorxiv.org/content/early/2019/06/10/662734`. `https://www.biorxiv.org/content/early/2019/06/10/662734.full.pdf`.

[21] Thorey, V., Hernandez, A. B., Arnal, P. J. & During, E. H. AI vs humans for the diagnosis of sleep apnea (2019). `arXiv:1906.09936`.

[22] Cribari-Neto, F. & Zeileis, A. Beta regression in R. *Journal of Statistical Software* (2010).

[23] R Core Team. *R: A Language and Environment for Statistical Computing*. R Foundation for Statistical Computing, Vienna, Austria (2019). URL `https://www.R-project.org/`.

[24] Hofmann, H., Wickham, H. & Kafadar, K. Letter-value plots: Boxplots for large data. *Journal of Computational and Graphical Statistics* **26**, 469–477 (2017). URL `https://doi.org/10.1080/10618600.2017.1305277`. `https://doi.org/10.1080/10618600.2017.1305277`.

[25] Anthony, L. F. W., Kanding, B. & Selvan, R. Carbontracker: Tracking and predicting the carbon footprint of training deep learning models. In *ICML Workshop on Challenges in Deploying and monitoring Machine Learning Systems* (2020).

[26] Perslev, M., Jensen, M., Darkner, S., Jennum, P. J. & Igel, C. U-Time: A fully convolutional network for time series segmentation applied to sleep staging. In *Advances in Neural Information Processing Systems (NeurIPS) 32*, 4415–4426 (2019).

[27] Iber, C. & AASM. *The AASM manual for the scoring of sleep and associated events: rules, terminology and technical specifications* (American Academy of Sleep Medicine, Westchester, IL, 2007).

[28] Kinga, D. P. & Ba, J. L. Adam: A method for stochastic optimization. In *International Conference on Learning Representations (ICLR)* (2015).

[29] Ronneberger, O., Fischer, P. & Brox, T. U-Net: Convolutional networks for biomedical image segmentation. In *Medical Image Computing and Computer-Assisted Intervention (MICCAI)*, vol. 9351 of *LNCS*, 234–241 (Springer, 2015).

[30] Odena, A., Dumoulin, V. & Olah, C. Deconvolution and checkerboard artifacts. *Distill* (2016).

[31] Ioffe, S. & Szegedy, C. Batch normalization: Accelerating deep network training by reducing internal covariate shift. In *International Conference on Machine Learning (ICML)*, 448–456 (2015).

[32] Yu, F. & Koltun, V. Multi-scale context aggregation by dilated convolutions. In *International Conference on Learning Representations (ICLR)* (2016).

[33] Clevert, D.-A., Unterthiner, T. & Hochreiter, S. Fast and accurate deep network learning by exponential linear units (ELUs). In *International Conference on Learning Representations (ICLR)* (2016).

[34] Virtanen, P. *et al.* Scipy 1.0-fundamental algorithms for scientific computing in Python. *Nature Methods* **17**, 261–272 (2020).
